# *Sirt6*-mediated epigenetic modification of DNA accessibility is essential for *Pou2f3*-induced thymic tuft cell development

**DOI:** 10.1038/s42003-022-03484-9

**Published:** 2022-06-06

**Authors:** Qian Zhang, Jiayu Zhang, Tong Lei, Zhanfeng Liang, Xue Dong, Liguang Sun, Yong Zhao

**Affiliations:** 1 State Key Laboratory of Membrane Biology, Institute of Zoology, Chinese Academy of Sciences, Beijing, China; 2 University of Chinese Academy of Sciences, Beijing, China; 3 National-local Joint Engineering Laboratory of Animal Models for Human Diseases, Institute of Translational Medicine, the First Hospital, Jilin University, Changchun, Jilin, China; 4 Institute for Stem Cell and Regeneration, Chinese Academy of Sciences, Beijing, China

**Keywords:** Thymus, Lymphocyte differentiation

## Abstract

Thymic epithelial cells (TECs) are essential for the production of self-tolerant T cells. The newly identified thymic tuft cells are regulated by *Pou2f3* and represent important elements for host type 2 immunity. However, epigenetic involvement in thymic tuft cell development remains unclear. We performed single-cell ATAC-seq of medullary TEC (mTEC) and established single-cell chromatin accessibility profiling of mTECs. The results showed that mTEC III cells can be further divided into three groups (Late Aire 1, 2, and 3) and that thymic tuft cells may be derived from Late Aire 2 cells. *Pou2f3* is expressed in both Late Aire 2 cells and thymic tuft cells, while *Pou2f3*-regulated genes are specifically expressed in thymic tuft cells with simultaneous opening of chromatin accessibility, indicating the involvement of epigenetic modification in this process. Using the epigenetic regulator *Sirt6*-defect mouse model, we found that *Sirt6* deletion increased Late Aire 2 cells and decreased thymic tuft cells and Late Aire 3 cells without affecting *Pou2f3* expression. However, *Sirt6* deletion reduced the chromatin accessibility of *Pou2f3*-regulated genes in thymic tuft cells, which may be caused by *Sirt6*–mediated regulation of *Hdac9* expression. These data indicate that epigenetic regulation is indispensable for *Pou2f3*-mediated thymic tuft cell development.

## Abstract (web summary)

Thymic epithelial cells (TECs) are essential for the production of self-tolerant T cells. Single-cell ATAC-seq of medullary TECs suggests that Sirt6-mediated epigenetic regulation may regulate the development of thymic tuft cells from these TECs.

## Introduction

The thymus is essential for the education and maturation of functionally competent and self-tolerant T cells, and diversely specialized thymic epithelial cells (TECs), including cortical TECs (cTECs) and medullary TECs (mTECs), play critical roles in these processes^1^. cTECs mainly coordinate the early stages and positive selection of thymocytes, while mTECs primarily carry out the later steps and negative selection of thymocytes (reviewed in ref. ^2,3^). The heterogeneity of mTECs in mice and humans has recently been recognized with lineage-tracing mouse models, bulk RNA sequencing (RNA-seq), and single-cell RNA-seq (scRNA-seq) assays^4–8^. Abramson and Amit’s teams identified four mTEC subgroups, including mTEC I-IV. In the adult thymus, immature Ccl21^+^Aire^−^ mTEC I cells are considered the earliest mTEC cluster, which recruits CCR7^+^ thymocytes to migrate from the thymic cortex region into the medulla region. Mature Aire^+^ mTEC II cells, as a functional mature mTEC cluster, randomly express tissue-restricted antigens to eliminate autoreactive thymocytes and contribute to the establishment of central immune tolerance. The corneocyte-like post-Aire expression KRT10^+^ mTEC III population is a terminally differentiated mTEC cluster that is also known as the late Aire stage, and it downregulates the expression of Aire, CD80, and MHC class II. The double cortin-like kinase 1 (*Dclk1*)^+^ and/or transient receptor potential cation channel subfamily M member 5 (*Trpm5*)^+^ mTEC IV population is a recently identified terminally differentiated mTEC cluster^4,5^. The mTEC IV population is also called thymic tuft cells because it expresses a group of canonical taste transduction pathway-related genes, which are similar to intestinal tuft cells^4,5,9,10^. These thymic tuft cells account for 3–10% of the mTECs in the mouse and human thymus^4,5^. These observations are consistent with previous studies showing that a subset of eGFP^+^ mTECs express α-gustducin (GNAT3), Trpm5, and phospholipase Cβ2 (PLCβ2) and are present as terminally differentiated epithelia in Hassall corpuscle–like clusters in Chat-eGFP BAC transgenic mice^11^. These thymic tuft cells are also detectable in BAC transgenic mice expressing CreERT2 from the *Tas2r143* promoter^12^. The expression of several bitter taste–associated receptors (*Tas2r105, Tas2r108*, and *Tas2r131*) in mTECs has been reported in taste receptor *Tas2r131* reporter mice^13^. Recently, in vivo fate mapping and lineage-tracing results have shown that mTEC IV cells (thymic tuft cells) are derived from mTEC II and/or III cells or a common ancestor through an Aire-expressing stage and/or an Aire-independent pathway^4,5^. For the convenience of discussion, we use the term thymic tuft cells to describe the tuft cell-like mTEC IV subgroup.

Importantly, these thymic tuft cells express high levels of the type 2 cytokine IL-25, MHC class II molecules, and associated antigen presentation genes^4,5^. *Pou2f3*-deficient mice showed a marked decrease in the number of TCRβ^int^CD1d^+^IL-4^+^ type 2 invariant natural killer T (NKT2) cells and IL-4-dependent EOMES^+^CD8^+^ thymocytes and an increased frequency of thymic Lin^−^TCR^−^CD127^+^GATA3^+^ ILC2s without a significant impact on CD4^−^CD8^−^, CD4^+^CD8^+^, CD4^−^CD8^+^, and CD4^+^CD8^−^ cells in the thymus, companying with an absence of thymic tuft cells^4,5^. Thus, thymic tuft cells are immunologically important, highly differentiated epithelial cells in the thymic medulla^4,5^. POU class 2 homeobox 3 (*Pou2f3*, *Skn-1a*, and *Oct11*)- and *Trpm5*-deficient mice show a specific loss of thymic tuft cells^4,5^, indicating that taste transduction pathway genes, such as *Pou2f3* and *Trpm5*, are required for the development and function of thymic tuft cells. However, the epigenetic regulation of thymic tuft cell development has not been clarified. In the present study, we performed scRNA-seq and scATAC-seq analyses of mouse TECs and applied a TEC-specific deletion of the *Sirt6* mouse model to define the routine development of thymic tuft cells, the *Pou2f3*-regulated gene expressing landscape in different mTEC subsets and the roles of *Sirt6* in the development of thymic tuft cells. Our results showed that thymic tuft cells may be derived from one subgroup of mTEC III cells (Late Aire 2) and that epigenetic regulation is indispensable for the development of *Pou2f3*-mediated thymic tuft cells. This work advances our understanding of epigenetic regulation of the development of mTECs, especially thymic tuft cells, and provides an unbiased scATAC-seq resource to study mTEC heterogeneity.

## Results

### Single-cell chromatin accessibility profiling of mouse mTECs

The single-cell assay for transposase-accessible chromatin with sequencing (scATAC-seq) method, which identifies active DNA regulatory elements by transposition of sequencing adapters into accessible chromatin in cells, has enabled mapping of cell-to-cell variability and epigenomic profiling of cells even in low-cell-number samples^14–16^. To investigate the chromatin accessibility characteristics of thymic tuft cells, 200000 CD45^-^EpCAM^+^ TECs were sorted from 4-week-old wild-type mice for scATAC-seq assays (10X Genomics Chromium) (Suppl. Fig. 1). After filtering, 4739 mTECs with high-quality data were analyzed (Method, Suppl. Fig. 2). To determine the cell type annotation of mTEC scATAC-seq, we first performed unbiased clustering on the downloaded scRNA-seq of WT^17^. According to the different molecular characteristics of mTEC clusters based on scRNA-seq assays^5^(Suppl. Figs. 3, 4), mTECs can be divided into 4 subgroups: (1) mTEC I highly expressed the indicator genes *Pdpn*, *Ccl21*, *Ly6a*, *Itga6*, and *Itga4*; (2) mTEC II highly expressed *Aire*, *Fezf2*, *Cd80*, *Cd40*, and *Cd74*; (3) mTEC III highly expressed *Pigr*, *Ly6d*, *Ivl*, and *Krt10*; and (4) thymic tuft cells highly expressed the indicator genes *Lrmp*, *Gnat3*, *Dclk1*, *Trpm5*, *L1cam*, and *Avil*.

**Fig. 1:**
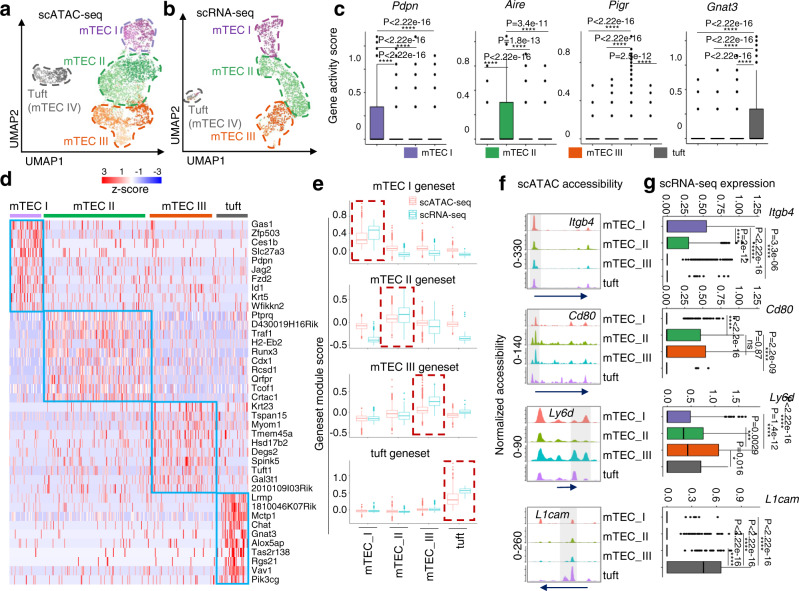
Chromatin accessibility profiling of mTECs was established according to the known single-cell transcriptional profile. **a** UMAP visualization of scATAC-seq profiles of mTECs. Cell types are circled with dotted lines of different colors. **b** UMAP visualization of scRNA-seq profiles of mTECs. Cell types are circled with dotted lines of different colors. **c** Boxplots show marker genes for each mTEC cluster by gene accessibility matrix. Color represents specific clusters of mTECs. mTEC I: *n* = 638, mTEC II: *n* = 1982, mTEC III: *n* = 1262, thymic tuft cells: *n* = 495. **d** Heatmap of the gene expression profile of defined mTEC populations in scATAC-seq was generated by the top ten marker genes from the gene activity matrix. The color scale indicates the level of gene activity. **e** Marker genes of each mTEC population calculated by scRNA-seq were added to both scRNA-seq and scATAC-seq. Gene set module scores in scRNA-seq (blue) and scATAC-seq (red) were compared with boxplots. **f** Genome tracks of aggregate scATAC-seq data show marker chromatin accessibility peaks. The arrow indicates the length and direction of the genes detected by scATAC-seq. **g** Boxplots show normalized expression of the marker genes for each mTEC cluster in scRNA-seq data. mTEC I: *n* = 364, mTEC II: *n* = 991, mTEC III: *n* = 458, thymic tuft cells: *n* = 62. The statistics was determined using Wilcoxon signed-rank test. ****p < 0.001.

To identify the genes accessible in each cell type, a gene activity matrix was established to serve as pseudoexpression data to identify the known mTEC subsets. Projection of gene expression (scRNA-seq) and gene activity scores (scATAC-seq) in the coembedded space shows that distinct clustering of unique cell types is preserved and that scATAC-seq and scRNA-seq cells of the same population cluster together^16,18–20^. scATAC-seq profiles were obtained by unbiased clustering on all peaks of WT mTECs using Signac, and the corresponding cell population annotation was predicted based on the gene activity matrix and coembedment analysis (Suppl. Fig. 5). Furthermore, we checked the subgroup-specific gene sets from scRNA-seq at different resolutions on scATAC-seq to determine the final scATAC-seq profile, which also proved the correlation between the identified cell type and the predicted id was consistent (Fig. 1a, b and Suppl. Figs. 6,  7).

Based on the expression levels of marker genes and gene sets, we identified four mTEC clusters (Fig. 1a), which showed clustering similar to that of the scRNA-seq data of mTECs^5^. Gene activity matrix data (pseudogene expression) enabled us to identify the highly expressed marker genes in different mTEC subgroups, as were recently reported^5^: *Pdpn* in mTEC I, *Aire* in mTEC II, *Pigr* in mTEC III, and *Gnat3* in thymic tuft cells (Fig. 1c and Supplementary Data 1). We then enriched the highly accessible genes in different mTEC subgroups based on scATAC-seq data (Fig. 1d). After analyzing the specific gene set of each mTEC cluster, we found that the chromatin accessibility detected by scATAC-seq was highly consistent with the RNA expression detected by scRNA-seq in each cluster (Fig. 1e and Supplementary Data 1). Additionally, we found that the gene set of thymic tuft cells was more specific than the mTEC I-III gene set in terms of transcriptional signature and chromatin accessibility (Fig. 1e and Supplementary Data 1). To determine whether cell-type-specific chromatin accessibility correlated with gene expression, we compared chromatin accessibility and mRNA expression in the known characteristic genes of each mTEC subgroup, including *Itgb4* in mTEC I, *CD80* in mTEC II, *Ly6d* in mTEC III, and *L1cam* in thymic tuft cells. The openness of the target gene chromatin accessibility (Fig. 1f) was nicely associated with its mRNA expression in all mTEC subgroups (Fig. 1g, Suppl. Fig. 8, and Supplementary Data 1), indicating that there was a positive correlation between chromatin accessibility and transcriptional level in the same cell types. Analysis of ATAC-seq profiles revealed that mTEC I and thymic tuft cells possessed more open chromatin regions than mTEC II and III cells, as determined by the ATAC peak numbers (Suppl. Fig. 9). The distribution of the specific opened peaks in mTEC I and thymic tuft cells was located mainly in promoter and distal regions (Suppl. Fig. 9), which is consistent with the association of distal chromatin elements with the developing cell type specificity as reported^21^. These results collectively proved the consistency between chromatin accessibility obtained by scATAC-seq assays and its mRNA expression obtained by scRNA-seq assays in mTEC clusters, and they also indicated the rich diversity of mTECs is accompanied by global remodeling of the chromatin accessibility landscape.

### Three subpopulations in the mTEC III subgroup

**Fig. 2:**
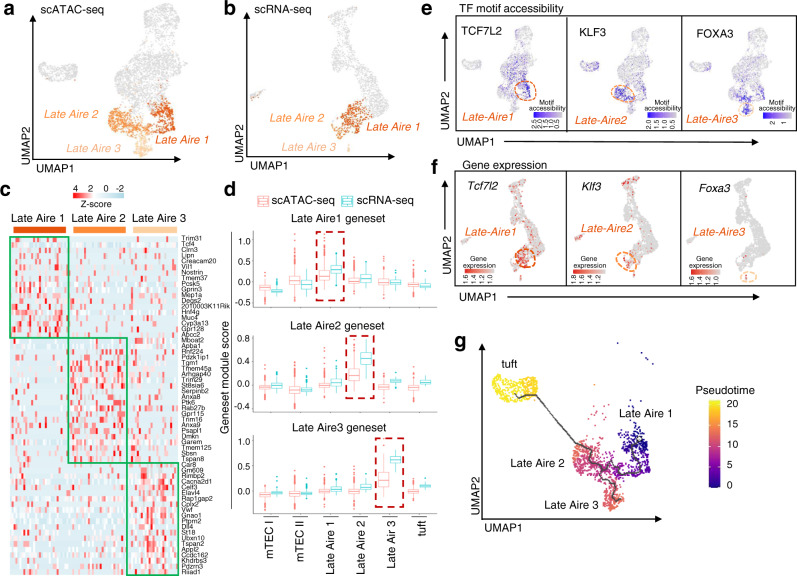
Chromatin accessibility, gene expression, and differentiation trajectory of three newly identified mTEC III subsets. UMAP visualization of scATAC-seq profiles (**a**) and scRNA-seq profiles (**b**). Late Aire 1, Late Aire 2, and Late Aire 3 cells labeled with different colors are defined by the different chromatin accessibility in scATAC-seq and scRNA-seq profiles. **c** Heatmap of the gene expression profile of late Aire 1–3 populations in scATAC-seq was generated by the top 20 marker genes from the gene activity matrix. The color scale indicates the level of gene activity. **d** Marker genes of each Late Aire population calculated by scATAC-seq were added to both scRNA-seq and scATAC-seq. The gene set module scores of Late Aire 1-3 in scRNA-seq (blue) and scATAC-seq (red) were compared in boxplots. **e** UMAP visualization of TF motif accessibility for the indicated TFs in Late Aire 1, Late Aire 2, and Late Aire 3 cells. The blue scale in the UMAP plot indicates the level of TF motif accessibility in scATAC-seq analyzed by chromVAR. **f** UMAP visualization of scRNA-seq colored based on the expression of the indicated TFs in Late Aire 1, Late Aire 2, and Late Aire 3 cells. The red scale in UMAP plot indicate the level of gene expression in scRNA-seq. **g** UMAP visualization of scATAC-seq subsets of Late Aire 1, Late Aire 2, Late Aire 3, and thymic tuft cells. Colored based on the pseudotime, with blue corresponding to early cells and yellow corresponding to thymic tuft cells.

mTECs express autoantigens instantaneously and have many heterogeneous populations^22^, and although the properties have been studied, they are not completely understood. It is well known that fine cell types can be distinguished by different resolutions in single-cell sequencing^22^. mTEC III, which shows significant heterogeneity in scATAC-seq, can be divided into three subgroups even at different resolutions (Fig. 1a and Suppl. Figs. 10,  11). Therefore, we further divided mTEC II into Late Aire 1, Late Aire 2, and Late Aire 3 at resolution 1 (Fig. 2a, b). By analyzing the chromatin accessibility and transcriptome of the three identified mTEC III clusters, we found that Late Aire 1 (*Hnf4g, Trim31, Nos2*, and *Gpa33*), Late Aire 2 (*Tspan8, Ly6d, Tmem45a*, and *Psapl1*), and Late Aire 3 (*Cd177, Fam174b, Car8*, and *Gm609*) were relatively independent mTEC subgroups with distinct chromatin accessibility profiles (Fig. 2c and Suppl. Fig. 12). This observation was consistent with a recent report showing that mTECs might have as many as 14 subgroups as analyzed by scRNA-seq^22^, and Late Aire 1 corresponded to C7 (*Gpa33* and *Nos2*), Late Aire 2 corresponded to C8 (*Tspan8* and *Ly6d*), and Late Aire 3 corresponded to C9 (*Cd177* and *Car8*). According to the marker genes determined by scATAC-seq, we found the corresponding three groups of cells by scRNA-seq (Suppl. Fig. 13). The highly expressed genes in Late Aire 1 to 3 of scRNA-seq were used to perform GO enrichment analyses (Suppl. Fig. 14), and calcium ion-regulated endocytosis was specifically enriched in Late Aire 3 cells. A new type of EPCAM^+^ cells has been identified in humans as neuro TECs^8,23^, which resemble neuroendocrine cells. In mice, Late Aire 3 cells did not express the markers *Neurog1* and *Neurod1* of neural TECs (Suppl. Fig. 15), indicating that Late Aire 3 cells might be a special subset of terminally differentiated cells. At the same time, the difference in the gene set module score in one of the Late Aire types (i.e., 1–3) further indicated the heterogeneity of mTEC III, as demonstrated by the scATAC-seq and scRNA-seq data (Fig. 2d and Supplementary Data 2). Compared with Late Aire 1 and 2, the major alteration of increased chromatin accessibility in Late Aire 3 was found in other intron regions (Suppl. Fig. 16).

Chromatin accessibility analysis can be used to identify candidate transcriptional regulators of each cell state or cell type during cell differentiation^24^. We measured chromatin accessibility at cis-elements sharing a transcription factor (TF)-binding motif using the chromVAR analysis approach^25^ and cross-matched the data among enriched highly activated motifs, scATAC-seq data, and scRNA-seq data to identify the key transcription factors in each cell of the mTEC III subgroup. We found that Late Aire 1–3 were specifically regulated by different transcription factors (Suppl. Fig. 17). Late Aire 1 was predominantly and specifically regulated by TCF7L2, Late Aire 2 was mainly regulated by KLF3, and Late Aire 3 was selectively regulated by FOXA3 (Fig. 2e). In parallel, through the analysis of scRNA-seq data, we found that the genes *Tcf7l2* and *Foxa3* were specifically expressed in Late Aire 1 and Late Aire 3, respectively, while *Klf3* was also partially expressed in mTEC I and thymic tuft cells in addition to Late Aire 2 (Fig. 2f).

Because lineage-tracing studies by two independent research teams showed that more than half of thymic tuft cells arise from mature mTEC^high^ cells^4,5^, *Klf3* was partially expressed in thymic tuft cells and KLF3 regulatory motifs were partially located in thymic tuft cells, we speculated that Late Aire 2 cells have a strong developmental correlation with thymic tuft cells. The development path of thymic tuft cells could not be obtained in all cells based on scATAC-seq, which may be caused by the large difference in gene expression between tufts and other mTECs (Suppl. Fig. 18). Hence, we used scATAC-seq to reconstruct cellular developmental trajectories of the Late Aire 1, Late Aire 2, Late Aire 3, and thymic tuft cells in an unbiased manner^14^ to further prove their developmental relevance. Differentiation pseudotime analysis and RNA velocity analysis showed that the Late Aire 2 subgroup had a certain developmental correlation with thymic tuft cells, which was also verified by scRNA-seq (Fig. 2g and Suppl. Figs. 19, 20). Thus, we speculate that the Late Aire 2 subgroup may represent a crossroad for differentiation into either Late Aire 3 or thymic tuft cells.

### Increased chromatin accessibility is essential for *Pou2f3*-induced thymic tuft cell development

**Fig. 3:**
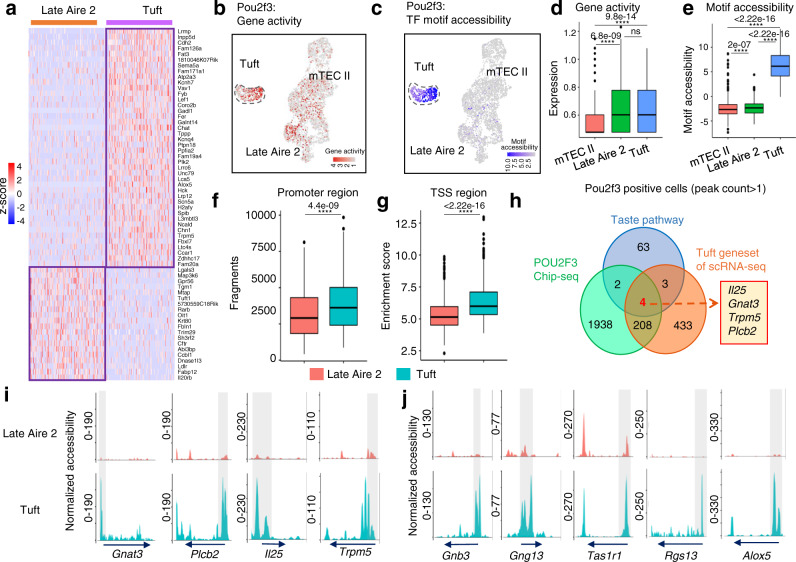
Pou2f3 but not its target genes are expressed before Late Aire 2, and chromatin opening is required for target gene expression in thymic tuft cells. **a** Heatmap of differentially accessible genes between Late Aire 2 cells and thymic tuft cells in scATAC-seq. The color scale indicates the level of gene activity. **b** UMAP visualization of the gene expression of *Pou2f3* in scRNA-seq. **c** UMAP visualization of the motif accessibility score of *Pou2f3* in scATAC-seq. **d** Gene expression of *Pou2f3* in *Pou2f3*^+^ mTEC subsets in scATAC-seq was compared in a boxplot. *Pou2f3*^+^ mTEC II: *n* = 499, *Pou2f3*^+^ Late Aire 2 cells: *n* = 210, *Pou2f3*^+^ thymic tuft cells: *n* = 297. **e** Motif accessibility score of *Pou2f3* in *Pou2f3*^+^ mTEC subsets in scATAC-seq was compared in a boxplot. Boxplots show the fragments at the promoter region (**f**) and TSS enrichment score (**g**) for Late Aire 2 cells (pink) and thymic tuft cells (blue). Late Aire 2 cells: *n* = 551, thymic tuft cells: *n* = 495. **h** Taste pathway-related gene (blue), *Pou2f3* directly regulated gene sets (green), and tuft gene sets of scRNA-seq (orange) are calculated in a Venn diagram. The overlapping genes among them are shown on the right. The chromatin accessibility peaks of overlapping genes (**i**) and tuft-associated genes (**j**) are shown in scATAC-seq tracks. The arrow indicates the length and direction of the genes detected by scATAC-seq. The statistics was determined using Wilcoxon signed-rank test. ****p < 0.001.

To further explore the developmental relationship between Late Aire 2 cells and thymic tuft cells, we analyzed their chromatin accessibility profiles by using scATAC-seq. The results showed that Late Aire 2 cells and thymic tuft cells had relatively specific chromatin accessibility profiles (Fig. 3a). *Pou2f3*, a well-known key transcription factor for the development of thymic tuft cells, may be accessible not only in thymic tuft cells but also in mTEC III^5^. Accordingly, we calculated the accessibility of *Pou2f3* in scATAC-seq using pseudoexpression data (Fig. 3b). In mTEC I, Late Aire 1, and Late Aire 3, the peak count of *Pou2f3* was less than 1 in 75% of the cells (upper edge of boxplot), while in both mTEC II and Late Aire 2, the peak count of *Pou2f3* was more than 1 in 50% of the cells (boxplot median); moreover, *Pou2f3* was accessible in 75% of the cells (boxplot lower margin) at a peak count of more than 1 in thymic tuft cells. This finding indicates that *Pou2f3* is mainly accessible in mTEC II, Late Aire 2, and thymic tuft cells (Suppl. Fig. 19a). Among these three populations, *Pou2f3* had the highest accessibility in thymic tuft cells (Suppl. Figs. 21,  22a). Except for thymic tuft cells, *Pou2f3* was accessible on each exon in Late Aire 2 cells, while other populations mainly opened in exon 1 and exon 2 of *Pou2f3* (Suppl. Fig. 22b). However, scRNA-seq data showed that the expression of *Pou2f3* was detected only in Late Aire 2 cells and thymic tuft cells (Suppl. Fig. 23). The expression of *Pou2f3* was not limited to thymic tuft cells, further suggesting that Late Aire 2 may have a stronger developmental correlation with thymic tuft cells than mTEC II. However, the peaks bound by *Pou2f3* were detectable specifically in thymic tuft cells, while almost no detectable motif accessibility was detected in other mTEC subgroups (Fig. 3c and Suppl. Fig. 24), indicating the motif accessibility of *Pou2f3* is critical for the differentiation of thymic tuft cells.

Because the majority of mTECs did not express *Pou2f3*, we selected *Pou2f3*^+^ mTEC subsets for the motif accessibility analysis. The accessibility region of *Pou2f3*^+^ cells in each population did not change compared to that of all cells (Suppl. Figs. 19c,  25). As mentioned earlier, there was no significant difference (*P* > 0.05) in *Pou2f3* accessibility between *Pou2f3*^+^ Late Aire 2 cells and *Pou2f3*^+^ thymic tuft cells in scATAC-seq (Fig. 3d and Supplementary Data 3). Furthermore, *Pou2f3* regulatory motif accessibility analysis of *Pou2f3*^+^ cells showed that thymic tuft cells were significantly different from mTEC II and Late Aire 2 cells (*P* < 0.01, Fig. 3e and Supplementary Data 3). In general, the analysis of *Pou2f3*^+^ mTEC subsets showed that both Late Aire 2 cells and thymic tuft cells had the ability to express *Pou2f3*, whether in chromatin accessibility or RNA expression, although its regulatory motif was only opened in thymic tuft cells. In both scRNA-seq and scATAC-seq, more than 30% of the tuft markers were regulated by *Pou2f3*, while less than 20% were regulated in other populations (Suppl. Fig. 26). Together, these data indicated that the expression of *Pou2f3* in mTECs alone is not sufficient to promote the expression of *Pou2f3*-regulated thymic tuft cell-related genes.

Furthermore, we found that the fragments of gene promoters and TSS enrichment in thymic tuft cells were higher than those in Late Aire 2 cells (Fig. 3f, g and Supplementary Data 3). To determine the specific role of increased chromatin accessibility in the development of Late Aire 2 cells to thymic tuft cells, we further analyzed the chromatin status of well-recognized important genes in thymic tuft cells. By analyzing the intersection of taste pathway-related genes (GO: 0050909, sensory perception of taste; KEGG: map04742, Taste transduction pathway), *Pou2f3*-regulated genes^26^ and genes highly expressed in thymic tuft cells, we identified that the overlapping genes included *Gnat3*, *Plcb2*, *Il25*, and *Trpm5* (Fig. 3h and Supplementary Data 4). In addition, we also selected genes that have been reported to be important in the thymus and small intestine tuft cells, including *Gnb3*, *Gng13*, *Tas1r1*, *Rgs13*, and *Alox5*. Consistent with the gene expression patterns, the chromatin of these genes was basically closed in Late Aire 2 mTECs and specifically opened in thymic tuft cells (Fig. 3i, j and Suppl. Fig. 27). Combined with the significant difference in gene accessibility shown in Fig. 3a, we speculate that in addition to the expression of *Pou2f3*, epigenetic regulation is essential for the development of thymic tuft cells.

### Deficiency of thymic tuft cell development in *Sirt6* cKO mice

**Fig. 4:**
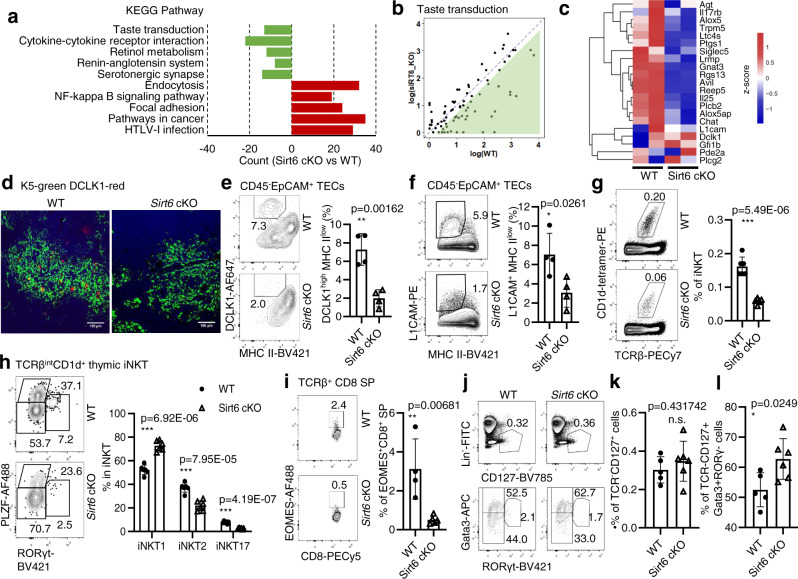
Sirt6 deletion affects the cell number and function of thymic tuft cells. **a** Downregulated genes or upregulated genes in *Sirt6*-deficient mTECs are enriched in KEGG pathways, and the top five pathways are ordered by *p* value. All pathways were selected under the standard of *p* < 0.05. **b** Scatter plot shows the difference in TPM values of taste transduction genes between WT and *Sirt6*-deficient mTECs. **c** Differential expression of thymic tuft cell-related genes between mTECs sorted from WT and *Sirt6* cKO mice. The color scale indicates the level of gene expression. **d** Representative images of frozen thymic sections from WT and *Sirt6* cKO mice (*n* = 4 per group). Green, KRT5. Red, DCLK1. Scale bars: 100 μm. **e** Representative flow cytometric profiles and frequency of thymic tuft cells (CD45^–^EpCAM^+^MHCII^low^DCLK1^high^) obtained from 4-week-old *Sirt6* cKO mice and littermates (*n* = 4 per group). **f** Representative flow cytometric profiles and frequency of L1CAM^+^ thymic tuft cells from 4-week-old *Sirt6* cKO mice and littermates (*n* = 4 per group). **g** Representative flow cytometric plots and frequency of TCRβ^int^CD1d^+^ thymic iNKT cells in 4-week-old *Sirt6* cKO mice and littermates (*n* = 6 per group). **h** Representative flow cytometric plots and frequencies of NKT1 (PLZF^–^RORγt^–^), NKT2 (PLZF^+^RORγt^–^), and NKT17 (PLZF^–^RORγt^+^) in 4-week-old *Sirt6* cKO mice and littermates (*n* = 6 per group). **i** Representative flow cytometric plots and frequency of EOMES^+^TCRβ^+^CD8 single-positive thymocytes in 4-week-old WT (*n* = 4) and *Sirt6* cKO (*n* = 5) mice. **j** Representative flow cytometric plots of Lin^–^TCR^–^CD127^+^ thymic ILCs (upper) and ILC subsets (lower) in *Sirt6* cKO mice and littermates. **k**, **l** Frequencies of Lin^–^TCR^–^CD127^+^ thymic ILCs (**k**) and Lin^–^TCR^–^CD127^+^Gata3^+^Rorγt^–^ ILC2s (**l**) of WT (*n* = 5) and Sirt6 cKO (*n* = 6) mice. *p < 0.05, **p < 0.01 and ***p < 0.001 compared with the identical WT control mice (Student’s *t*-test). The error bar represents one standard deviation.

Chromatin accessibility is regulated by numerous epigenetic regulators^27,28^. Previous studies have proven that SIRT6, as a key chromatin regulator, remodels chromatin by promoting chromatin relaxation, although it has diverse biological functions, such as genome stability, glucose metabolism, and tumor suppression, through multiple pathways^29–34^. We, therefore, bred Foxn1-Cre mice with *Sirt6*^flox/flox^ mice to obtain TEC-specific deletion of *Sirt6* mice as reported recently^35^. TEC-specific *Sirt6* deletion reduced the cell number of mTECs with an accelerated differentiation of CD80^-^Aire^-^ immature mTECs to CD80^+^Aire^-^ intermediate mature mTECs by promoting *Spib* expression^35^. However, we observed that the taste transduction signal pathway and taste-related genes were significantly downregulated in *Sirt6*-deficient mTECs, while endocytosis, NF-κB, and other pathways were enhanced, as detected by RNA-seq analysis (Fig. 4a, b and Supplementary Data 5). The known marker genes for thymic tuft cells compared with WT mTECs were also decreased after *Sirt6* deletion (Fig. 4c). By detecting the proportion of DCLK1^+^ thymic tuft cells, we found that *Sirt6* deletion blocked the development of thymic tuft cells, as determined by immunofluorescence staining and flow cytometry assays (*P* < 0.01, Fig. 4d, e and Supplementary Data 5). This conclusion was also confirmed by the decreased expression of L1CAM on thymic tuft cells in *Sirt6* cKO mice (Fig. 4f and Supplementary Data 5). Consistent with the decreased cell number of thymic tuft cells in *Pou2f3* knockout mice^4,5^, the functional deficiency of thymic tuft cells was evidenced by the abnormal development of NKT2 cells, EOMES^+^CD8^+^ single-positive thymocytes, and ILC2s in the thymus of *Sirt6* cKO mice (Fig. 4g–j and Supplementary Data 5). TCRβ^int^CD1d^+^ iNKT cells, especially RORγt^−^PLZF^+^ iNKT2 cells and RORγt^+^PLZF^−^ iNKT17 cells, decreased significantly in *Sirt6* cKO mice (*P* < 0.01, Fig. 4g, h and Supplementary Data 5). Meanwhile, EOMES^+^CD8^+^ single-positive cells were also markedly decreased after *Sirt6* deletion (*P* < 0.01, Fig. 4i and Supplementary Data 5). The proportion of ILC2s increased slightly after *Sirt6* ablation (Fig. 4j–l and Supplementary Data 5). These results clearly showed that *Sirt6* is essential for the development of thymic tuft cells.

### *Sirt6* deletion decreased the chromatin accessibility of *Pou2f3*-regulated genes in thymic tuft cells

**Fig. 5:**
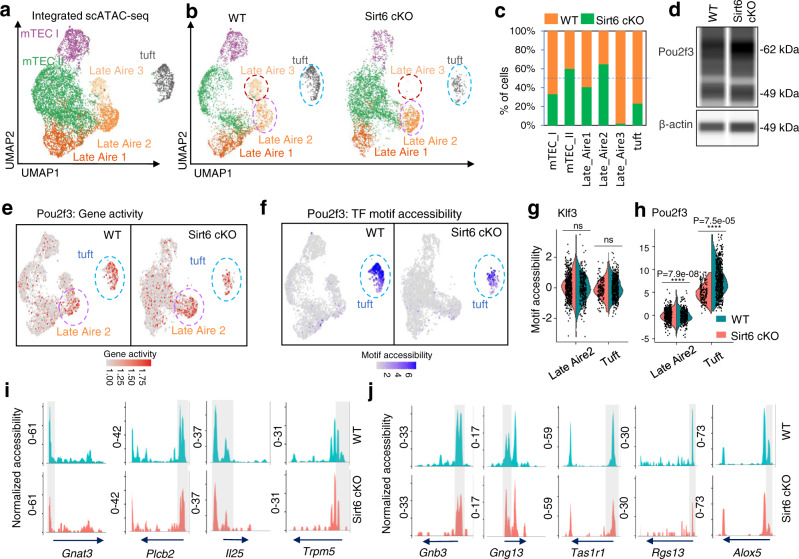
Chromatin accessibility of the genes regulated by Pou2f3 decreased after Sirt6 deletion. **a**, **b** scATAC-seq data of WT and *Sirt6*-deficient mTECs are coembedded into a single UMAP visualization (**a**) and are displayed separately (**b**). Cells are stained with the mTEC subset defined before. **c** Relative proportion of mTEC clusters between WT and *Sirt6* cKO mice. **d** Simple Western analysis of POU2F3 expression in sorted mTECs (CD45^−^EpCAM^+^UEA-1^+^Ly51^−^) from 4-week-old WT and *Sirt6* cKO mice. **e** UMAP visualization of the gene expression of *Pou2f3* in WT (left) and *Sirt6* cKO (right) scATAC-seq. The red scale in the UMAP plot indicates the level of *Pou2f3* gene activity in scATAC-seq **f** UMAP visualization of the motif accessibility score of *Pou2f3* in WT (left) and *Sirt6* cKO (right) scATAC-seq. The blue scale in the UMAP plot indicates the level of *Pou2f3* motif accessibility in scATAC-seq analyzed by chromVAR. **g**, **h** Motif accessibility scores of Klf3 (**g**) and Pou2f3 (**h**) in Late Aire 2 cells and thymic tuft cells of WT (Orange) and Sirt6 cKO mice (green) were compared in a violin plot. WT Late Aire 2 cells: *n* = 272, Sirt6 cKO Late Aire 2 cells: *n* = 500, WT thymic tuft cells: *n* = 501, Sirt6 cKO thymic tuft cells: *n* = 150. **i**, **j** Chromatin accessibility peaks of overlapping genes (**i**) and tuft-associated genes (**j**) mentioned in Fig. 3 in WT and *Sirt6*-deficient thymic tuft cells are shown in aggregated scATAC-seq tracks. The arrow indicates the length and direction of the genes detected by scATAC-seq. The statistics was determined using Wilcoxon signed-rank test. *p < 0.05, **p < 0.01, ***p < 0.005 and ****p < 0.001.

Sirt6 has been reported to regulate chromatin openness in many cells through its deacetylation^34,36^. By constructing *Sirt6* cKO mice, it was found that the cell number and function of thymic tuft cells in the thymus were significantly affected (Fig. 4). Therefore, scATAC-seq was also performed on *Sirt6* cKO mice to explore its possible effects on the developmental trajectory of thymic tuft cells. The sorting strategy and quality control rules are the same as those mentioned above (Suppl. Fig. 1, Method). Integrated scATAC-seq profiles were performed by unbiased clustering on all peaks of WT and *Sirt6* cKO mTECs using Signac, and the corresponding cell population annotation was predicted based on a gene activity matrix and coembedded analysis (Suppl. Fig. 28). Furthermore, we checked the modular score of subgroup-specific genes from scRNA-seq to determine the integrated scATAC-seq profile, which also proved that the correlation between the identified cell type and the predicted id was consistent (Suppl. Figs. 29,  30). Therefore, we obtained the distribution of well-established mTEC populations (mTEC I, mTEC II, Late Aire 1, Late Aire 2, Late Aire 3, and thymic tuft cells) based on integrated scATAC-seq data from WT and *Sirt6* cKO mice (Fig. 5a, b).

However, compared with WT control mice, *Sirt6* cKO mice showed increased mTEC II and Late Aire 2 subgroups and decreased mTEC I, Late Aire 3, and thymic tuft cells (Fig. 5b, c and Supplementary Data 6), which is nicely consistent with the cell phenotype alteration of mTECs analyzed by flow cytometry (Fig. 4 and ref. ^35^). The integrated scATAC-seq data did not reveal the developmental trajectory of thymic tuft cells, which was consistent with the WT scATAC-seq results mentioned above. In particular, Late Aire 2 of WT mice has the potential to differentiate into Late Aire 3, while Late Aire 2 of *Sirt6* cKO mice has the potential to develop into thymic tuft cells, but no Late Aire 3 (Suppl. Fig. 31). The increased Late Aire 2 proportion and decreased Late Aire 3 and thymic tuft cells in *Sirt6* cKO mice suggested that *Sirt6* deficiency may lead to the retardation of Late Aire 2 cells development to the subsequent stages (including Late Aire 3 cells and thymic tuft cells).

Since the transcription factor *Pou2f3* is a key factor controlling the development of thymic tuft cell development^4,5^, we detected the expression of *Pou2f3* in sorted WT and *Sirt6* cKO mTECs by the Simple Western assay. Unexpectedly, the expression of *Pou2f3* was not significantly affected after the *Sirt6* deletion (Fig. 5d and Suppl. Fig. 32). This observation was further confirmed by scATAC-seq analysis, which showed that the accessibility of *Pou2f3* in Late Aire 2 cells of WT and *Sirt6* cKO mice was identical (Fig. 5e). Thus, *Sirt6* deletion did not impact the accessibility and expression of *Pou2f3* in the thymus.

By comparing the peaks of *Klf3-* and *Pou2f3*-regulated motifs in Late Aire 2 cells and thymic tuft cells between WT and *Sirt6* cKO mice, we found that the peaks of *Pou2f3*-regulated motifs were reduced specifically in thymic tuft cells after *Sirt6* deletion, whereas *Klf3*-regulated motifs in Late Aire 2 cells and thymic tuft cells were unimpaired after *Sirt6* deletion (Fig. 5f–h, Suppl. Fig. 33, and Supplementary Data 6). Consistent with this result, the differentially accessible peaks of thymic tuft cells between WT and *Sirt6* cKO mice were analyzed. A subsequent motif enrichment analysis conclusively showed that *Pou2f3* was the most significant TF regulating the reduced accessible peak region after *Sirt6* deficiency (Suppl. Fig. 34). The top three TFs of motif enrichment, namely, KLF15, EGR1, and E2F6, in the open peak region after *Sirt6* deficiency were not necessary, and there was no significant increase in the motif activity score of those TFs (Suppl. Fig. 35). Therefore, *Sirt6* knockout did affect thymic tuft cells by affecting the accessibility of *Pou2f3* downstream genes. Furthermore, the chromatin accessibility of the identified key *Pou2f3*-regulated genes decreased in *Sirt6* cKO thymic tuft cells (Fig. 5i, j). Thus, *Sirt6* contributes to the development of thymic tuft cells by increasing the chromatin accessibility of *Pou2f3*-regulated motifs in the thymus.

### *Sirt6* deletion increased *Hdac9* expression to decrease the chromatin accessibility of *Pou2f3*-regulated genes

**Fig. 6:**
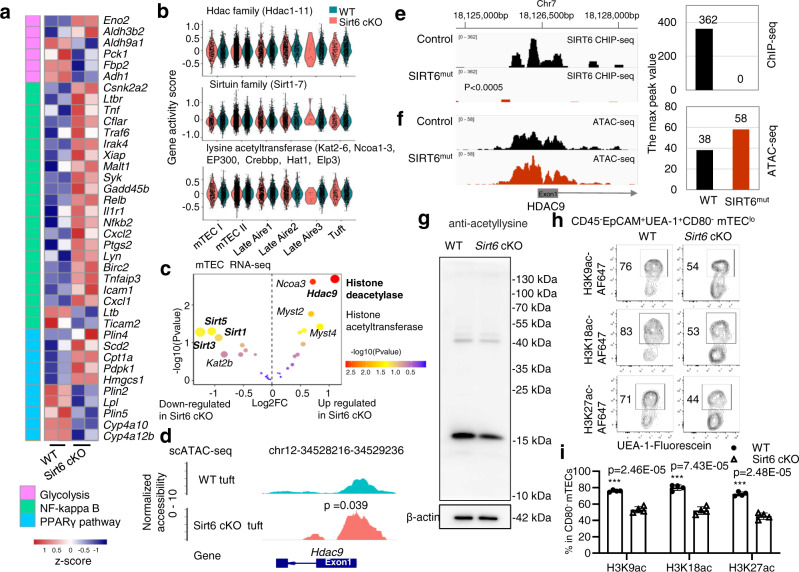
Decreased chromatin accessibility of Pou2f3-regulated genes was consistent with the decreased acetylation regulated by the SIRT6-HDAC9 axis. **a** Glycolysis-, NF-kappaB pathway-, and PPARγ pathway-associated genes were compared between WT and *Sirt6* cKO mTEC bulk RNA-seq by heatmap. The color scale indicates the level of gene expression. **b** Gene set module scores of the histone deacetylase gene family, sirtuin gene family, and histone lysine acetyltransferase gene family were compared between WT (green) and *Sirt6* cKO mouse (red) mTEC populations. **c** Differential expression of the histone deacetylase gene family, sirtuin gene family, and histone lysine acetyltransferase gene family was calculated by DEseq2 using WT and *Sirt6* cKO mTEC bulk RNA-seq. The x-axis represents Log2FC, and the y-axis represents −log10 (*P* value). The dot size is consistent with the |Log2FC| value. Histone deacetylase-related genes are labeled in bold, and histone acetyltransferase-related genes are not labeled in bold. **d** Chromatin accessibility peaks around the promoter of the *Hdac9* gene between WT and *Sirt6* cKO thymic tuft cells are shown in aggregated scATAC-seq tracks. The arrow indicates the length and direction of the peak around the promoter region of *Hdac9* detected by scATAC-seq. **e** Capture of the UCSC (GRCh37/hg19) genome browser showing the human *HDAC9* locus in ChIP-seq. **f** Capture of the UCSC (GRCh37/hg19) genome browser showing the human *HDAC9* locus in ATAC-seq. **g** Western blot analysis of protein acetylation in mTECs (CD45^−^CD326^+^UEA-1^+^Ly51^−^) isolated from wild-type or *Sirt6* cKO mice. **h** Representative flow cytometric profiles of H3K9ac, H3K18ac, and H3K27ac expressed in CD80^−^ mTECs obtained from 4-week-old *Sirt6* cKO mice and littermates. **i** Frequencies of H3K9ac, H3K18ac, and H3K27ac expression in CD80^−^ mTECs of 4-week-old *Sirt6* cKO mice and littermates (*n* = 4 per group). ***p < 0.001 compared with the identical WT control mice (Student’s *t*-test). The error bar represents one standard deviation.

SIRT6 plays a pivotal role in regulating some fundamental cellular processes, such as cellular metabolism, DNA repair, gene expression, mitochondrial biology, and telemetric maintenance^37,38^. Deacetylation of specific sites on histone H3 (H3K9, H3K56, and H3K18) by SIRT6 can promote chromatin compaction and repress transcription of target genes^39–44^. SIRT6 predominantly represses the expression of various genes, such as glycolytic genes, Myc-target ribosomal protein genes, lipid metabolism genes, and NF-κB-dependent inflammatory genes^29–33,45,46^. In parallel with these studies, *Sirt6*-deficient mTECs expressed higher glycolysis-, NF-κB pathway-, and PPARγ pathway-related genes, as detected by bulk RNA-seq assays (Fig. 6a). However, the decreased *Pou2f3* target gene accessibility caused by *Sirt6* deletion is somehow contradictory to the general conception of the biofunction of *Sirt6*. Therefore, we observed increased gene expression profiles related to histone acetylation, including the families of histone deacetylase (HDAC), SIRTUIN, and histone lysine acetyltransferase (KAT), in *Sirt6*-deleted thymic tuft cells. The scATAC-seq assays showed that although the overall gene accessibility of the SIRTUIN, HDAC, and KAT families was unchanged in *Sirt6*-deleted thymic tuft cells (Fig. 6b and Supplementary Data 7), some genes, including the histone deacetylase gene (*Hdac9*) and histone acetyltransferase genes (*Ncoa3*, *Myst2*, and *Myst4*), were significantly upregulated in mTECs after *Sirt6* deletion (Fig. 6c), while some histone deacetylase genes (*Sirt5*, *Sirt3*, and *Sirt1*) were significantly downregulated (Fig. 6c). In particular, *Hdac*9 was the only deacetylase gene with an accessible promoter region after deletion of *Sirt6* in thymic tuft cells (Suppl. Fig. 36a). In addition, the peak of the promoter region of *Hdac9* was significantly accessible in thymic tuft cells (Suppl. Fig. 36b). The chromatin accessibility of the *Hdac9* promoter was also significantly increased in *Sirt6*-deleted thymic tuft cells (Fig. 6d), indicating that *Sirt6* may promote chromatin accessibility of thymic tuft cells by inhibiting *Hdac9*.

Additionally, *SIRT6* was found specifically bind the *HDAC9* promoter region in SKMel-239 cell lines (Fig. 6e and Suppl. Fig. 37). This observation was further confirmed by the *HDAC9* promoter had a tendency to increase chromatin accessibility in the SKMel-239 cell lines with a homozygous mutant *SIRT6*^36^, although the significance cannot be calculated due to lack of replicates (Fig. 6f and Suppl. Fig. 37). Consistent with the enhanced histone deacetylase *Hdac9* expression, acetylation in *Sirt6*-deficient mTECs, including H3K9ac, H3K18ac, and H3K27ac, was significantly decreased, as determined by Western blot and flow cytometry (*P* < 0.001, Fig. 6g–i, Suppl. Fig. 38, and Supplementary Data 7). Thus, deletion of histone deacetylase SIRT6 indirectly decreased the chromatin accessibility of *Pou2f3*-regulated motifs, likely by increasing histone deacetylase gene *Hdac9* expression, and finally impaired *Pou2f3*-mediated thymic tuft cell development.

## Discussion

We presented the scATAC-seq data of mouse mTECs showing the overall profiles from chromatin accessibility profiling of mTECs in all development stages. We found that the Late Aire-expressing mTEC III subgroup might include the Late Aire 1–3 subpopulations with distinctive gene accessibility profiles (Supplementary Data 8). Late Aire 1 mTECs likely differentiate into Late Aire 2 mTECs, which then differentiate into either Late Aire 3 cells or thymic tuft cells. Unexpectedly, the key transcription factor *Pou2f3* was also widely expressed in pre- and Late Aire-expressing mTECs, but its regulated target genes were specifically expressed in thymic tuft cells, indicating critical roles of epigenetic modulation in *Pou2f3*-mediated thymic tuft cell development. Furthermore, *Sirt6* deletion did not impact *Pou2f3* expression in mTECs but significantly diminished the presence of thymic tuft cells by regulating chromatin accessibility of *Pou2f3*-regulated motifs. More importantly, Late Aire 3 cells almost completely disappeared after *Sirt6* knockout, indicating that epigenetic regulation is indispensable in the development of Late Aire 2 cells to thymic tuft cells and Late Aire 3 cells.

Based on the scATAC-seq data of mouse mTECs, we confirmed the rich diversity of mTECs and could roughly divide mTECs into mTEC I-III and thymic tuft cells, as recently reported with scRNA-seq data^5^, indicating that promoter and distal region accessibility is linked to the expression of genes contributing to mTEC subgroup identity. Previous reports have shown that mTECs also contain a TAC-TEC cluster^47^, although our scATAC-seq data showed that the chromatin accessibility of the TAC-TEC characteristic gene set (cell cycle-related genes) was similar to that in other mTEC clusters. This finding is consistent with another report that chromatin accessibility of cell cycle-related genes has a limited contribution to gene expression in shared single-cell profiling^48^. Therefore, we did not conduct further chromatin accessibility analysis for the TAC-TEC cluster.

Among Late Aire-expressing mTEC III cells, we found heterogeneity and identified Late Aire 1-3 subgroups with distinct transcription factors in each subgroup, such as TCF7L2 in Late Aire 1 cells, KLF3 in Late Aire 2 cells, and FOXA3 in Late Aire 3 cells. mTEC III, as a terminally differentiated mTEC cluster, downregulates the expression of MHC II and Aire^49^ and highly expresses KRT1, KRT10, involucrin, desmogleins, SPINK5, Asprv1, cystatin, LY6D, and Pigr^1,5–7,49–52^. Late Aire 1 cells still expressed *H2-DMb2*, *H2-Oa*, *H2-M2*, and *Aire* (Suppl. Fig. 8), suggesting that Late Aire 1 has a strong developmental correlation with mTEC II, which is speculated to be the early stage of mTEC terminal differentiation. Late Aire 2 cells specifically expressed *Spink5* and *Ly6d*, suggesting that Late Aire 2 is more consistent with the known terminally differentiated mTECs^5,53^. Late Aire 3 cells with high expression of *Ascl1* may be similar to the reported Ascl1^+^ mTEC^lo^ ^8^; however, the specific function of Late Aire 3 needs to be further studied. Using scATAC-seq to reconstruct the cellular developmental trajectories of these mTECs in an unbiased manner, we found that Late Aire 1 cells likely differentiated to Late Aire 2 cells and finally to Late Aire 3 cells. On the other hand, some Late Aire 2 cells also differentiated into thymic tuft cells. Thus, Late Aire 2 mTECs may represent a crossroad for the differentiation into either Late Aire 3 cells or thymic tuft cells. Lineage-tracing studies by two independent research teams showed that more than half of thymic tuft cells arise from mature mTEC^high^ cells^4,5^, with the remaining half of the thymic tuft cells not appearing to pass through the AIRE-expressing mTEC stage. Specifically, diphtheria toxin treatment of mice, in which diphtheria toxin receptor expression is driven by the Aire promoter, resulted in only a partial deletion or even no significant impact of thymic tuft cells with no marked alterations in the gene expression profile of thymic tuft cells^4,5^. Thus, this thymic tuft cell differentiation pathway identified in the present study requires to be verified in further experiments.

Of note, Late Aire 2 cells exhibited similar accessibility levels of the key transcription factor of thymic tuft cells, namely, *Pou2f3*, as determined by transcriptional expression and open chromatin features. However, the *Pou2f3*-regulated genes were not expressed in Late Aire 2 cells but were specifically expressed in thymic tuft cells, and the loci of *Pou2f3*-target genes were inaccessible in Late Aire 2 cells, likely reflecting a requisite gain of accessible chromatin at that gene’s locus. These data strongly highlight the importance of chromatin accessibility and epigenetic modulation in *Pou2f3*-mediated thymic tuft cell differentiation; that is, *Pou2f3* is essential but not sufficient for thymic tuft cell differentiation.

SIRT6, as a key chromatin regulator, predominantly represses the expression of various genes, such as glycolytic genes, Myc-target ribosomal protein genes, lipid metabolism genes, and NF-κB-dependent genes, to regulate some fundamental cellular processes, such as cellular metabolism, DNA repair, gene expression, mitochondrial biology, and telemetric maintenance^29–33,45,46^. The expression of these genes in mTECs and thymic tuft cells was upregulated after *Sirt6* deletion. However, the expression and locus openness of *Pou2f3*-regulated genes were obviously decreased, which blocked the differentiation of *Pou2f3*-mediated thymic tuft cells. Unexpectedly, histone acetylation, including H3K9ac, H3K18ac, and H3K27ac, was decreased in *Sirt6*-deficient CD80^−^ mTECs, which is inconsistent with the histone deacetylase function of SIRT6. We thus screened the expression of histone deacetylase and histone acetyltransferase and identified that histone deacetylase *Hdac9* expression might be directly regulated by SIRT6 in thymic tuft cell differentiation. Enhanced histone deacetylase *Hdac9* expression by *Sirt6* deletion subsequently decreased the chromatin accessibility of *Pou2f3*-regulated motifs to block *Pou2f3*-mediated thymic tuft cell development. Therefore, epigenetic regulators are essential for the development of thymic tuft cells.

By describing the chromatin landscape of mTECs, we identified previously unknown mTEC subpopulations and explored developmental trajectories and epigenetic regulation from mTEC III to thymic tuft cells. The difference between chromatin accessibility and gene expression contributes to an in-depth understanding of the development of mTECs.

## Methods

### Mice

Consistent with our previous reports^35^, the Foxn1-Cre mouse strain (a gift from Dr. Yu Zhang)^54,55^ was crossed with *Sirt6*^loxp/loxp^ mouse strain (a gift from Dr. Zhenyu Ju)^56,57^ to generate the Foxn1-Cre *Sirt6*^loxp/loxp^ mice and Foxn1-Cre negative littermate controls. Mice were maintained in the Institute of Zoology, Chinese Academy of Sciences (IOZ, CAS) specific pathogen-free conditions and approved by the Animal Ethics Committee of Institute of Zoology, Beijing, China.

### Thymic stromal cell isolation

Thymi from 4-week-old wild-type and *Sirt6* cKO mice were placed into 6 cm dishes, chopped into small pieces with surgical scissors, and suspended in DMEM (HyClone Laboratories, SH30022.01B) containing 2% fetal bovine serum (FBS; Gibco, 16000-044). After removing most thymocytes from the supernatant, the remaining thymus fragments were digested with a solution containing 20 mg/ml collagenase/dispase (Sigma-Aldrich, 11097113001) and 20 U/ml DNase I (Sigma-Aldrich, d5025) for 40 min in a water bath at 37 ^o^C^58^. When the small pieces disappeared, the cell suspension was filtered through a 200-mesh filter to obtain a single-cell suspension.

### scATAC-seq library generation and sequence alignment

The cell suspension mentioned above was incubated with CD45 microbeads (Miltenyi, mouse, 130-052-301) in the dark for 15 min on ice, and the CD45-negative cells were enriched by using a LS column (Miltenyi, 130-042-401). After incubation with antibodies, wild-type samples (150000 CD45^-^CD326^+^ TECs) and *Sirt6* cKO samples (100000 CD45^−^CD326^+^ TECs plus 8400 CD45^-^CD326^+^L1CAM^+^MHC II^low^ thymic tuft cells) were sorted by BD FACSAria™ Fusion Flow Cytometers (BD Biosciences, USA) for the 10× Genomics scATAC-seq analysis.

The nuclei were isolated and washed according to the method in the 10X Genomics Chromium Single-Cell ATAC Reagent Kit user guide (CG000169). Then, the nuclei were resuspended in chilled diluted nuclei buffer (10x Genomics; 2000153). The nuclei in bulk samples were partitioned into nanoliter-scale gel beads-in-emulsions (GEMs), a pool of ~750,000 10x barcodes was sampled to separately and uniquely index the transposed DNA of each individual nucleus, and libraries were generated (performed by CapitalBio Technology, Beijing). The libraries were sequenced using an Illumina NovaSeq sequencer with a sequencing depth of at least 25 k read pairs per nucleus using a paired-end 50 bp (PE50) reading strategy.

Raw sequencing data were converted to FASTQ format using Cell Ranger ATAC mkfastq. Alignment of scATAC-seq analyses was completed utilizing the 10x Genomics Cell Ranger ATAC pipeline (version 1.2.0). Each library was aligned to an indexed mm10 genome using Cell Ranger ATAC Count.

### scRNA-seq data process and cell type clustering

The gene expression matrix data of TEC scRNA-seq were obtained from GSE137699^17^. We merged 2-week and 10-week wild-type control samples for a Seurat (4.0.1) analysis. The scRNA-seq project was constructed by the Seurat (4.0.1) R package with at least 200 genes per cell and at least three cells. Cells were filtered based on the number of gene features per cell, the number of reads per gene, the percent of ribosome-associated genes, and the percent of mitochondrial-associated genes, the value of which was restricted to 5 to 95%. After filtering, 19,014 features across 1875 cells remained for further analysis. Normalization and scale strategies were performed according to a previous method^17^ as follows. Quality control of scRNA-seq was based on a 5–95% threshold of the count of reads, a number of the feature genes, and the percent of mitochondria-related genes. The cutoff strategy of WT TEC scRNA-seq project was using the options “2274 < nCount_RNA < 3699, 1361 < nFeature_RNA < 6587, 0.007 < percent.mito < 0.014”. The scale and clustering strategy was using the options “ScaleData: scale.factor = 1000; FindVariableFeatures: mean.function = ExpMean, dispersion.function = LogVMR, x.low.cutoff = 0.0125, x.high.cutoff = 3, y.cutoff = 0.5; FindClusters: resolution = 0.6, random.seed = 0; RunUMAP: dims = 1:13”. For our analysis, we filtered *Ly75*^+^*Psmb11*^+^ cTECs for consistency with scATAC-seq data. The clustree (0.4.4) package was used to visualize the allocation of cells between clusters at different resolutions. The marker genes of each cluster were generated by Seurat’s default FindAllMarkers() function on the threshold of p_val_adj < 0.05 and avg_log2FC > 0.5. The initial cell population was defined by mTEC I (*Itgb4*, and *Itga6*), mTEC II (*Aire* and *Cd80*), mTEC III (*Ly6d* and *Pigr*), and tuft (*L1cam* and *Gnat3*) cells. The gene set module scores of the mTEC I, mTEC II, mTEC III, and thymic tuft cells were determined by the AddModuleScore() function.

### Data processing and clustering analysis

The aggregated peak-by-cell data matrix was read into R (R version 4.0.3) and processed using Seurat (4.0.1)^59^ and Signac (1.1.1). The chromatin assay was constructed by CreateChromatinAssay and transferred to the Seurat object by CreateSeuratObject.

Blacklist regions indicate abnormal or high-signal areas in either of the next-generation sequencing experiments, and the exclusion of these regions can provide quality assurance for further analysis of functional genomic data. The nucleosome banding pattern, defined as a histogram of DNA fragment sizes (determined from the paired-end sequencing reads), should exhibit a strong nucleosome banding pattern corresponding to the length of DNA wrapped around a single nucleosome. Quality control of scATAC-seq was based on a 5–95% threshold of the fraction of fragments in peaks (pct_reads_in_peaks), the total number of fragments in the peak region (peak_region_fragments), the ratio of reads in the blacklist region (blacklist_ratio), the TSS enrichment score (TSS.enrichment) and the nucleosome binding pattern (nucleosome_signal). The cutoff strategy of WT TEC scATAC-seq project was using the options “60.01 < pct_reads_in_peaks < 88.95, 883 < peak_region_fragments < 17873, 0 < blacklist_ratio < 0.02, 2.30 < TSS.enrichment < 12.96, 0.03 < nucleosome_signal < 2.87” and the *Sirt6* cKO TEC scATAC-seq project was using the options “60 < pct_reads_in_peaks < 79.9, 715 < peak_region_fragments < 10427, 0 < blacklist_ratio < 0.014, 2.81 < TSS.enrichment < 8.41, 0.02 < nucleosome_signal < 0.46”. Latent semantic indexing (LSI) was used to calculate the variable features, and the first ten components were used to generate a uniform manifold approximation and projection (UMAP) dimensionality reduction. To define the subsets of WT TECs, a shared-nearest-neighbor graph was generated from the 2:10 LSI components and used to cluster the cells via Seurat’s Louvain algorithm.

Notably, 50% of the *Sirt6* cKO thymic tuft cells were randomly excluded based on the number of externally added cells to ensure that the cell proportion of the data was consistent with the true value in vivo. After that, WT TEC and *Sirt6* cKO filtered TEC scATAC-seq data were merged into a combined object for further analysis.

### Cell population and marker identification

The gene activity matrix was calculated for the filtered peak matrix by the Cicero analysis package (1.7.1)^60^ and monocle 3 alpha package (2.99.3)^61^, and it was added to the Seurat object based on RNA assays. Marker genes for peak-based clustering were generated using the FindAllMarkers() function on the gene activity matrix based on the threshold of p_val_adj <0.05 and avg_log2FC > 0.5. The shared characteristic genes between scATAC-seq and scRNA-seq of mTEC I, mTEC II, mTEC III and thymic tuft cells calculated by scRNA-seq mentioned before were added to the peak-by-cell data matrix through the AddModuleScore() function. The populations of mTEC I (*Itgb4* and *Itga6*), mTEC II (*Aire* and *Cd80*), mTEC III (*Ly6d* and *Pigr*), and thymic tuft cells (*L1cam* and *Gnat3*) in WT scATAC-seq and combined scATAC-seq were all established by marker genes and the scRNA-seq clustering gene set module score.

### Repopulation of the Late Aire subset

Late Aire repopulation was performed by WT scATAC-seq according to the population heterogeneity and unique gene expression profiles. The Late Aire 1, Late Aire 2, and Late Aire 3 marker genes calculated by FindMarkers() according to the threshold of p_val_adj <0.05 and avg_log2FC > 0.5 were derived from the gene activity matrix of scATAC-seq. The shared characteristic genes between WT scATAC-seq and scRNA-seq of Late Aire 1, Late Aire 2, and Late Aire 3 calculated by scATAC-seq were added to the scRNA-seq gene expression matrix through the AddModuleScore() function. The three subsets of mTEC III cells of the combined data were generated by the same method as mentioned above.

### TF motif analysis

A motif enrichment analysis was performed using Signac (1.1.1) and ChromVAR (1.10.0)^25^. We created the motif class to store all the required information, including a list of position weight matrices (PWMs) or position frequency matrices (PFMs) and a motif occurrence matrix derived from the JASPAR2020 (species: 9606) database using the AddMotifs() function. Open chromatin peaks of each subset were defined by the FindAllMarkers() function on the peak assay of scATAC-seq based on the threshold of avg_log2FC > 0.5. The open chromatin peaks of each subset were used to perform DNA sequence motif analysis by FindMotifs(). Differentially accessible peaks between WT and *Sirt6* cKO cells were calculated by FindMarkers(), and then FindMotifs() analysis was performed. All of the motif activity scores were run by chromVAR and visualized by FeaturePlot() and VlnPlot().

### Pseudotemporal analyses

For the WT scATAC-seq analysis, subsets of cells pertaining to mTEC III (including Late Aire 1, Late Aire 2, and Late Aire 3) and thymic tuft cells were extracted for pseudotime analysis by Monocle3 (0.2.1). Using the UMAP cell positions of the extracted subset from the scATAC-seq analysis, we next employed monocle to learn a graph trajectory through this space. The start of pseudotime was chosen as the branch node that started within the Late Aire 1 subset.

### Pou2f3 target gene selection

The Pou2f3 ChIP-seq data used in this article were downloaded from small intestine tissue (GSE116510). Reads were trimmed for Illumina adapter sequences using trim_galore (version 0.5.0) and aligned to mm10 using hisat2 (version 2.1.0). Pou2f3 significant peaks were identified using MACS2 (version 2.1.1) using the parameters--nomodel--shift-50--extsize 100, and matching input control was used to call peaks. ChIPpeakAnno (3.28.0) was used to annotate the peaks found by MACS2, matching the upstream and downstream 2 kb regions of genes in the mm10 genome to obtain 2152 predicted genes, which are provided in Supplementary Data 1.

### Epigenetic sequencing data analysis of the human SIRT6 knockout cell line

The interaction between SIRT6 and HDAC9 was analyzed by ChIP-seq (SIRT6) and ATAC-seq of WT and SIRT6 KO SKMel-239 cells derived from GSE102813^36^. The Bigwig file (.bw) containing the axis information and related scores of the peak region can be downloaded from the Gene Expression Omnibus (GEO) database. The visualization of peak regions was performed by Integrative Genomics Viewer^62^ software.

### Bulk RNA-seq analysis

Bulk RNA-seq of WT and *Sirt6* cKO mTECs was derived from our previously published studies, and the data are available in GSE166840^35^. The differentially expressed genes were calculated by the DEseq2 (1.28.1) package according to the threshold of adjusted *p* value <0.05 and |Log2FC | > 2. The KEGG analysis of the differentially expressed genes was performed on the KOBAS 3.0 website.

### ChIP-seq analysis

Reads were trimmed for Illumina adapter sequences using trim_galore (v 0.5.0) and aligned to the mm10 using hisat2 (v 2.1.0). Pou2f3 significant peaks were identified using MACS2 (v 2.1.1) with parameters--nomodel--shift-50--extsize 100 and matching Input control was used to call peaks. ChIPpeakAnno (v 3.28.0) was used to annotate the peaks found by MACS2, matching the upstream and downstream 2 Kb regions of genes in EnsDb.Mmusculus.v79 genome to obtain 2152 predicted genes.

### Flow cytometric analysis and antibodies

After the Fc receptor was blocked by 2.4G2, the cells were incubated with the designated antibody for 30 min at 4 °C^63^. For different intracellular staining, according to the instructions, fixation buffer (eBioscience, 00-5123-43 and 00-5223-56) and permeabilization buffer (eBioscience, 00-8333-56) were used for nuclear antibody staining, and BD Cytofix/Cytoperm™ Fixation and Permeabilization Solution (BD Biosciences, 554722) was used for cytoplasmic antibody staining. A BD LSRFortessa X-20 flow cytometer (BD Biosciences, USA) was used for the flow cytometry analysis.

The following antibodies were used: Fixable Viability Dye eFluor™ 506 (eBioscience, 65-0866-18, 1:400), CD45-BUV395 (BD Biosciences, clone 30-F11, 564279, 1:400), CD326-PE/Cy7 (Biolegend, clone G8.8, 188216, 1:800), UEA-I (Vector Laboratories, FL-1061, 1:300), Ly51-AF647 (Biolegend, clone 6C3, 108312, 1:300 dilution), Ly51-BV786 (BD Biosciences, clone BP-1, 740882, 1:300 dilution), I-A/I-E-BV421 (Biolegend, clone M5/114.15.2, 107632, 1:400 dilution), CD80-PE (Biolegend, clone 16-10A1, 104708, 1:2000 dilution), CD80-BV650 (Biolegend, clone 16-10A1, 104732, 1:300 dilution), L1CAM-PE (R&D, clone 555, FAB5674P, 1:20 dilution), rabbit anti-DCLK1 (Abcam, ab31704, 1:15000 dilution), TCR-β-PE-Cy7 (Biolegend, clone H57-597, 109222, 1:500 dilution), mouse CD1d loaded R-PE label (Proimmune, E001-2A-G, 1:600 dilution), RORγt-BV421 (BD Biosciences, clone Q31-378, 562894, 1:200 dilution), PLZF-AF488 (eBioscience, clone Mags.21F7, 53-9320-80, 1:200 dilution), CD4-PE (Biolegend, clone GK1.5, 100408, 1:1600 dilution), CD8a-PE/Cy5 (Biolegend, clone 53-6.7, 100710, 1:1600 dilution), EOMES-AF488 (eBioscience, clone Dan11mag, 53-4875-80, 1:200 dilution), CD127-BV785 (Biolegend, clone A7R34, 135037, 1:200 dilution), GATA3-APC (Biolegend, clone 16E10A23, 653805, 1:100 dilution), T-bet-PE (eBioscience, clone 4B10, 12-5825-82, 1:200 dilution); Lin^-^-FITC: CD4-FITC (Biolegend, clone GK1.5, 100405, 1:1000 dilution); CD8-FITC (BD Biosciences, clone 53-6.7, 553031, 1:400 dilution); CD19-FITC (Biolegend, clone 1D3/CD19, 153404, 1:200 dilution); CD11c-AF488 (eBioscience, clone N418, 53-0114-82, 1:400 dilution); CD11b-FITC (eBioscience, clone M1/70, 11-0112-82, 1:500 dilution); F4/80-FITC (eBioscience, clone BM8, 11-4801-82, 1:500 dilution); TCRγδ-FITC (BD Biosciences, clone GL3, 553177, 1:200 dilution); NK1.1-FITC (eBioscience, clone PK136, 11-5941-81, 1:400 dilution); TER-119-FITC (Biolegend, clone TER-119, 116206, 1:400 dilution); Alexa Fluor 647 AffiniPure donkey anti-Rabbit IgG (H + L) (Jackson ImmunoResearch Laboratories, 703-545-155, 1:600 dilution). Acetyl-Histone H3 (Lys9) (C5B11) Rabbit mAb 9649 (1:1000 dilution), Acetyl-Histone H3 (Lys18) (D8Z5H) Rabbit mAb 13998 (1:1000 dilution) and Acetyl-Histone H3 (Lys27) (D5E4) XP^®^ Rabbit mAb 8173 (1:200 dilution), all from Acetyl-Histone H3 Antibody Sampler Kit (Cell Signaling Technology, 9927).

### Immunofluorescence staining

Frozen section samples of thymus tissue from 4-week-old WT and *Sirt6* cKO mice were prepared according to previous methods^4,5,35^. Frozen slice samples (25 μm in thickness) were fixed with 4% polyoxymethylene (Solarbio, P1110) for 20 min and blocked in PBS containing 1% BSA and 0.3% Triton X-100 (Sigma–Aldrich) for 1 h^64^. Then, the sliced samples were stained with primary and secondary antibodies for 1 h. DAPI staining was performed for 10 min after the above steps. The following antibodies were used for staining: chicken anti-KRT5 (Biolegend, 905901; clone Poly9059) diluted by 1:300 and rabbit anti-DCLK1 (Abcam, ab31704) diluted by 1:250. Alexa Fluor 488-conjugated donkey anti-Chicken IgG (H + L) (Jackson ImmunoResearch Laboratories, 703-545-155) diluted by 1:300, Alexa Fluor 594-conjugated donkey anti-rabbit IgG (H + L) (Jackson ImmunoResearch Laboratories, 711-586-152) diluted by 1:300. All antibodies were diluted in PBS (0.5% BSA). Laser scanning confocal microscope (Zeiss LSM710, Oberkochen, Germany) were used to obtain images.

### Simple Western

Referring to a previous method^65^, mTECs (CD45^−^CD326^+^UEA-1^+^Ly51^−^) isolated from 4-week-old WT and *Sirt6* cKO mice were lysed with RIPA lysis buffer (Beyotime, P0013B) supplemented with 1 mM PMSF (Beyotime, ST506). According to the manufacturer’s instructions, a 12–230 kDa separation module (ProteinSimple, SM-W002-1) was used to detect the expression of POU2F3. The lysate was mixed with 5× Fluorescent master mix at a ratio of 4:1 and heated with a biotinylated ladder at 95 ^o^C for 5 min. The primary antibody, anti-POU2F3 (Prosci, 7795), was diluted with Antibody Diluent II at 1:40, while the anti-rabbit secondary HRP antibody (042-206; ProteinSimple) was used directly. Lumino-S and peroxide were mixed and used as substrates. The above reagents were added to the plate according to the instructions and centrifuged at 2500 rpm for 5 min at room temperature. Finally, WES (ProteinSimple, USA) was used to detect the protein.

### Western blot assay

Consistent with a previous description^35^, mTECs (CD45^−^CD326^+^UEA-1^+^Ly51^−^) sorted from 4-week-old WT and *Sirt6* cKO mice were lysed with RIPA lysis buffer to detect protein acetylation. Protein was separated by 10% SDS–PAGE and transferred to PVDF membranes (Merck Millipore, IPFL00010). After blocking with 5% skim milk powder (Oxoid, LP0031) at room temperature for 60–90 min, the primary antibody was incubated overnight at 4 °C. The primary antibodies were anti-acetyllysine mouse mAb (PTM BIOLABS, clone Kac-01, PTM0101) diluted at 1:1000 and anti-actin (Sigma–Aldrich, A5441) diluted at 1:40000. Then, the corresponding secondary antibody was added at room temperature for 45 min. Chemiluminescence (Merck Millipore, WBKLS0500) was used to detect protein expression.

### Statistics and reproducibility

The statistical significance of the gene expression and activity between mTEC populations visualized by boxplot and violin plot was determined using Wilcoxon signed-rank test. Boxplot and violin plots were visualized by ggpubr (v 0.4.0) and R (v4.0.3). The statistical significance of the difference in cell ratio of WT and Sirt6 cKO detected by flow cytometric was determined by Student’s *t*-test. The bar graphs were visualized using GraphPad Prism (v8) and the replication were in 4–6 independent experiments, as indicated in the figure legends. FDR-adjusted *P* values and fold changes of differential expressed genes between WT and Sirt6 cKO were derived from the DESeq2 package (v1.28.1). *P* values for KEGG pathway enrichment were derived from clusterProfiler package (v 3.16.1).

### Reporting Summary

Further information on research design is available in the Nature Research Reporting Summary linked to this article.

## Supplementary information

Supplementary Information

Description of Additional Supplementary Files

Supplementary data 1

Supplementary data 2

Supplementary data 3

Supplementary data 4

Supplementary data 5

Supplementary data 6

Supplementary data 7

Supplementary data 8

Reporting Summary

## Data Availability

The gene expression matrix data of TEC scRNA-seq was obtained from GSE137699. The analysis of the interaction between SIRT6 and HDAC9 were performed by the ChIP-seq (SIRT6, H3K9ac, H3K27ac, and H3K56ac) and ATAC-seq of WT and SIRT6 mutant SKMel-239 cell line derived from GSE102813. The bulk RNA-seq of WT and Sirt6 cKO mTEC were derived from our recently published studies^35^, and the data was available on GSE166840. The genes regulated by POU2F3 were calculated by the ChIP-seq (POU2F3) of intestinal tuft derived from GSE116510. The raw data of scATAC-seq sequencing used in this article have been deposited at the National Genomics Data Center BioProject under number: PRJCA006664. Uncropped Western blots gel for Figs. 5c, 6g are presented in Suppl. Figs. 32, 38, respectively.
