# Supplementary material for: Sirt6-mediated epigenetic modification of DNA accessibility is essential for Pou2f3-induced thymic tuft cell development
Source: Commun Biol. 2022 Jun 6;5:544. doi: 10.1038/s42003-022-03484-9 (PMC9170729; doi:10.1038/s42003-022-03484-9)
Supplement: Supplementary file 1 — Supplementary Information [file 42003_2022_3484_MOESM1_ESM.pdf]

***Sirt6*-mediated epigenetic modification of DNA accessibility is essential for *Pou2f3*-induced thymic tuft cell development**

Qian Zhang[1,2,#], Jiayu Zhang[1,2,#], Tong Lei[1,2,#], Zhanfeng Liang[1,2], Xue Dong[1,2], Liguang Sun[3,*], Yong Zhao[1,2,4,*]

[1] State Key Laboratory of Membrane Biology, Institute of Zoology, Chinese Academy of Sciences, Beijing, China; [2] University of Chinese Academy of Sciences, Beijing, China; [3] National-local Joint Engineering Laboratory of Animal Models for Human Diseases, Institute of Translational Medicine, the First Hospital, Jilin University, Changchun, Jilin, China. [4] Institute for Stem Cell and Regeneration, Chinese Academy of Sciences, Beijing, China.

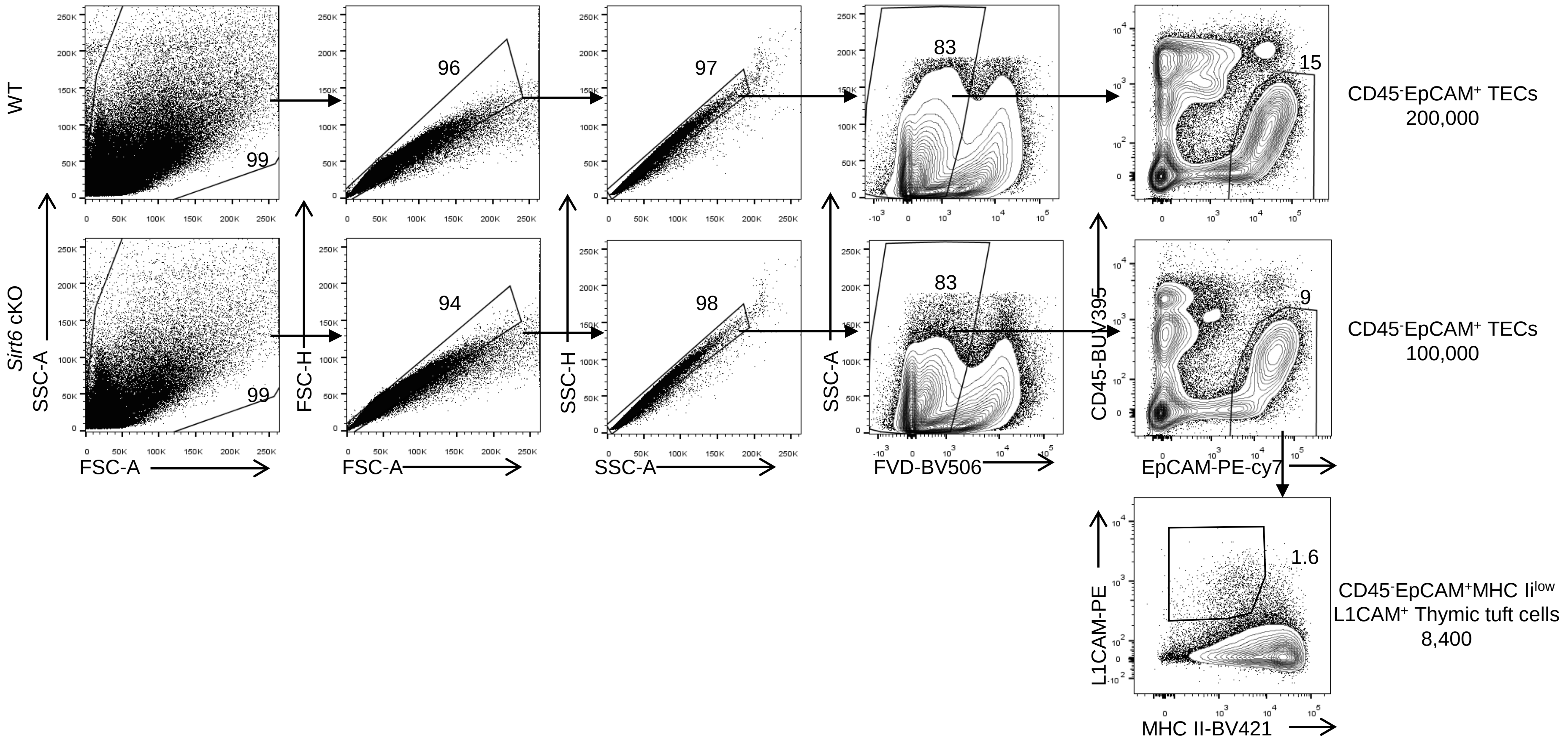

Supplementary Figure 1. Cell sorting strategy of WT and Sirt6 cKO mTEC.

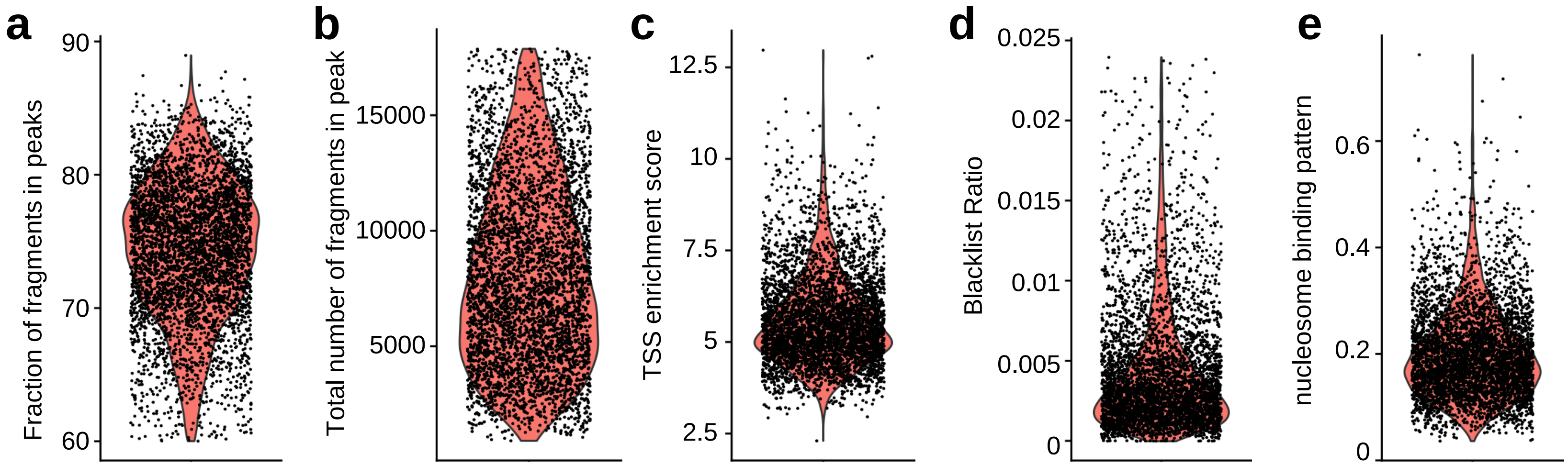

Supplementary Figure 2. Quality control strategy of scATAC-seq of WT mTECs.
Data quality control was carried out according to the value of the fraction of fragments in peaks (**a**), the total number of fragments in the peak region (**b**), the ratio of reads in the "blacklist" region (**c**), the transcription start site (TSS) enrichment score (**d**) and the nucleosome binding pattern (**e**).

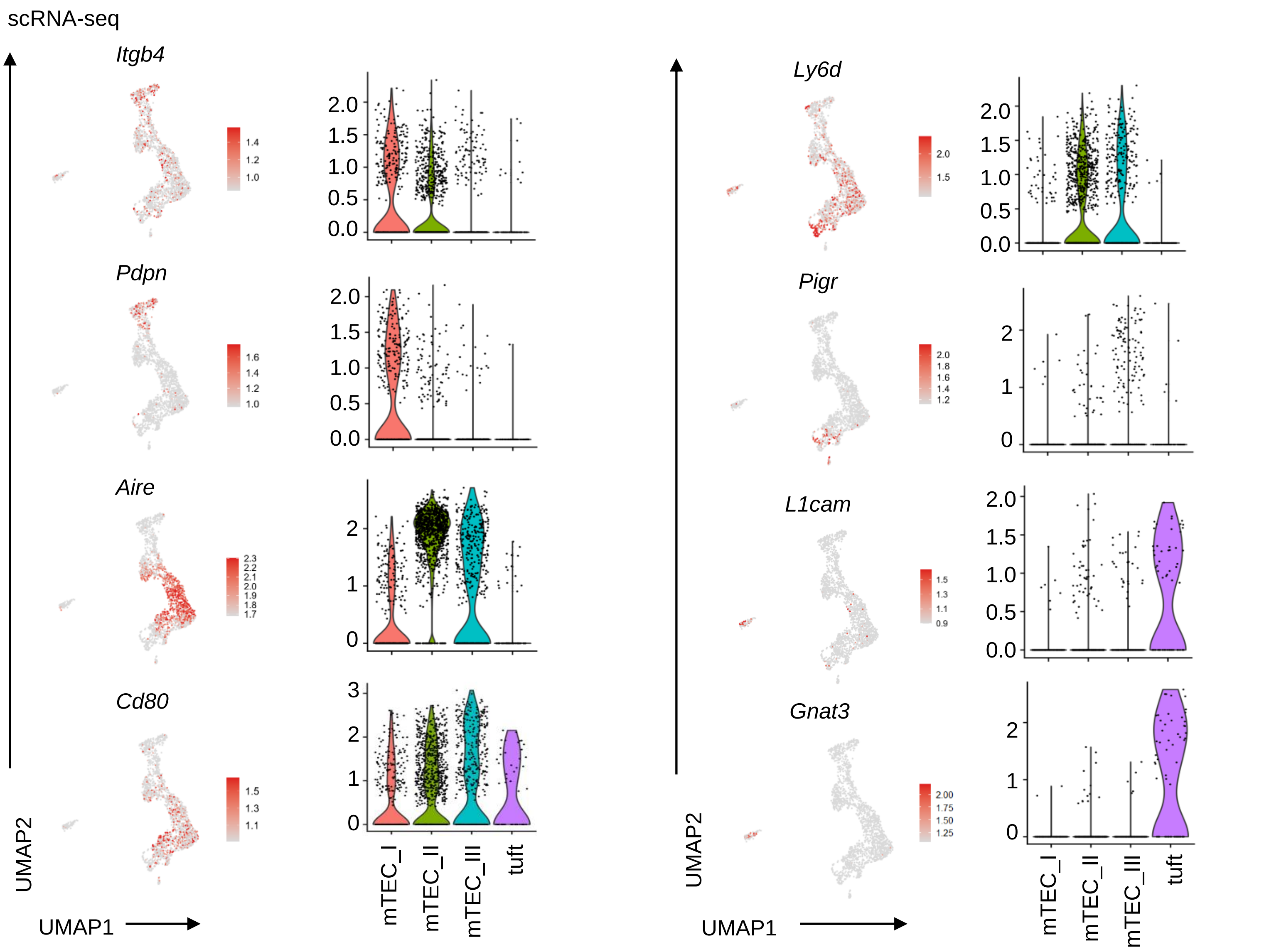

Supplementary Figure 3. Marker gene expression of the defined mTEC populations in scRNA-seq. The gene expression of Itgb4 and Pdpn (mTEC I); Aire, Fezf2, and Cd80 (mTEC II); Pigr and Ly6d (mTEC III); and L1cam, and Gnat3 (thymic tuft cells) as detected by scRNA-seq assay. The red scale in UMAP plot indicate the level of gene expression. The colors in violins indicate different cell types of mTECs.

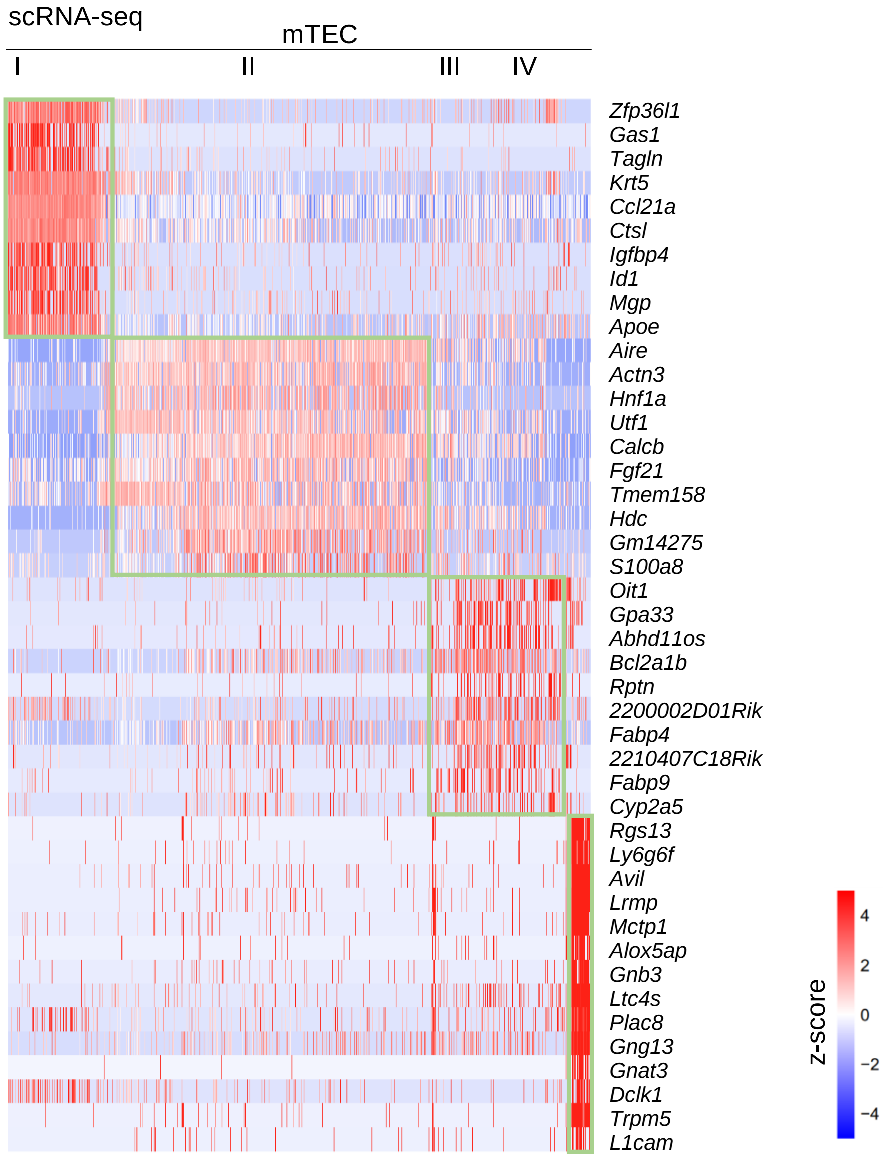

Supplementary Figure 4. Gene expression profile of defined mTEC populations in scRNA-seq. Gene expression profile of mTEC populations, including mTEC I-III and thymic tuft cells. The color scale indicates the level of gene expression. Red represents relatively high expression and blue represents relatively low expression.

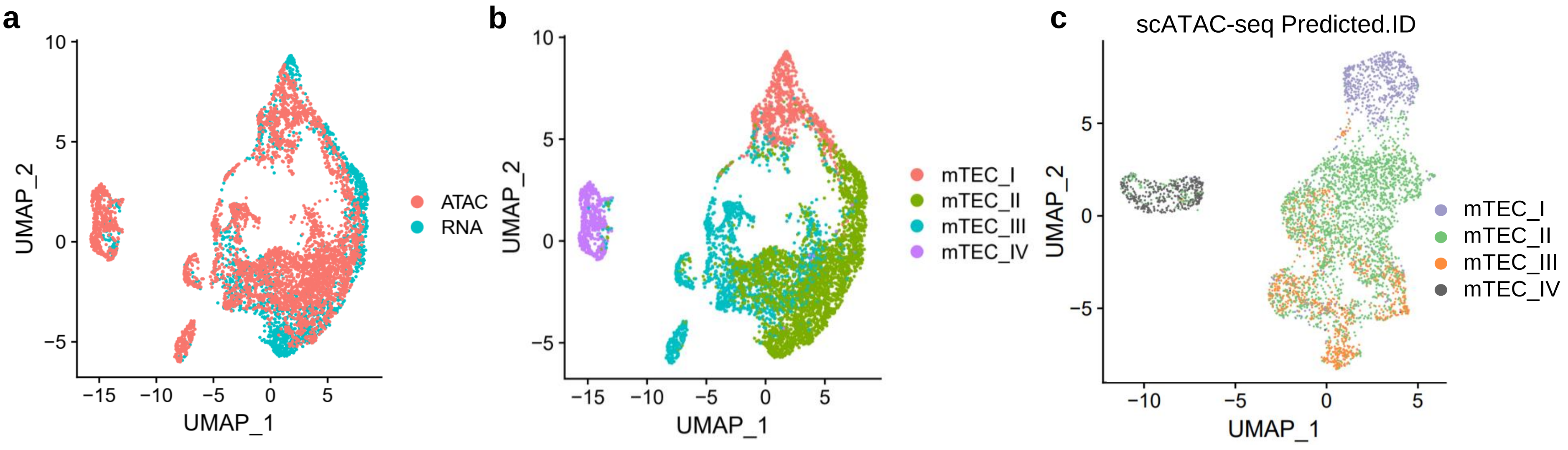

Supplementary Figure 5. Strategy of coembedding scRNA-seq and scATAC-seq data.
(**a**) UMAP visualization of scRNA-seq and scATAC-seq coembedding analysis. Colors representing sequencing techniques. (**b**) UMAP visualization of scRNA-seq and scATAC-seq coembedding analysis. Colors representing cell types. (**c**) UMAP visualization of WT scATAC-seq data. Colors represent the cell type predicted by scRNA-seq.

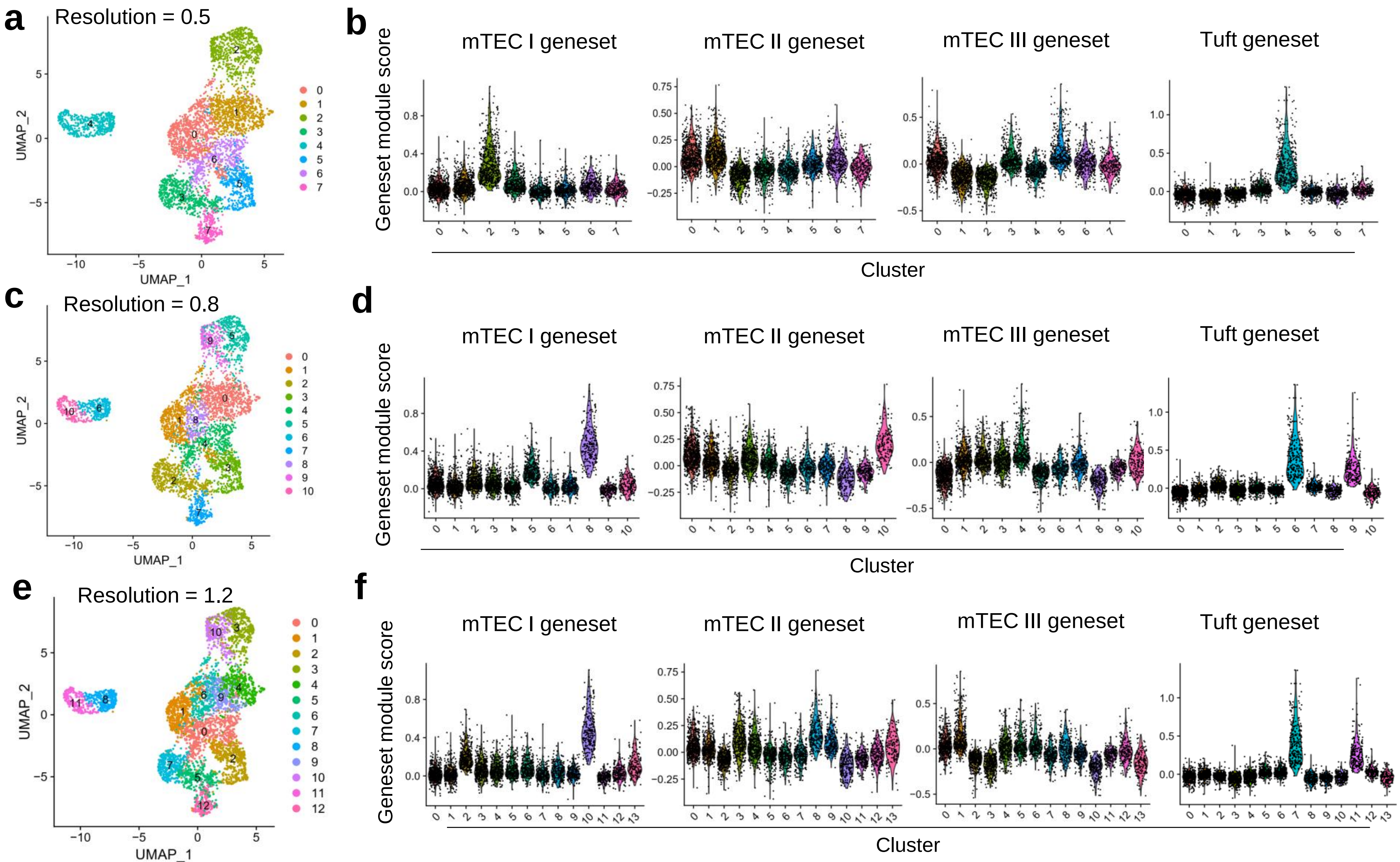

Supplementary Figure 6. Scoring of the cell-specific gene set of WT scATAC-seq at different resolutions. (**a**) scATAC-seq of WT mTECs was divided into 8 color-labeled groups at 0.5 resolution. (**b**) Module scores of mTEC I, mTEC II, mTEC III and tuft feature gene sets in 8 subsets at 0.5 resolution. (**c**) scATAC-seq of WT mTECs was divided into 11 color-labeled groups at 0.8 resolution. (**d**) Module scores of mTEC I, mTEC II, mTEC III and tuft feature gene sets in 11 subsets at 0.8 resolution. (**e**) scATAC-seq of WT mTECs was divided into 13 color-labeled groups at 1.2 resolution. (**f**) Module scores of mTEC I, mTEC II, mTEC III and tuft feature gene sets in 13 subsets at 1.2 resolution. Colors indicate different populations in the resolution of 0.5, 0.8 and 1.2.

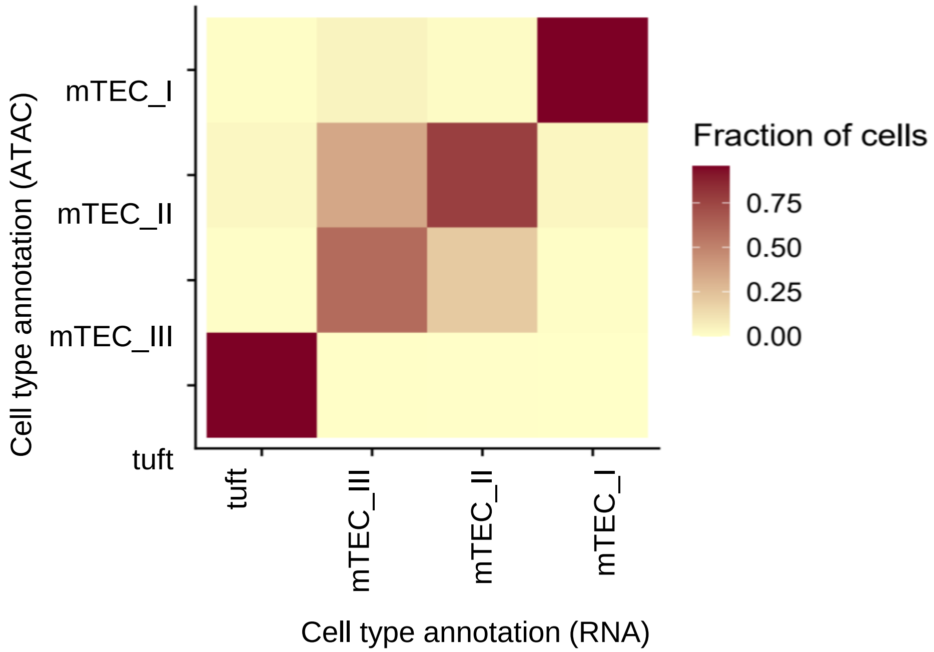

Supplementary Figure 7. Correlation between scATAC-seq annotated cell types and predicted cell types.

The correlation analysis between the cell types annotated by scATAC-seq and predicted by scRNA-seq was based on the fraction of cells identified as the same cell type.

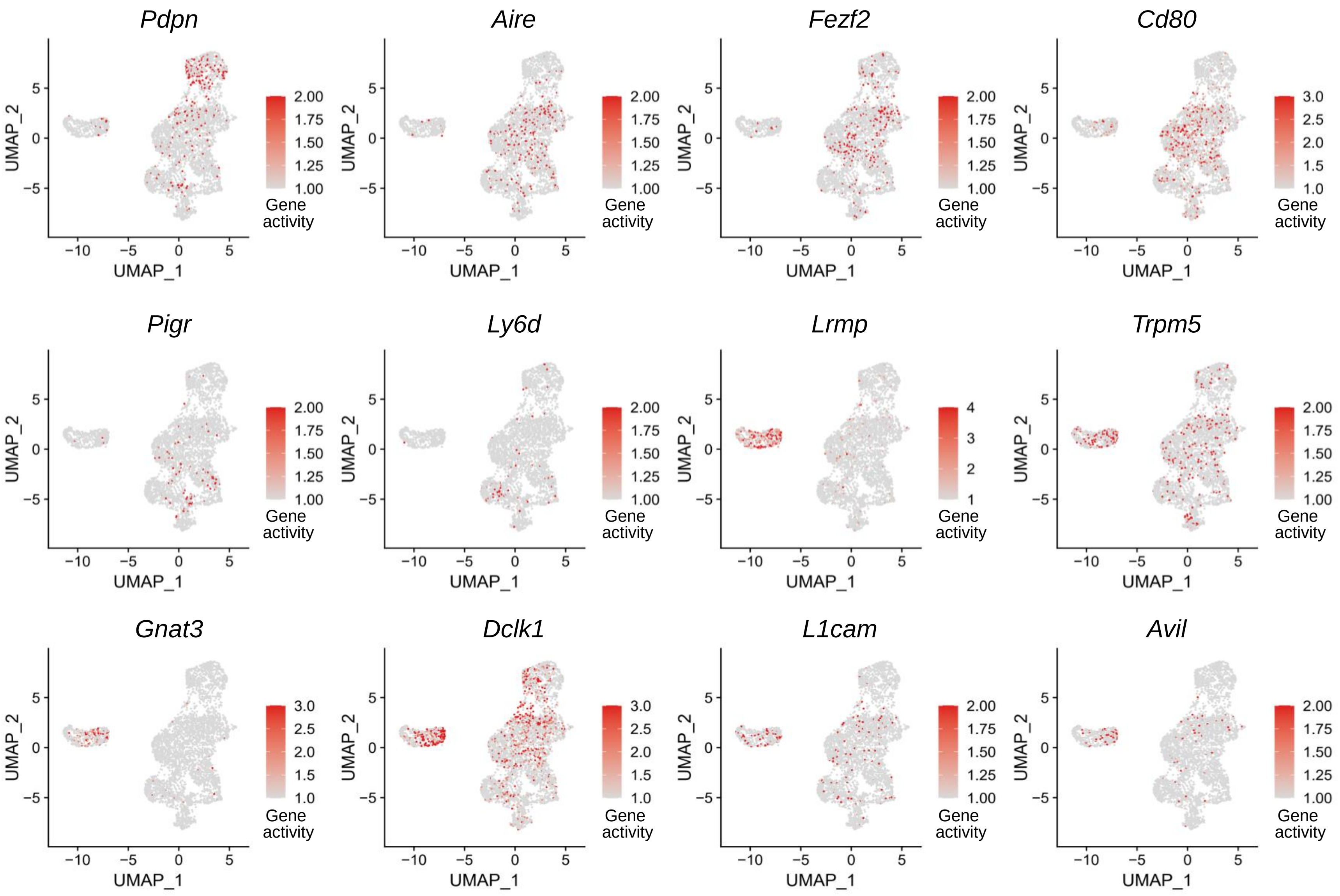

Supplementary Figure 8. Marker gene activity of the defined mTEC populations in scATAC-seq.
The gene activity of *Pdpn, Aire, Fezf, Cd80, Pigr, Ly6d, Lrmp, Trpm5, Gnat3, Dclk1, L1cam* and *Avil* in scATAC-seq. The red scale in UMAP plot indicate the level of gene activity.

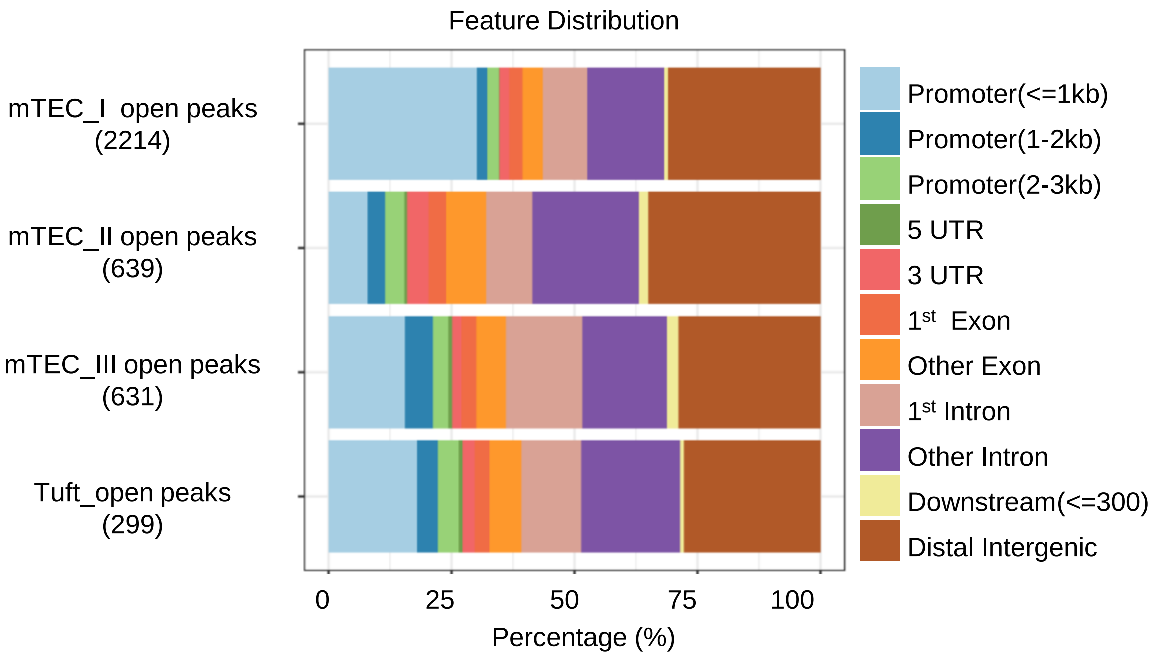

Supplementary Figure 9. Distribution of the peaks of defined mTEC populations in the genome region.

The distribution of the open peaks in the promoter, UTR, exon, intron and distal ranges in mTEC I-III and thymic tuft cells as detected by scATAC-seq. The x-axis represents the proportion of peak numbers in different regions and colors represent different regions of the genome.

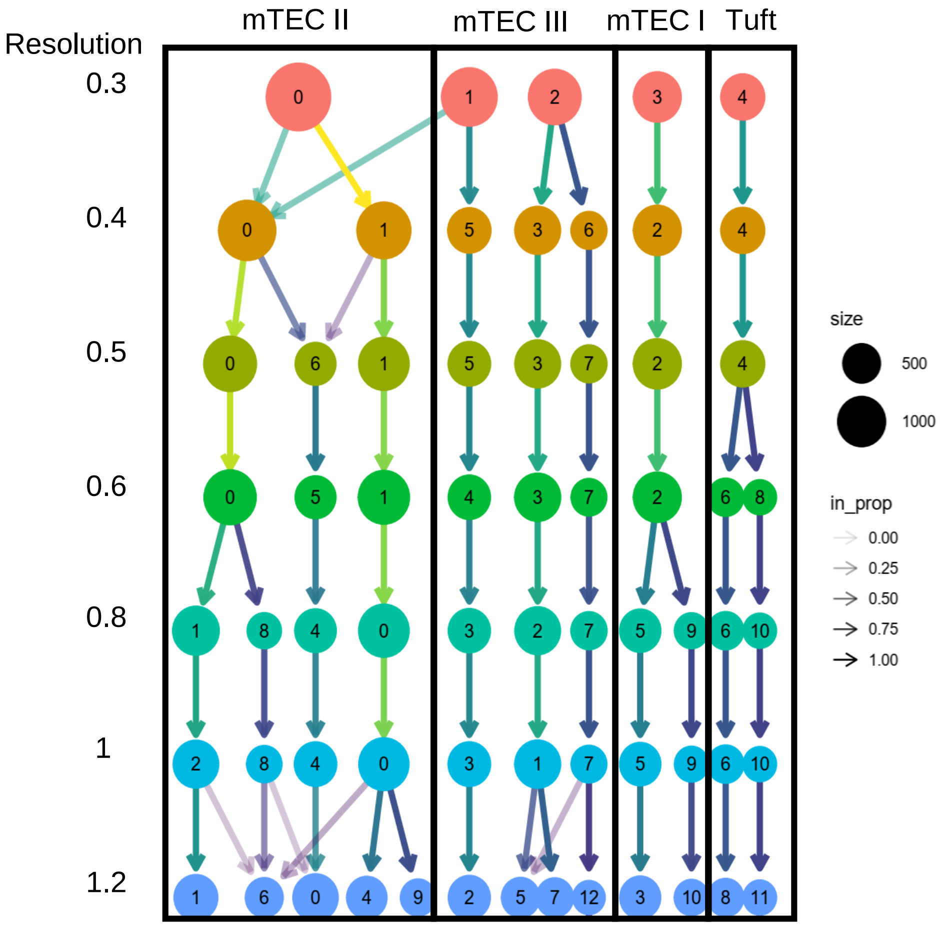

Supplementary Figure 10. Visualization of the distribution of scATAC-seq cells between clusters at different resolutions.

Distribution of mTEC I, mTEC II, mTEC III and tuft cells in scATAC-seq at resolutions of 0.3, 0.4, 0.5, 0.6, 0.8, 1, and 1.2. Colors indicate different resolutions and node size indicate cell numbers.

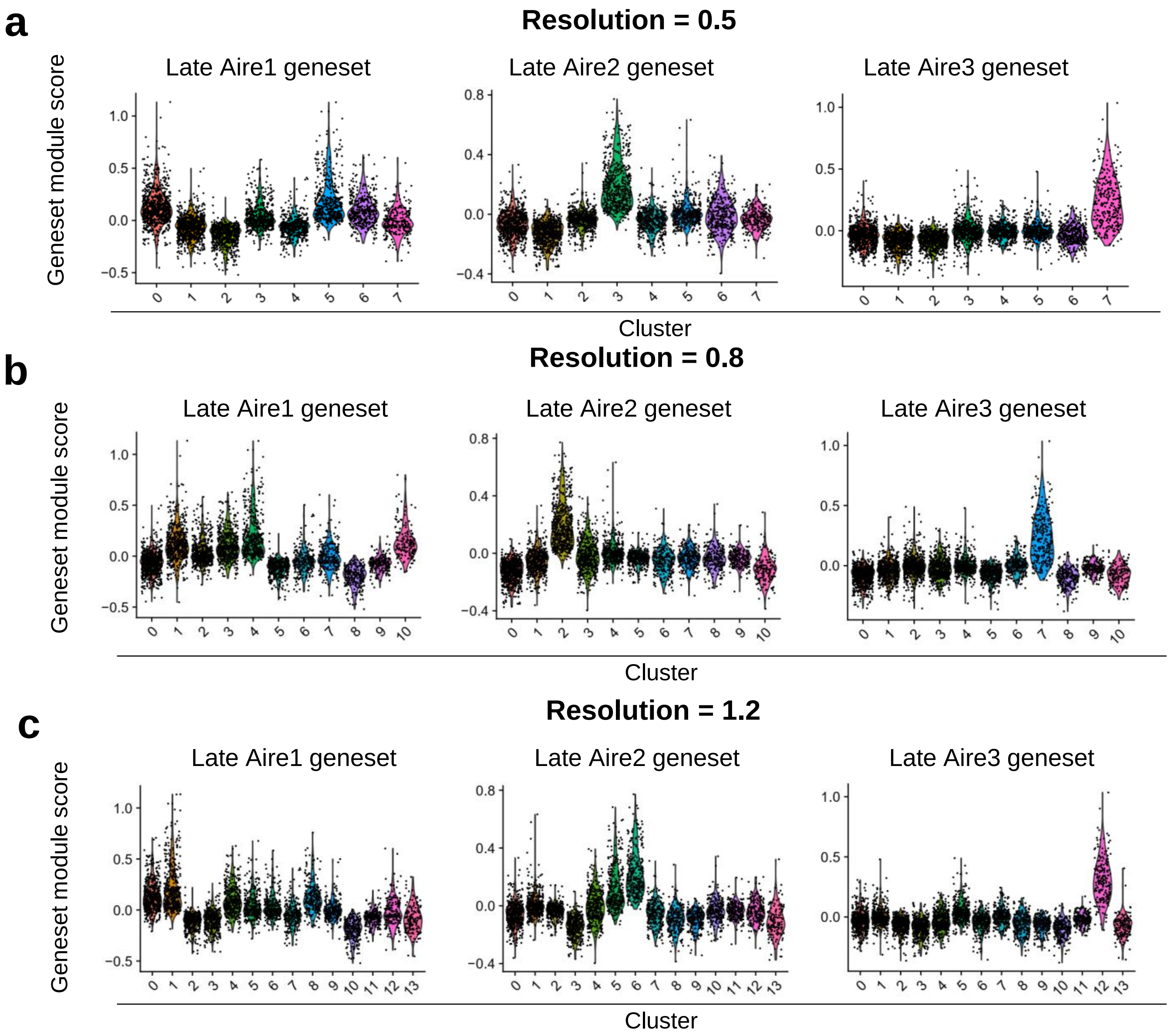

Supplementary Figure 11. Scoring of the Late Aire 1-3 specific gene set of WT scATAC-seq at different resolutions.

(**a**) Module scores of the Late Aire 1, Late Aire 2 and Late Aire 3 feature gene sets in 8 subsets at 0.5 resolution. (b) Module scores of the Late Aire 1, Late Aire 2 and Late Aire 3 feature gene sets in 11 subsets at 0.8 resolution. (c) Module scores of the Late Aire 1, Late Aire 2 and Late Aire 3 feature gene sets in 13 subsets at 1.2 resolution.

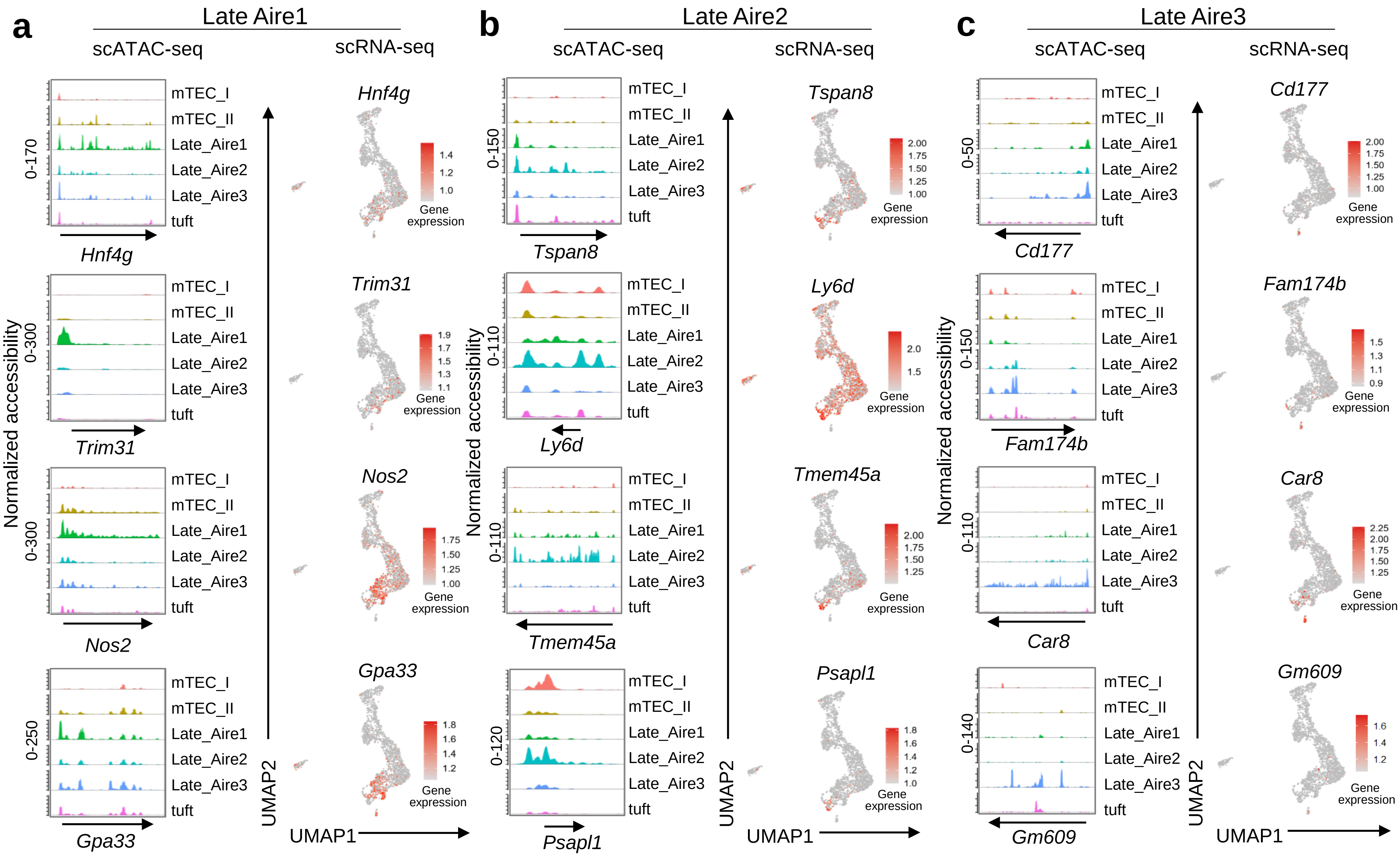

Supplementary Figure 12. Marker gene activity and expression of the three Late Aire populations detected by scATAC-seq and scRNA-seq assays.

Marker gene activity and expression in Late Aire 1 (**a**), Late Aire 2 (**b**) and Late Aire 3 (**c**) was detected by scATAC-seq (coverage plot) and scRNA-seq (feature plot) assays. The colors in genome tracks indicate different cell types of mTECs assayed by scATAC-seq. The arrow indicates the length and direction of the genes detected by scATAC-seq. The red scale in UMAP plot indicate the level of gene expression assayed by scRNA-seq.

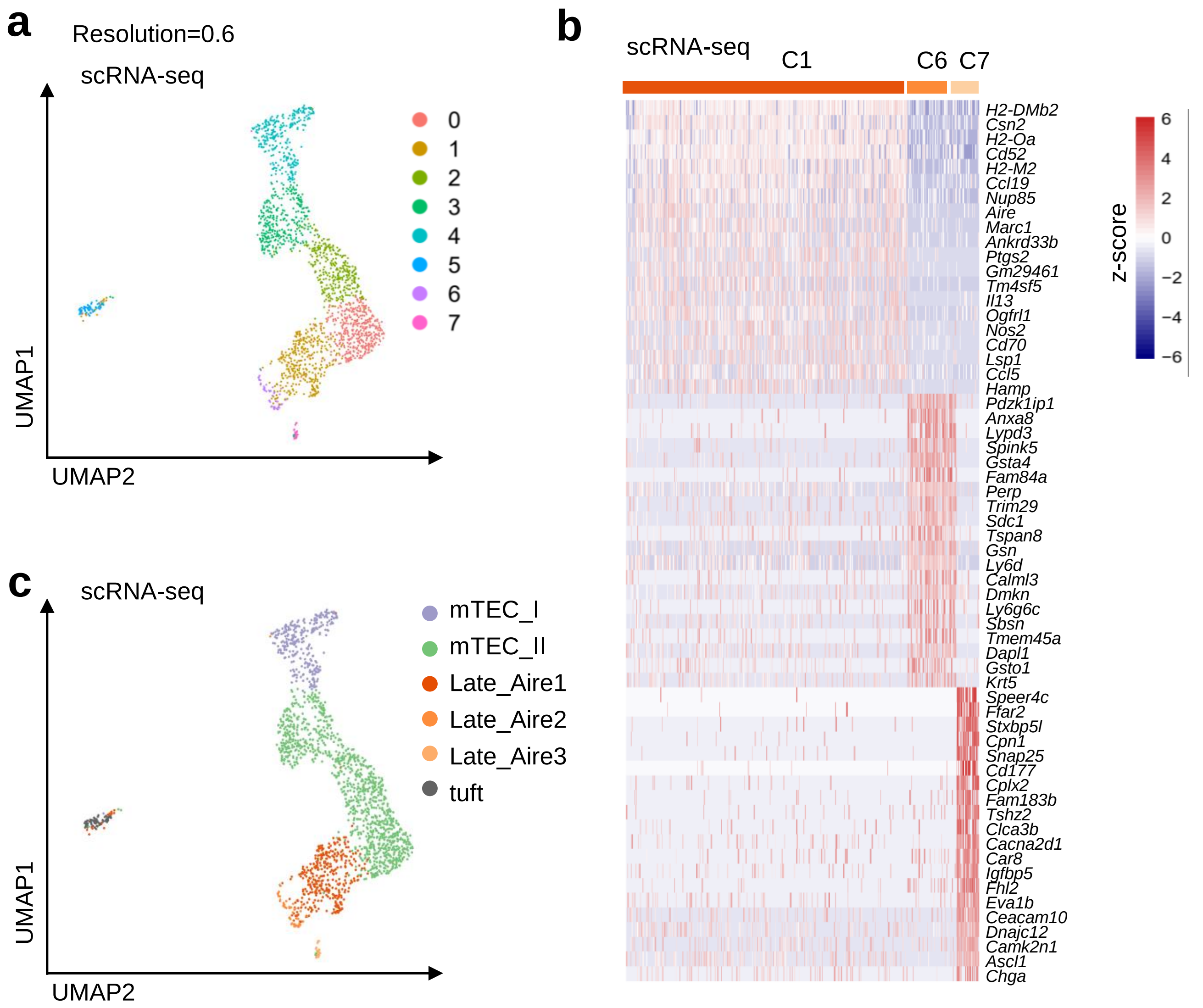

Supplementary Figure 13. Gene expression profiling of the three Late Aire populations in scRNA-seq. (**a**) UMAP visualization of scRNA-seq in resolution 0.6. Colors represent cell types in resolution 0.6. (**b**) Gene expression profiles of C1, C6 and C7 as detected by scRNA-seq assays. Color scale indicates the level of gene expression. Red represents relatively high expression and blue represents relatively low expression. (**c**) UMAP visualization of scRNA-seq colored by six identified mTEC populations. Colors represent cell types of mTECs.

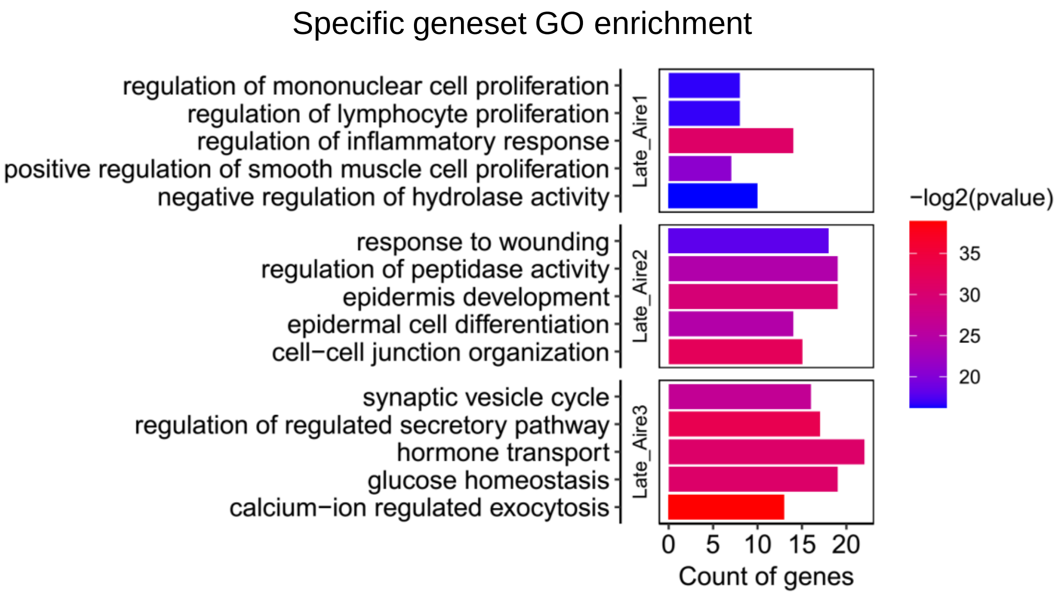

Supplementary Figure 14. GO enrichment analysis of the three Late Aire populations by scRNA-seq assay.

The marker genes of Late Aire 1-3 was used for GO enrichment analysis. Top5 GO terms of Late Aire 1-3 selected according to p value were provided here. Color scale indicates –log2(pvalue).

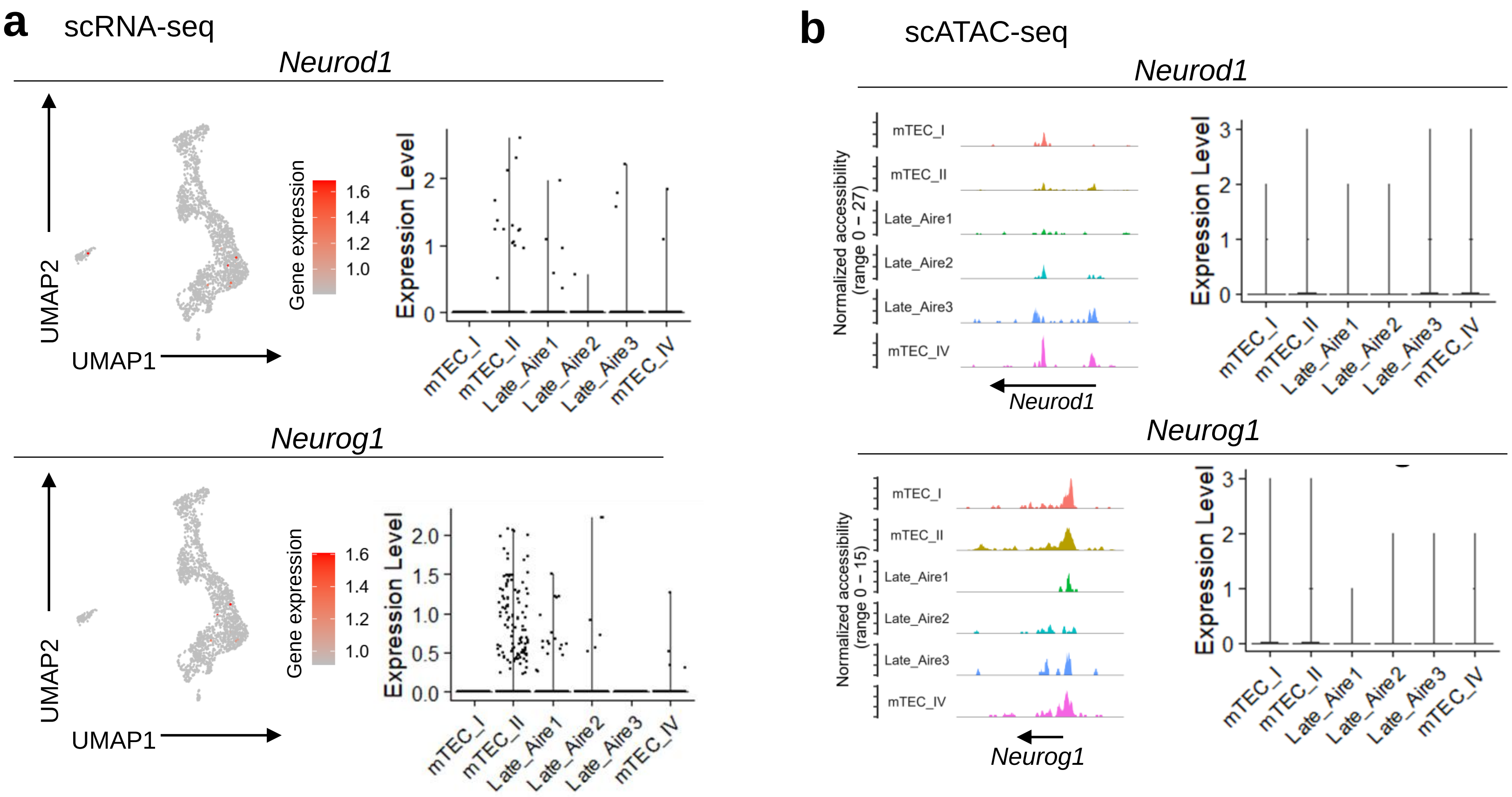

Supplementary Figure 15. The expression and activity of genes associated with neuro TECs assayed by scRNA-seq and scATAC-seq.

(**a**) The expression of neuro TECs markers in mTEC subsets was detected by scRNA-seq. The red scale in UMAP plot indicate the gene expression level of scRNA-seq. (**b**) The chromatin accessibility of neuro TECs markers in mTEC subsets was detected by scATAC-seq. The colors in genome tracks indicate different cell types of mTECs. The arrow indicates the length and direction of the genes detected by scATAC-seq.

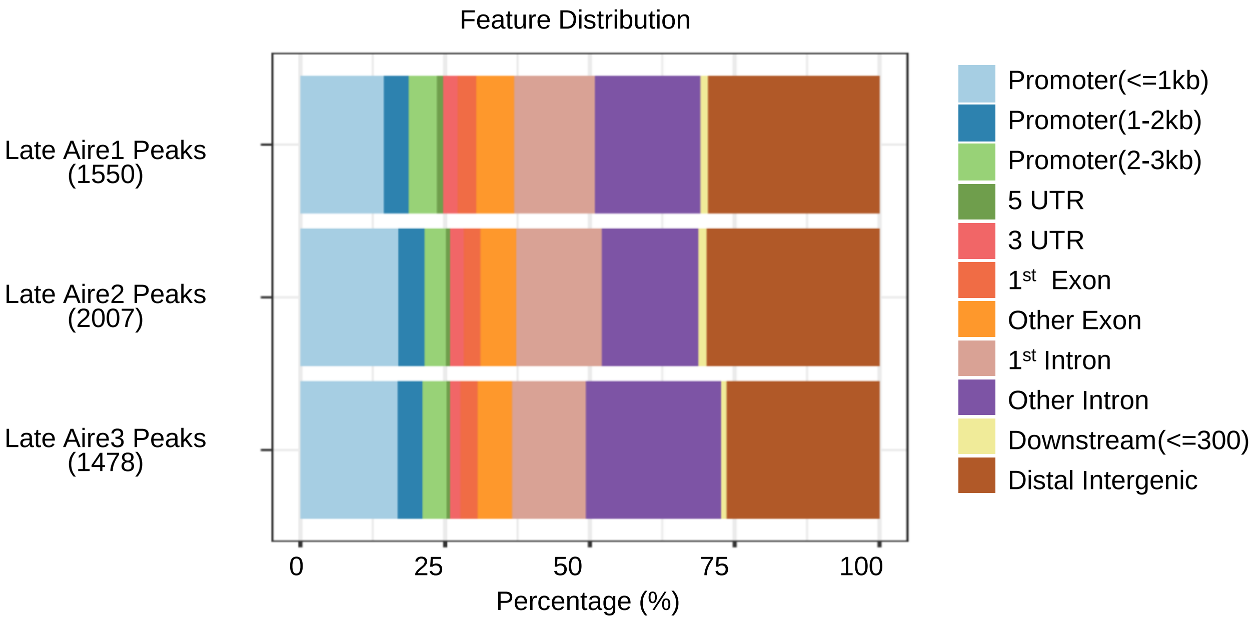

Supplementary Figure 16. Distribution of the peak of the three Late Aire populations in the genome region.

The distribution of the open peaks in the promoter, UTR, exon, intron and distal ranges in Late Aire 1-3 cells as assayed by scATAC-seq. The x-axis represents the proportion of peak numbers in different regions and colors represent different regions of the genome.

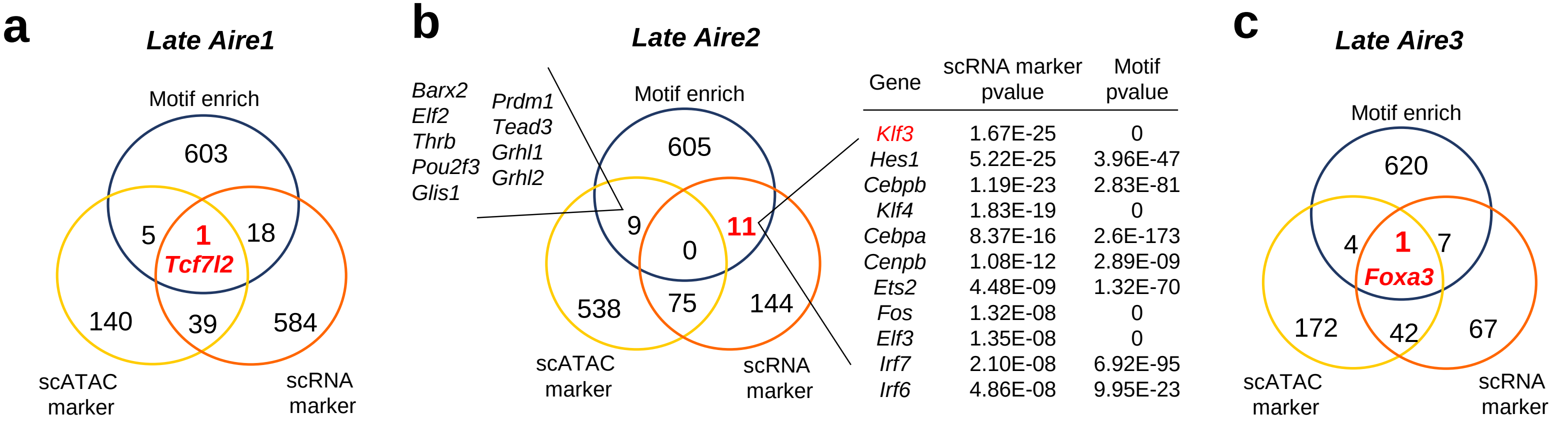

Gene	scRNA marker pvalue	Motif pvalue
Klf3	1.67E-25	0
Hes1	5.22E-25	3.96E-47
Cebpb	1.19E-23	2.83E-81
Klf4	1.83E-19	0
Cebpa	8.37E-16	2.6E-173
Cenpb	1.08E-12	2.89E-09
Ets2	4.48E-09	1.32E-70
Fos	1.32E-08	0
Elf3	1.35E-08	0
Irf7	2.10E-08	6.92E-95
Irf6	4.86E-08	9.95E-23

Supplementary Figure 17. Enrichment of specific transcription factors in the three Late Aire populations.

The highly expressed genes detected by transcription factor motif enrichment, scRNA-seq and scATAC-seq assays were matched, and the key transcription factors in Late Aire 1 (**a**), Late Aire 2 (**b**) and Late Aire 3 (**c**) were identified.

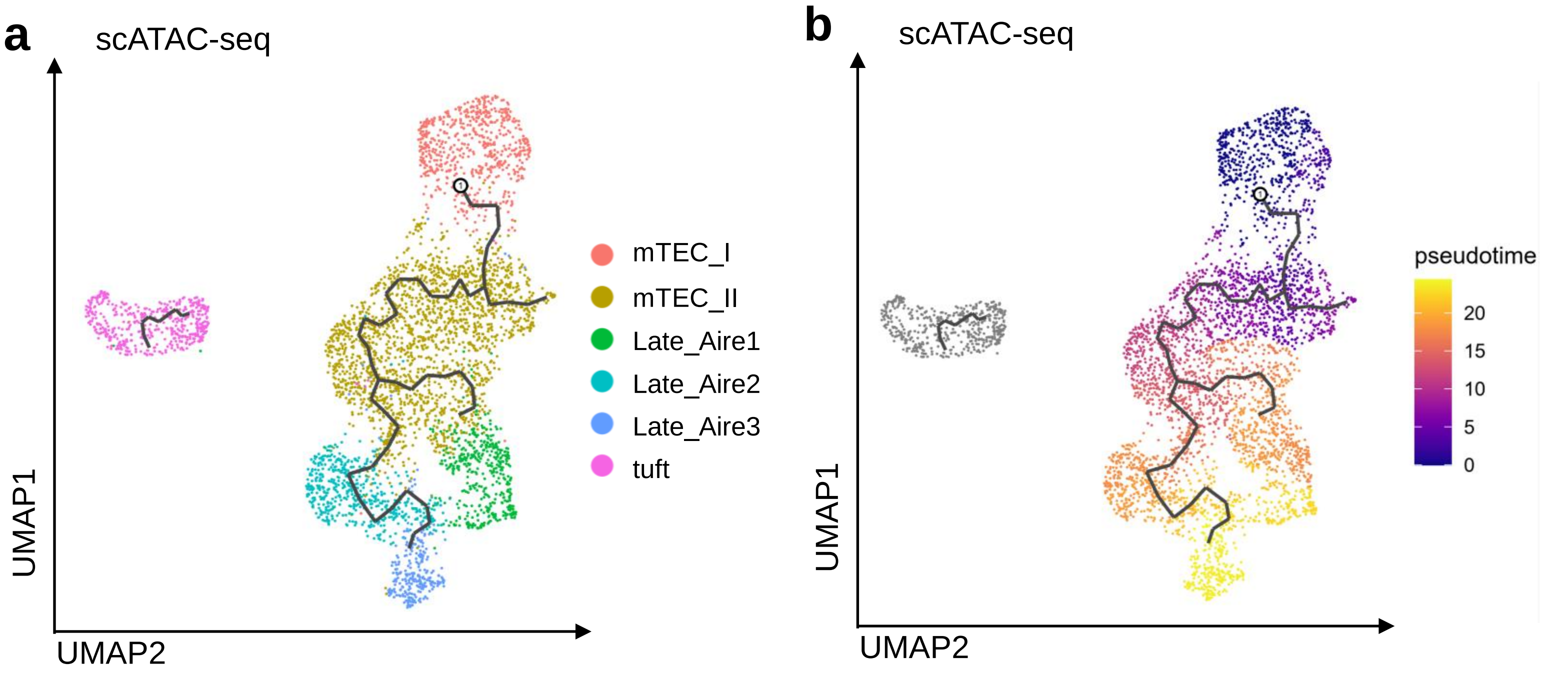

Supplementary Figure 18. Trajectory analysis of scATAC-seq.
(**a**) UMAP visualization of scATAC-seq trajectory colored by subsets of mTEC I, mTEC II, Late Aire 1, Late Aire 2, Late Aire 3, and thymic tuft cells. (**b**) UMAP visualization of scATAC-seq trajectory colored based on the pseudotime.

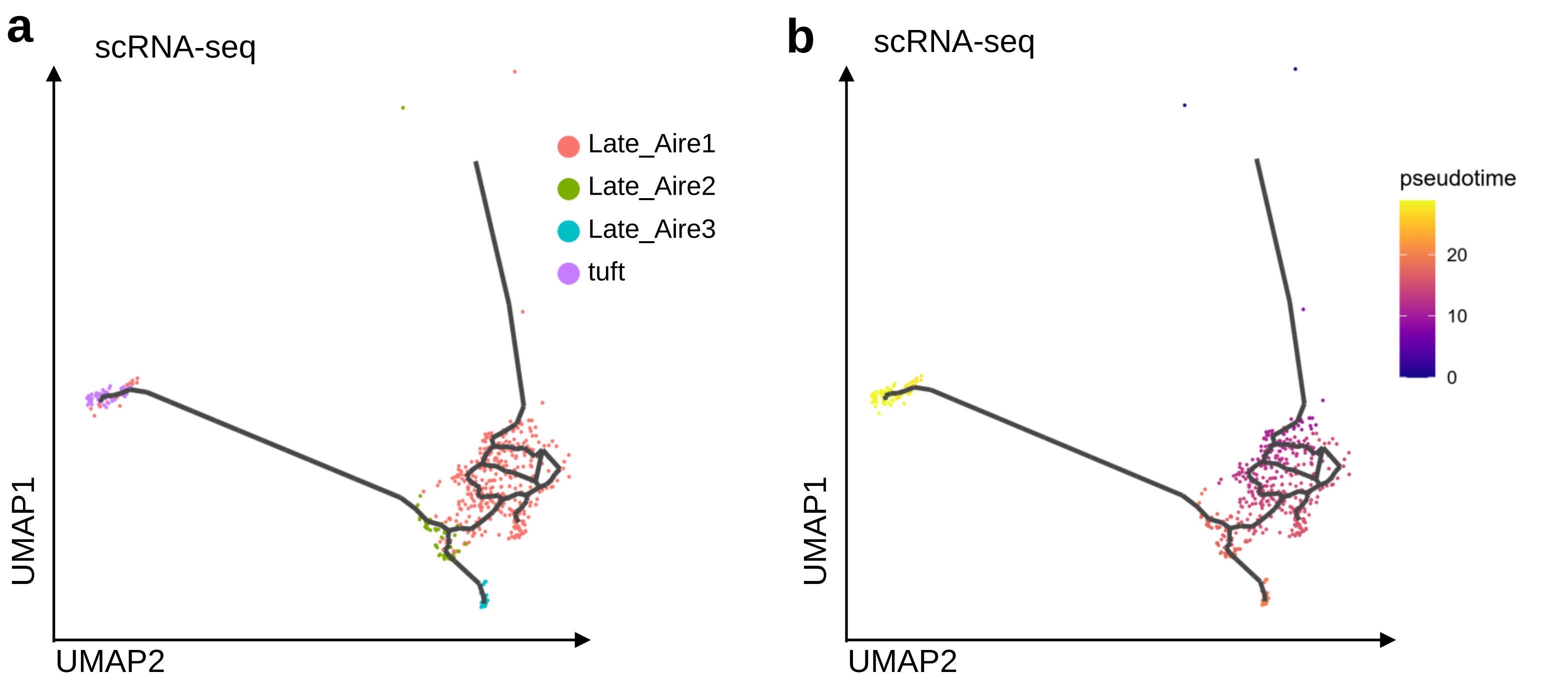

Supplementary Figure 19. Trajectory analysis of scRNA-seq.
(**a**) UMAP visualization of scRNA-seq trajectory including Late Aire 1, Late Aire 2, Late Aire 3, and thymic tuft cells. Color represents the cell type. (**b**) UMAP visualization of scRNA-seq trajectory including Late Aire 1, Late Aire 2, Late Aire 3, and thymic tuft cells. Color represents the pseudotime.

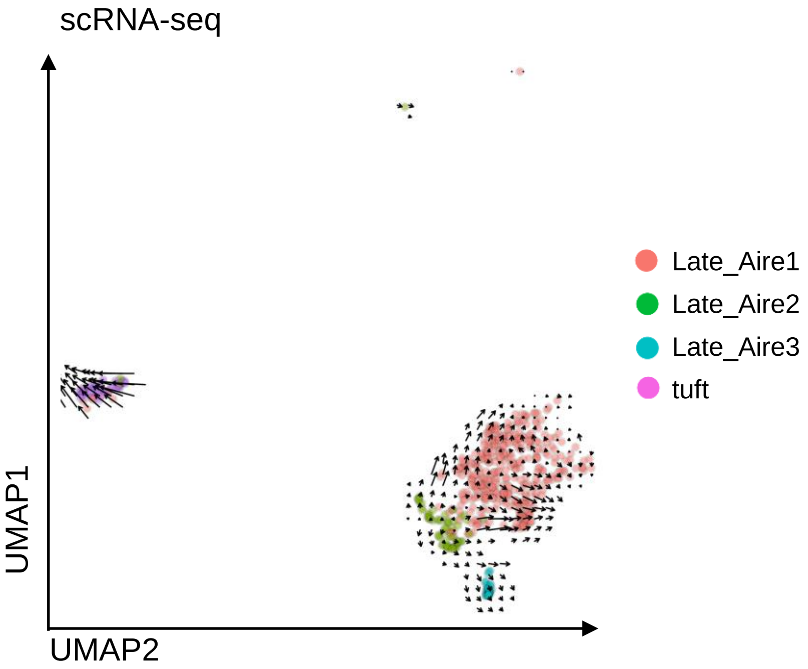

Supplementary Figure 20. RNA velocity analysis of scRNA-seq.
UMAP visualization of RNA velocity including Late Aire 1, Late Aire 2, Late Aire 3, and thymic tuft cells of scRNA-seq. Color represents the cell type.

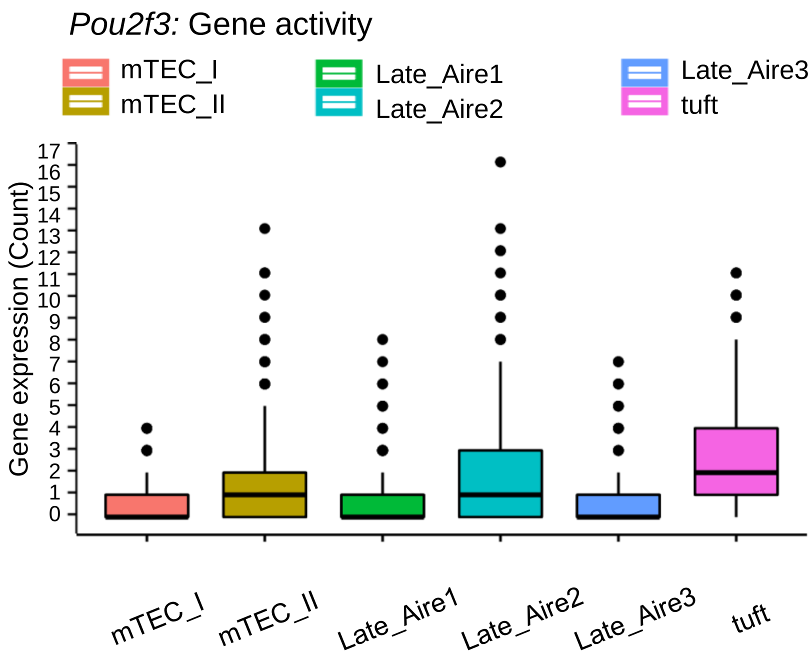

Comparison *P-value*

	mTEC_II	Late_Aire1	Late_Aire2	Late_Aire3	tuft
mTEC_I	**8.7e-10**	0.14	**<2.2e-16**	0.66	**<2.2e-16**
mTEC_II		**<1.5e-11**	**4.1e-10**	**5e-06**	**<2.2e-16**
Late_Aire1			**<2.2e-16**	0.47	**<2.2e-16**
Late_Aire2				**8.8e-14**	**2.9e-15**
Late_Aire3					**<2.2e-16**

Supplementary Figure 21. *Pou2f3* gene activity in mTECs assayed by scATAC-seq.
Expression of *Pou2f3* in scATAC-seq was imputed by a gene activity matrix and was shown as boxplots. *P-values* were derived from accessibility comparison of pairwise cell populations. The numbers marked in red are significantly different (P-value <0.05).

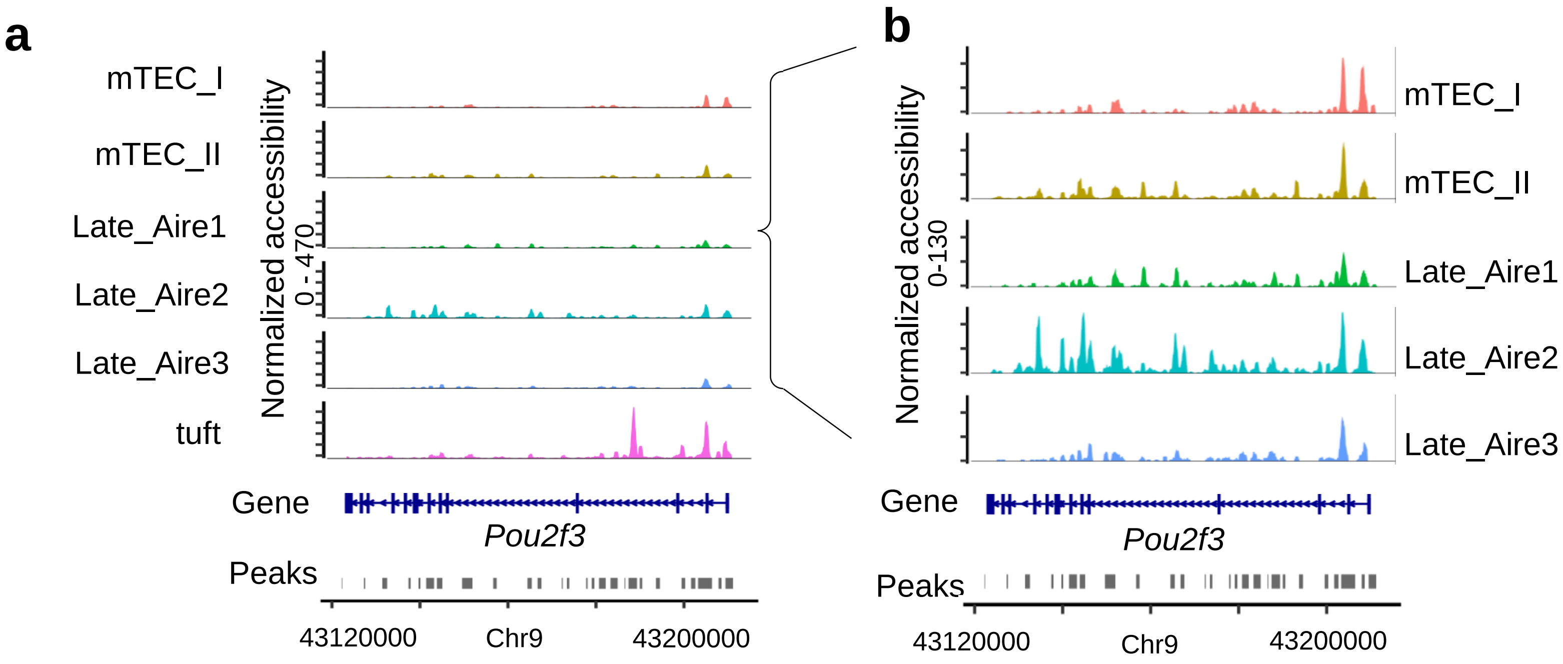

Supplementary Figure 22. The accessibility of *Pou2f3* peaks in mTECs assayed by scATAC-seq.
(**a**) Chromatin accessibility peaks of the *Pou2f3* gene in all mTEC subsets are shown in scATAC-seq tracks. (**b**) Chromatin accessibility peaks of the *Pou2f3* gene in mTEC I, mTEC II, Late Aire 1, Late Aire 2 and Late Aire 3 are shown in scATAC-seq tracks. The colors in genome tracks indicate different cell types of mTECs. The arrow indicates the length and direction of the *Pou2f3* detected by scATAC-seq.

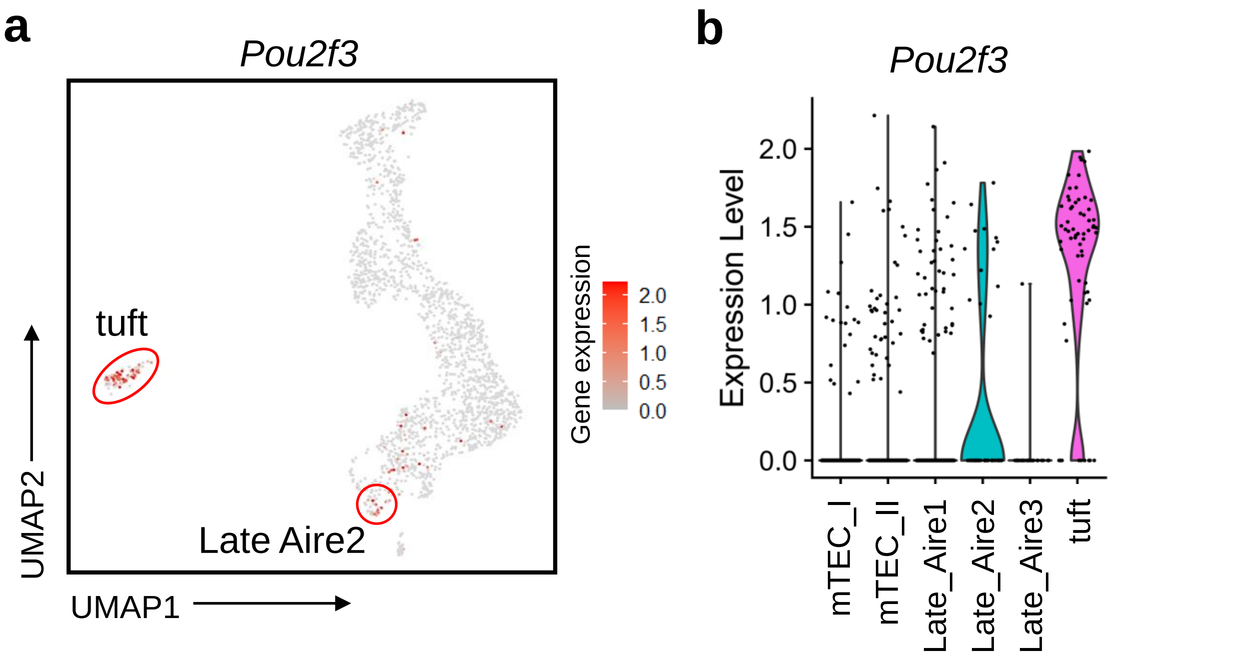

Supplementary Figure 23. *Pou2f3* gene expression in mTECs assayed by scRNA-seq. The expression of *Pou2f3* in WT scRNA-seq was visualized by UMAP plot (**a**) and violin plot (**b**). The red scale in UMAP plot (**a**) indicate the level of gene expression detected by scRNA-seq. The colors in violins indicate different cell types of mTECs.

Pou2f3: TF motif accessibility

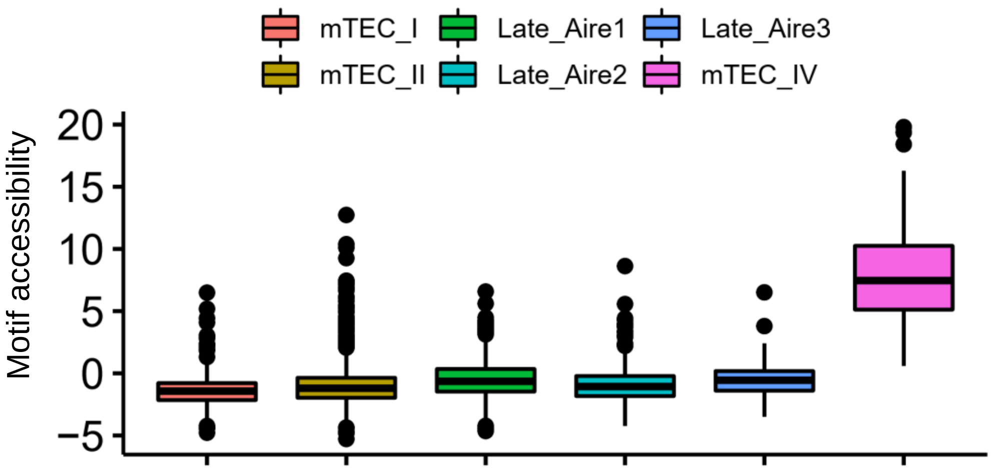

Supplementary Figure 24. *Pou2f3* motif accessibility in mTECs assayed by scATAC-seq. Comparison of the motif accessibility of *Pou2f3* in the mTEC I, mTEC II, Late Aire 1, Late Aire 2, Late Aire 3 and tuft cells. The colors in boxplots indicate different cell types of mTECs.

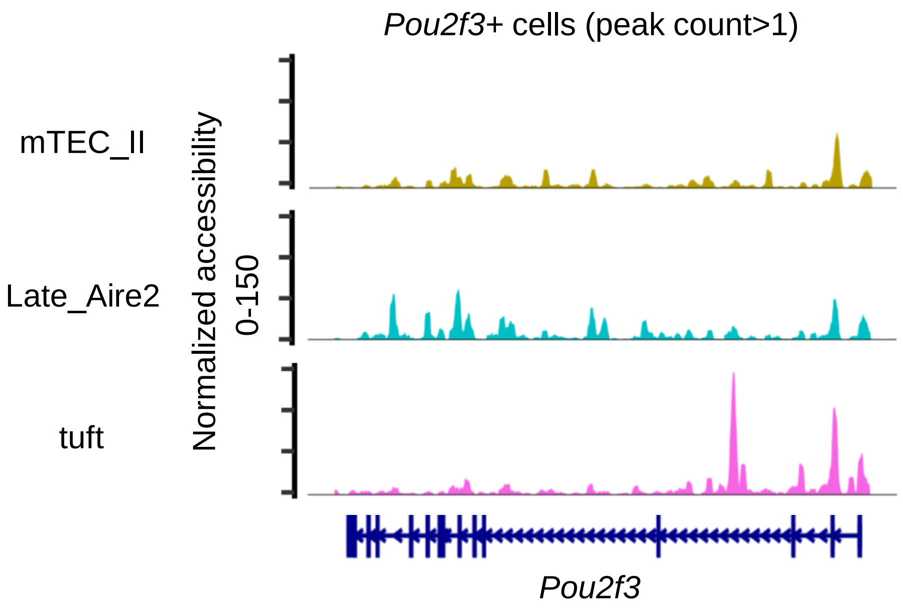

Supplementary Figure 25. Pou2f3 gene expression in Pou2f3+ mTECs assayed by scATAC-seq. Chromatin accessibility peaks of the *Pou2f3* gene in *Pou2f3*+ mTEC II, Late Aire 2 and tuft are shown in scATAC-seq tracks. The colors in genome tracks indicate different cell types of mTECs. The arrow indicates the length and direction of Pou2f3 detected by scATAC-seq.

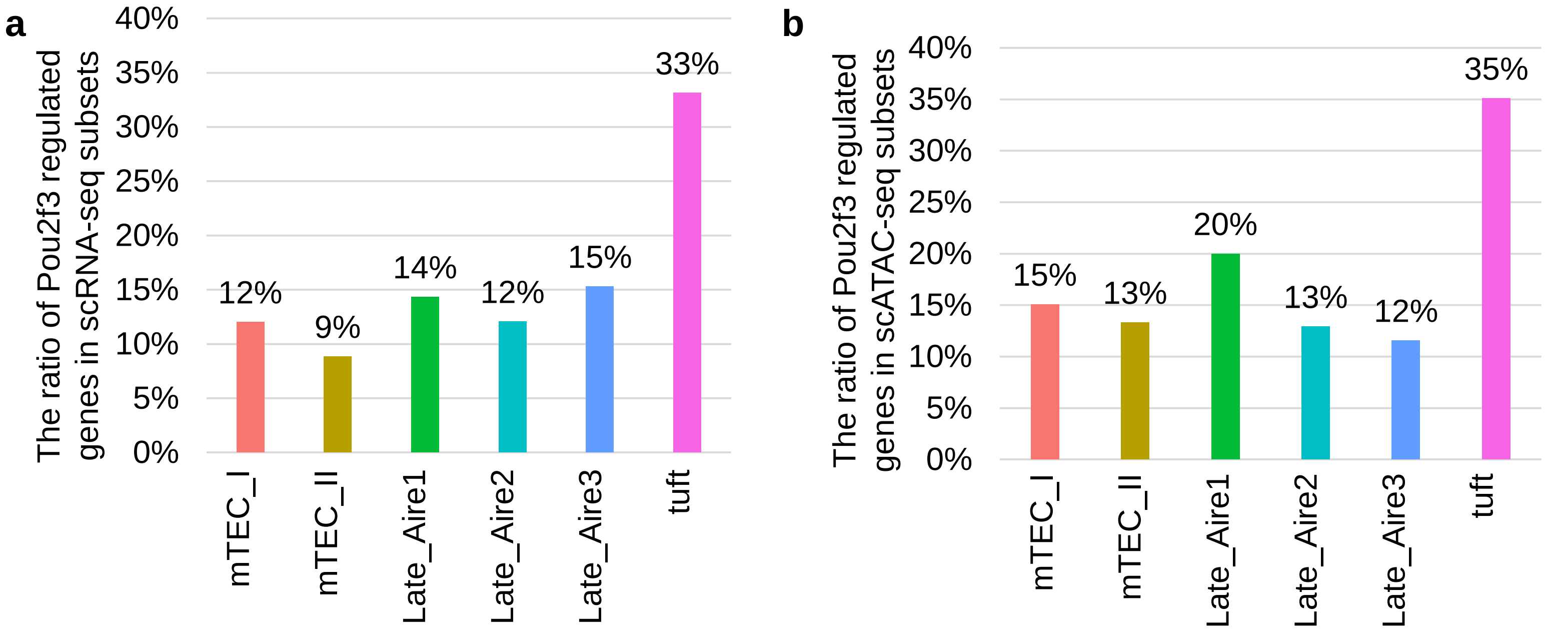

Supplementary Figure 26. Proportion of genes regulated by Pou2f3 in the mTEC population. (**a**) Ratio of Pou2f3-regulated genes in scATAC-seq subsets. (**b**) Ratio of Pou2f3-regulated genes in scRNA-seq subsets. The colors in histogram indicate different cell types of mTECs.

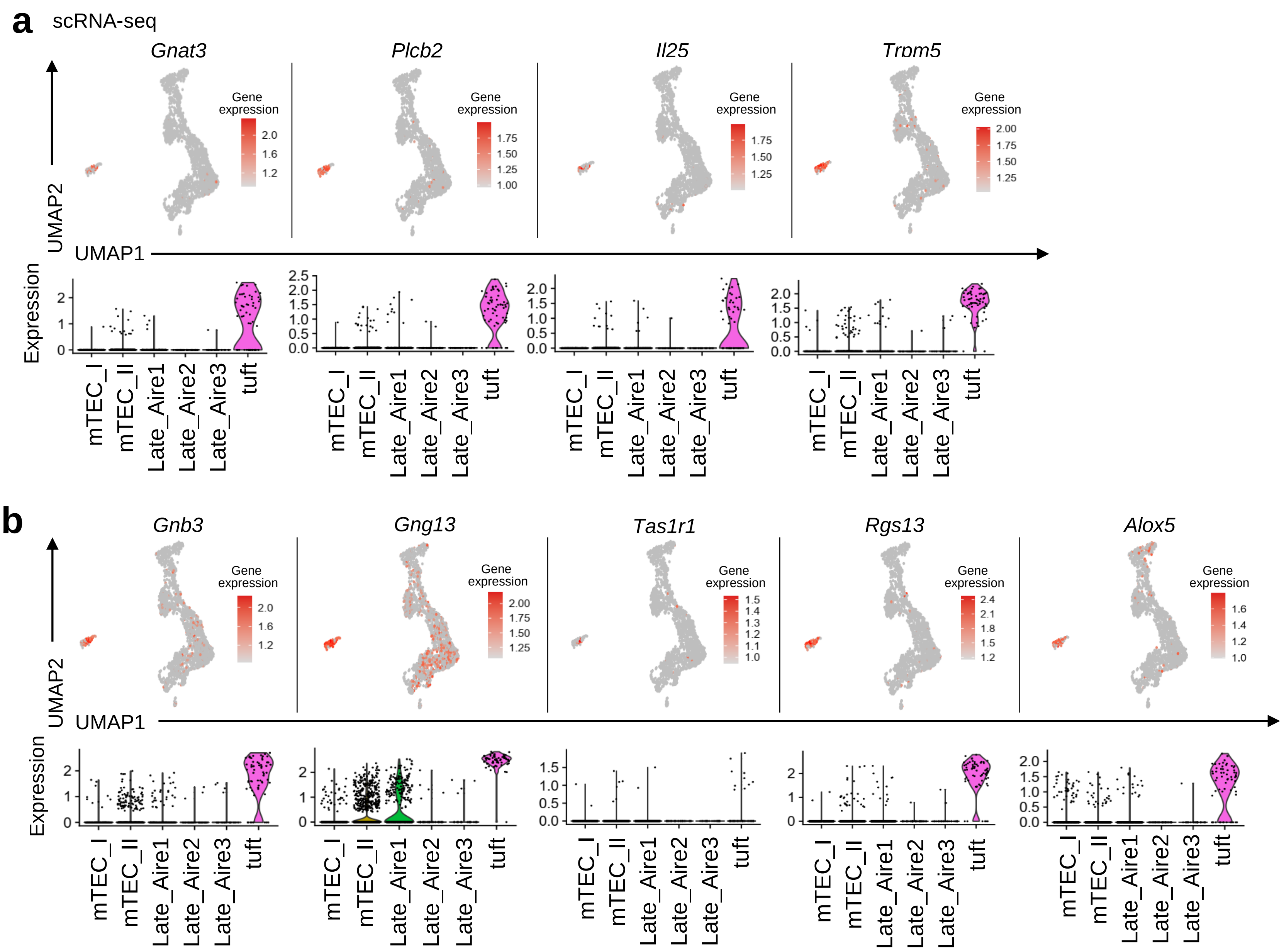

Supplementary Figure 27. Expression of the POU2F3-regulated genes in mTECs as analyzed by scRNA-seq. POU2F3-regulated genes, including *Gnat3*, *Plcb2*, *Il25* and *Trpm5* (**a**), and tuft-associated genes, including *Gnb3*, *Gng13*, *Tas1r1*, *Rgs13* and *Alox5* (**b**), were selectively expressed in thymic tuft cells, as analyzed by scRNA-seq. The red scale in UMAP plot indicate the level of gene expression. The colors in violins indicate different cell types of mTECs.

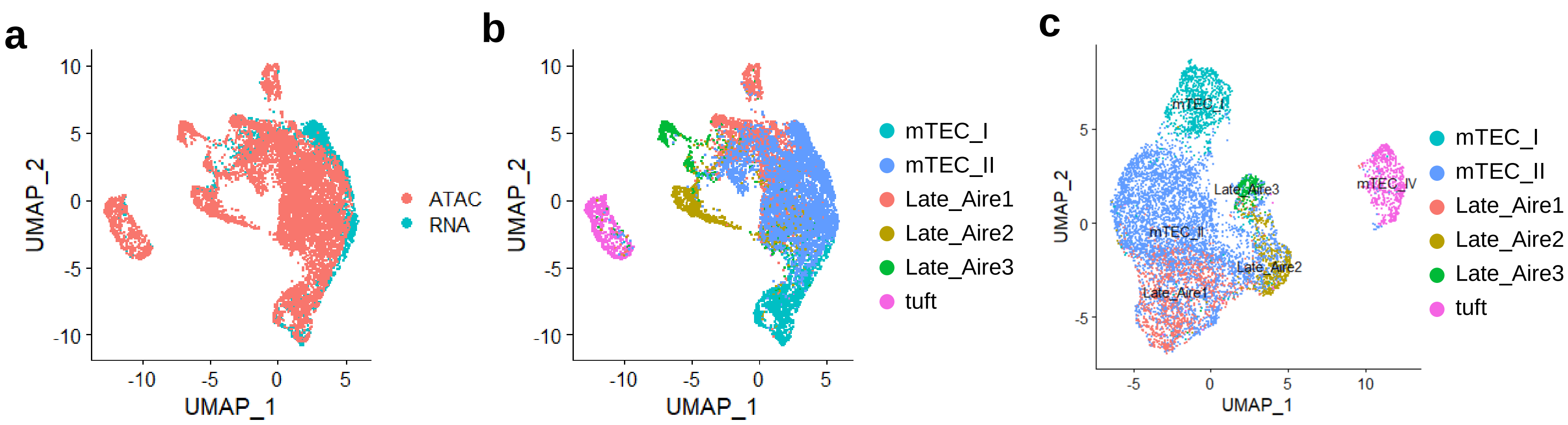

Supplementary Figure 28. Strategy of coembedding scRNA-seq and scATAC-seq data.
(**a**) UMAP visualization of the scRNA-seq and integrated scATAC-seq coembedding analysis. Colors represent the sequencing techniques. (**b**) UMAP visualization of the scRNA-seq and integrated scATAC-seq coembedding analysis. Colors represent the cell type. (**c**) UMAP visualization of integrated scATAC-seq data mixed with WT and Sirt6 cKO mTECs. Colors represent the cell type predicted by scRNA-seq.

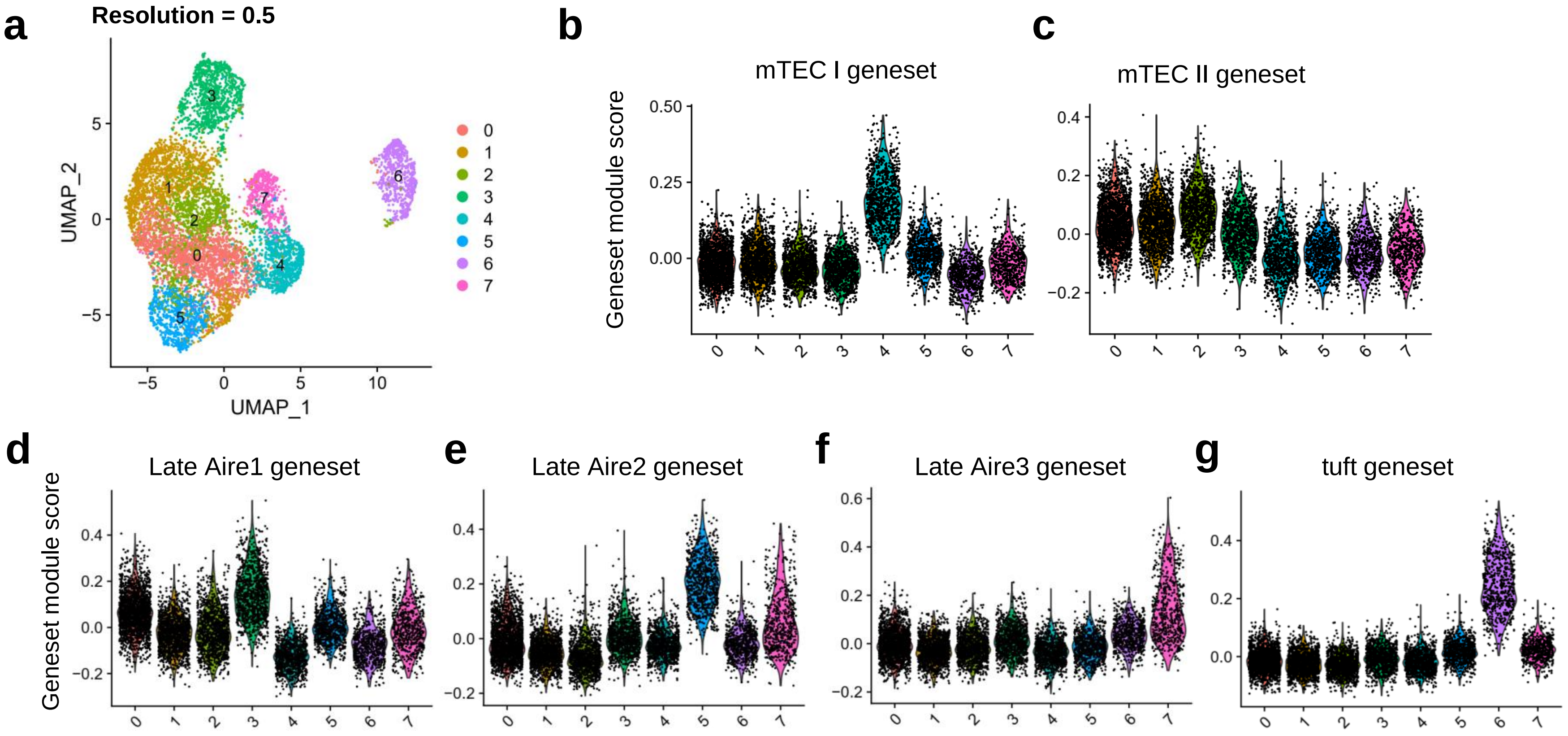

Supplementary Figure 29. Scoring of the cell-specific gene set of integrated scATAC-seq.
(**a**) Integrated scATAC-seq of WT and Sirt6 cKO mTECs was divided into 8 color-labeled groups at 0.5 resolution. Module scores of the feature gene set of mTEC I (**b**), mTEC II (**c**), Late Aire 1 (**d**), Late Aire 2 (**e**), Late Aire 3 (**f**) and tuft (**g**) in 8 subsets at 0.5 resolution.

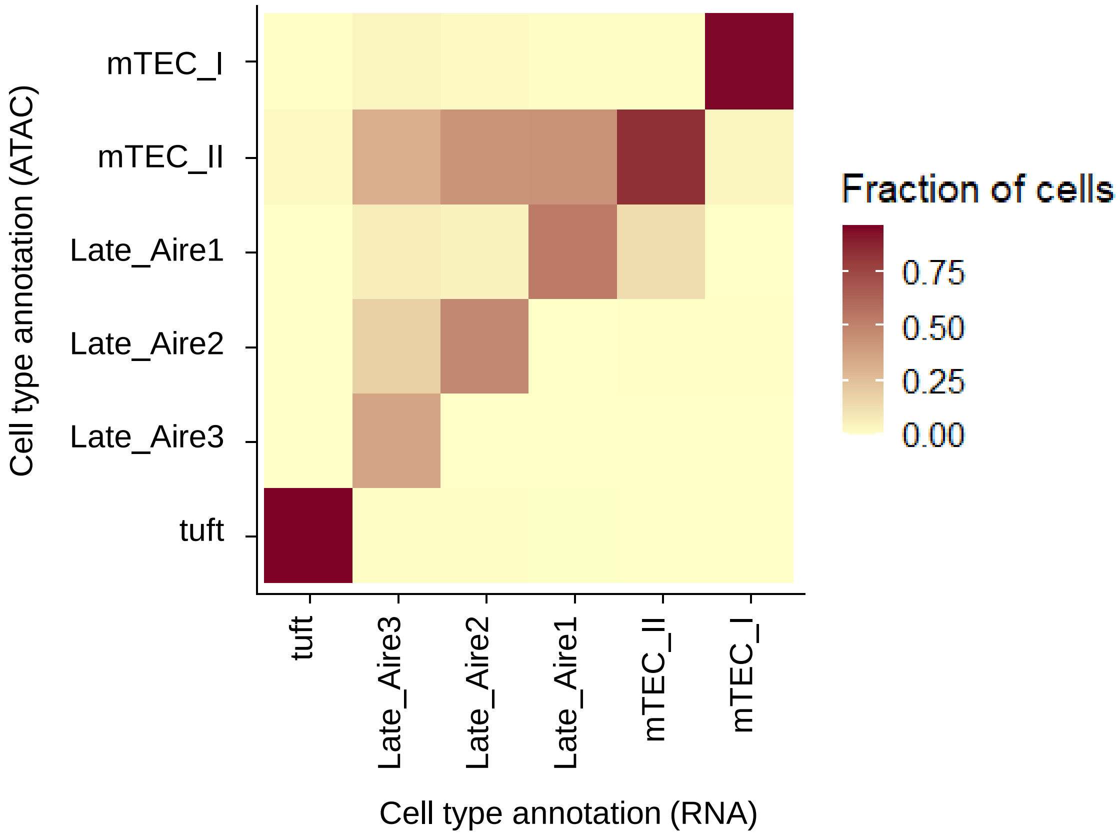

Supplementary Figure 30. Correlation between integrated scATAC-seq annotated cell types and predicted cell types.

The correlation analysis between the cell types annotated by integrated scATAC-seq and predicted by scRNA-seq was based on the fraction of cells identified as the same cell type.

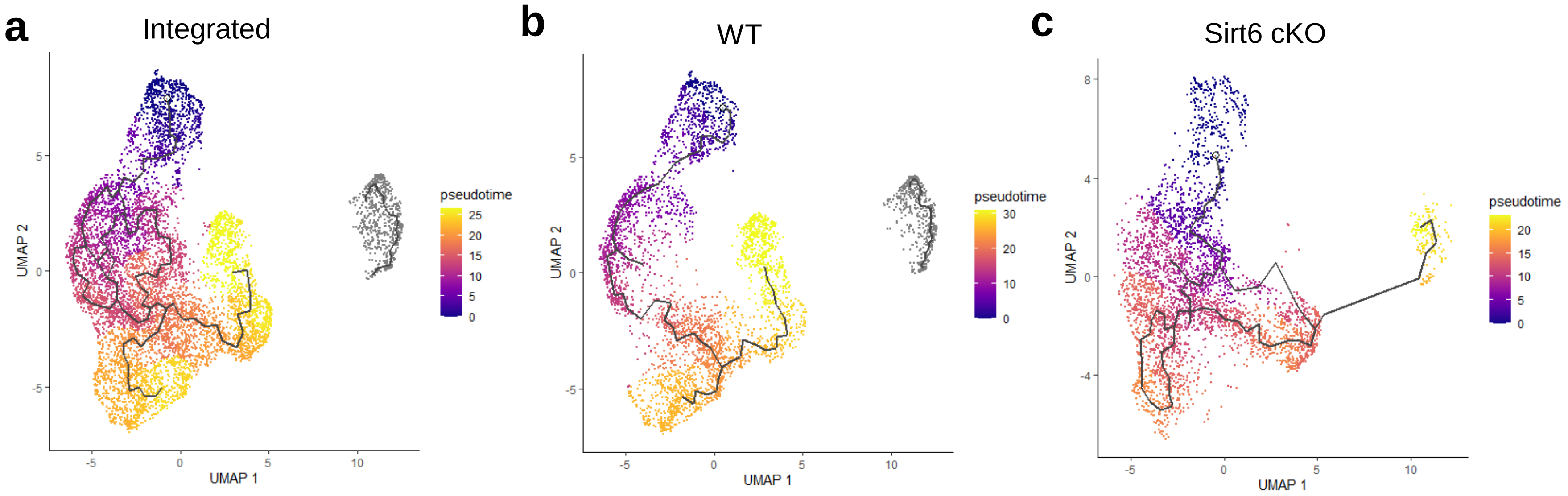

Supplementary Figure 31. Trajectory analysis of integrated scATAC-seq.
(**a**) UMAP visualization of integrated scATAC-seq trajectory colored based on the pseudotime. (**b**) UMAP visualization of WT cells in integrated scATAC-seq trajectory colored based on the pseudotime. (**c**) UMAP visualization of Sirt6 cKO cells in integrated scATAC-seq trajectory colored based on the pseudotime.

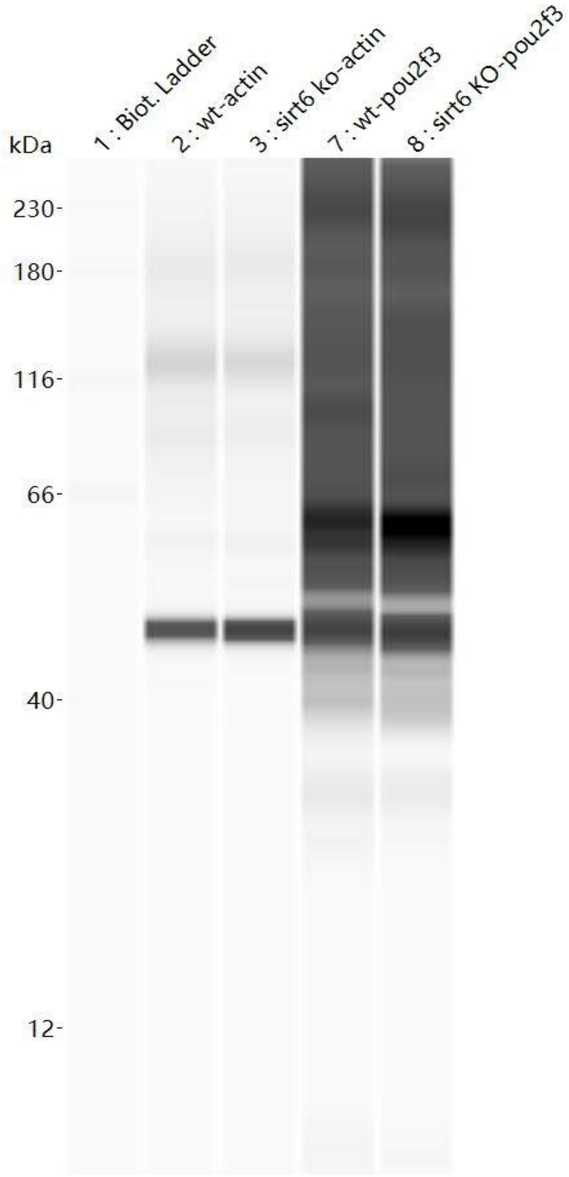

Supplementary Figure 32. The original picture of Simple western result for Fig 5c.

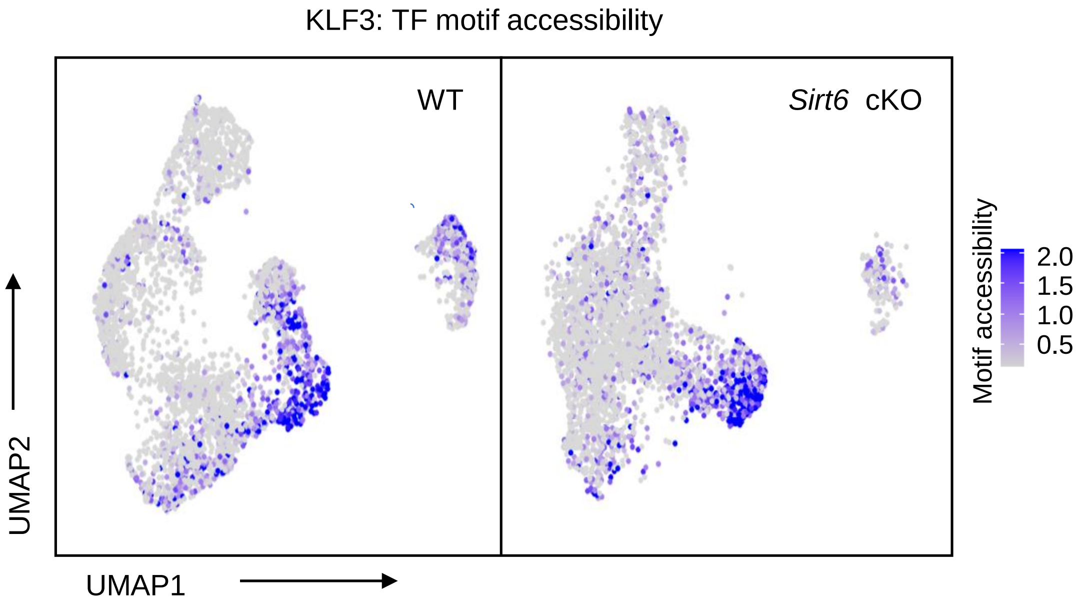

Supplementary Figure 33. Motif accessibility of KLF3 in WT and *Sirt6* cKO mTECs as analyzed by scATAC-seq.

KLF3-regulated motif were mainly opened in the Late Aire 2 subgroup, and *Sirt6* deletion did not impact their expression in Late Aire 2 cells. The blue scale in UMAP plot indicate the level of KLF3-regulated motif accessibility detected by scATAC-seq.

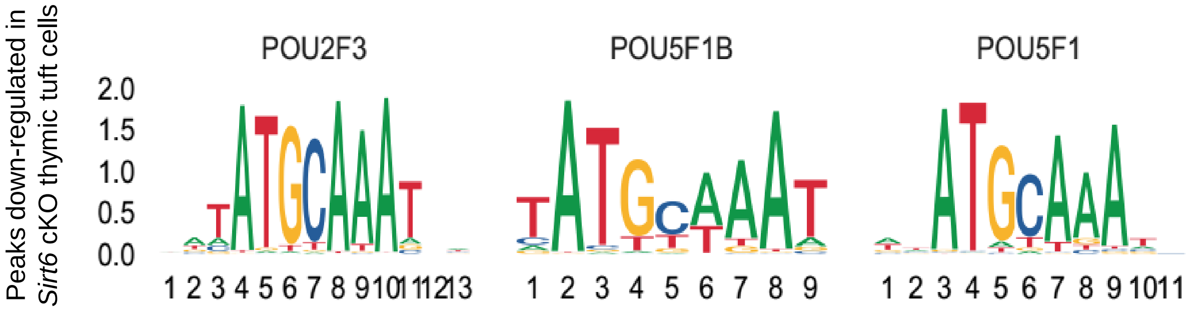

	POU2F3	POU5F1B	POU5F1
P-value	8.32e-289	3.44e-264	1.21e-264
% of target	49.87	48.13	42.16

Supplementary Figure 34. Transcription factor motif enrichment analysis of the down-regulated peaks in *Sirt*6 cKO thymic tuft cells.

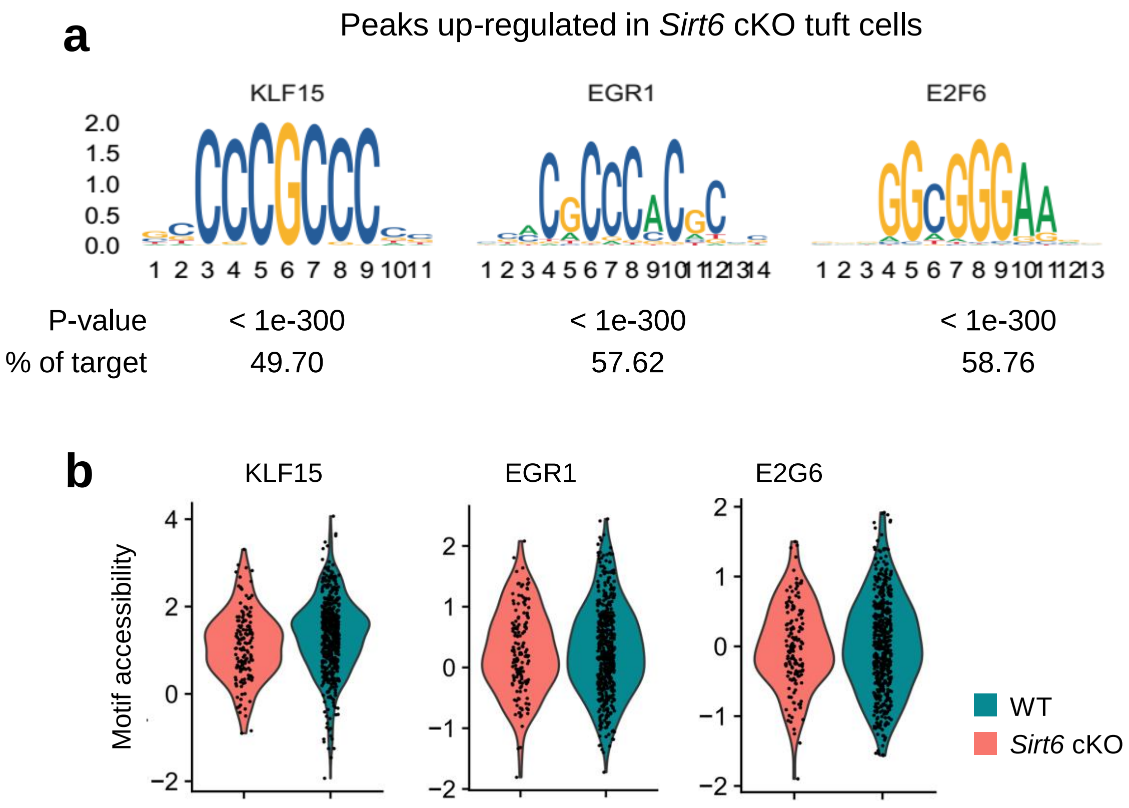

Supplementary Figure 35. Transcription factor motif enrichment analysis of the up-regulated peaks in *Sirt6* cKO thymic tuft cells.

(**a**) Position weight matrices of the motifs enriched by the upregulated peaks in *Sirt6* cKO mice. (**b**) Motif accessibility score of the motifs in WT and *Sirt6* cKO tuft cells determined by violin plot.

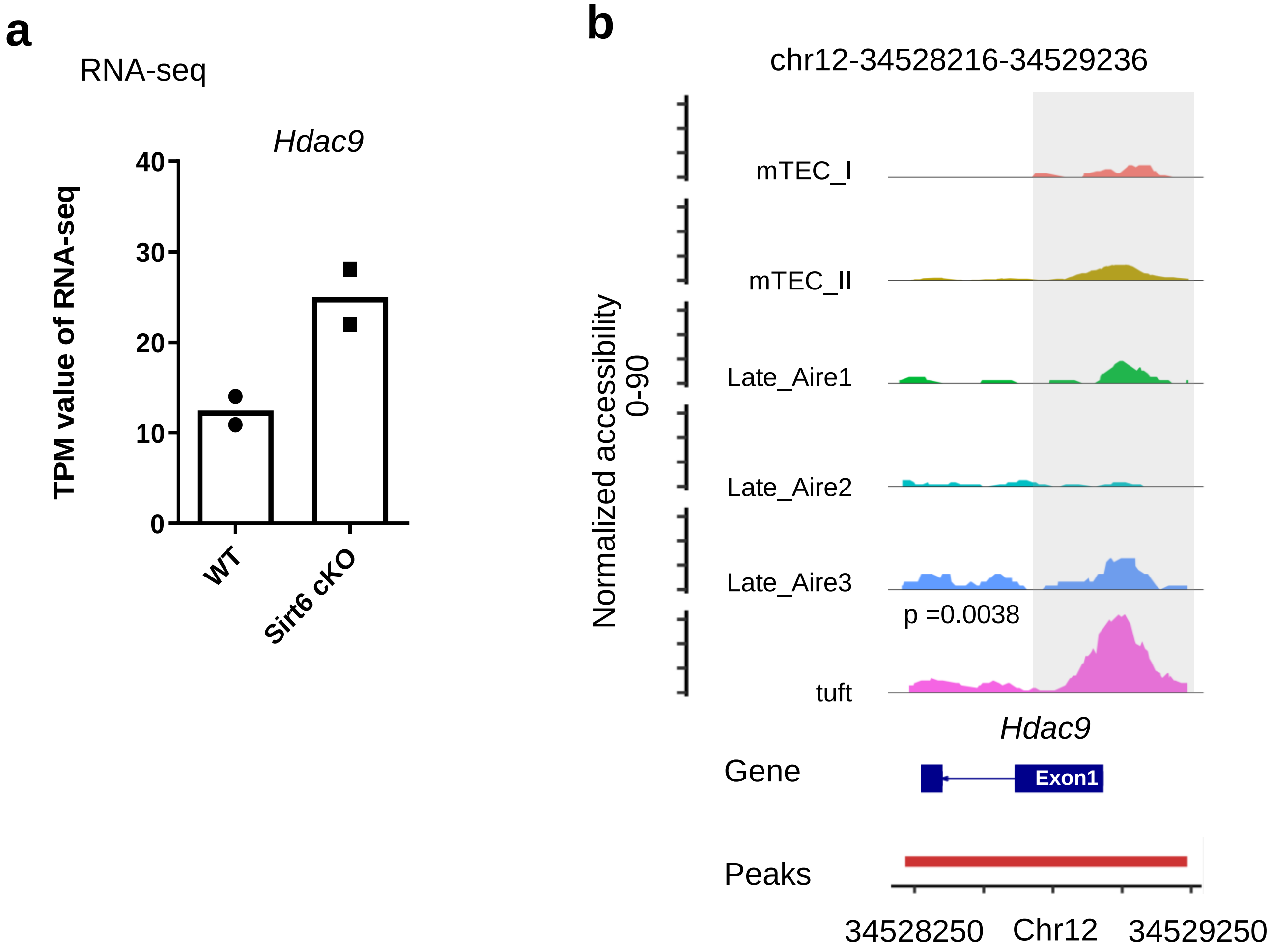

Supplementary Figure 36. *Hdac9* gene expression in mTECs assayed by scATAC-seq.
(**a**) The TPM value of *Hdac9* in WT and *Sirt6* cKO mTECs. (**b**) The chromatin accessibility of peaks around the promoter of *Hdac9* in all mTEC subsets are shown in WT scATAC-seq tracks. The colors in genome tracks indicate different cell types of mTECs. The arrow indicates the length and direction of the peak around the promoter region of Hdac9 detected by scATAC-seq.

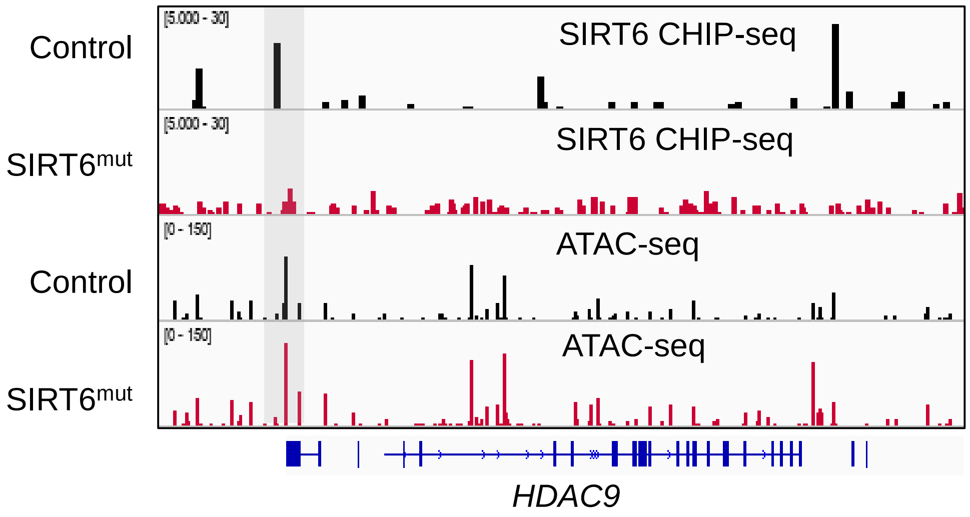

Supplementary Figure 37. Human *HDAC9* gene locus in WT and *SIRT6*-deficient SKMel-239 cell lines.

Capture of the UCSC (GRCh37/hg19) genome browser showing the human full *HDAC9* locus in *SIRT6* ChIP-seq and ATAC-seq of WT and *SIRT6*-deficient SKMel-239 cell lines. The arrow indicates the length and direction of the human HDAC9 gene.

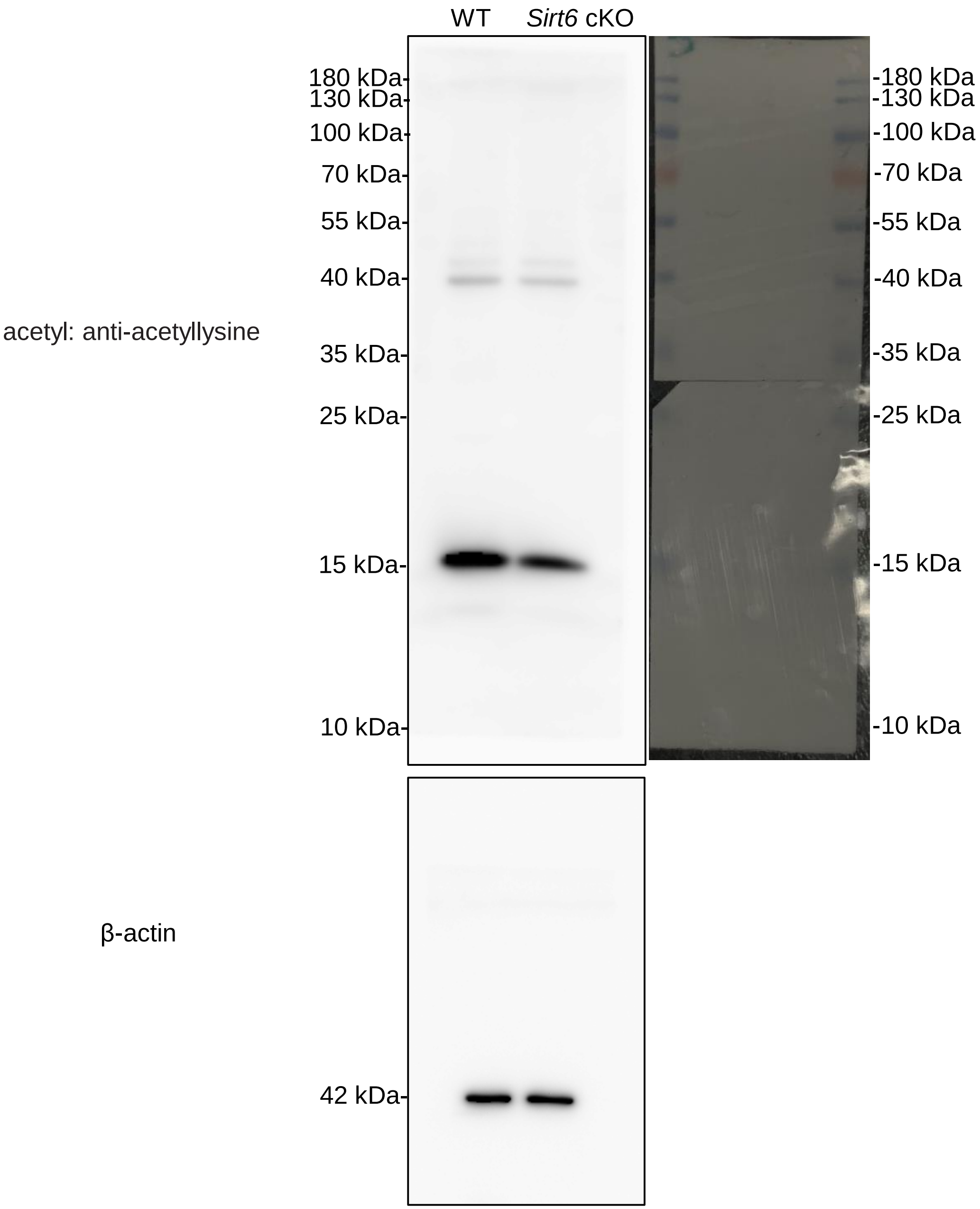

Supplementary Figure 38. The original picture of Western blot result for Fig 6g.
