# Supplementary material for: Sirt6-mediated epigenetic modification of DNA accessibility is essential for Pou2f3-induced thymic tuft cell development
Source: Commun Biol. 2022 Jun 6;5:544. doi: 10.1038/s42003-022-03484-9 (PMC9170729; doi:10.1038/s42003-022-03484-9)
Supplement: Supplementary file 2 — Description of Additional Supplementary Files [file 42003_2022_3484_MOESM2_ESM.pdf]

Description of Additional Supplementary Files

File name: Supplementary Data 1
Description: The source data behind the graphs in the Fig 1.

File name: Supplementary Data 2
Description: The source data behind the graphs in the Fig 2.

File name: Supplementary Data 3
Description: The source data behind the graphs in the Fig 3.

File name: Supplementary Data 4
Description: The genes regulated by Pou2f3.

File name: Supplementary Data 5
Description: The source data behind the graphs in the Fig 4.

File name: Supplementary Data 6
Description: The source data behind the graphs in the Fig 5.

File name: Supplementary Data 7
Description: The source data behind the graphs in the Fig 6.

File name: Supplementary Data 8
Description: Marker genes of mTEC subsets calculated by scATAC-seq.
