# Supplementary material for: Sirt6-mediated epigenetic modification of DNA accessibility is essential for Pou2f3-induced thymic tuft cell development
Source: Commun Biol. 2022 Jun 6;5:544. doi: 10.1038/s42003-022-03484-9 (PMC9170729; doi:10.1038/s42003-022-03484-9)
Supplement: Supplementary file 11 — Reporting Summary [file 42003_2022_3484_MOESM11_ESM.pdf]

nature portfolio

Corresponding author(s): Yong Zhao, COMMSBIO-21-2541-T

Last updated by author(s): Apr 8, 2022

Reporting Summary

Nature Portfolio wishes to improve the reproducibility of the work that we publish. This form provides structure for consistency and transparency in reporting. For further information on Nature Portfolio policies, see our Editorial Policies and the Editorial Policy Checklist.

Statistics

For all statistical analyses, confirm that the following items are present in the figure legend, table legend, main text, or Methods section.

n/a	Confirmed	
☐	☒	The exact sample size (*n*) for each experimental group/condition, given as a discrete number and unit of measurement
☐	☒	A statement on whether measurements were taken from distinct samples or whether the same sample was measured repeatedly
☐	☒	The statistical test(s) used AND whether they are one- or two-sided<br>*Only common tests should be described solely by name; describe more complex techniques in the Methods section.*
☐	☒	A description of all covariates tested
☐	☒	A description of any assumptions or corrections, such as tests of normality and adjustment for multiple comparisons
☐	☒	A full description of the statistical parameters including central tendency (e.g. means) or other basic estimates (e.g. regression coefficient) AND variation (e.g. standard deviation) or associated estimates of uncertainty (e.g. confidence intervals)
☐	☒	For null hypothesis testing, the test statistic (e.g. *F*, *t*, *r*) with confidence intervals, effect sizes, degrees of freedom and *P* value noted<br>*Give P values as exact values whenever suitable.*
☒	☐	For Bayesian analysis, information on the choice of priors and Markov chain Monte Carlo settings
☒	☐	For hierarchical and complex designs, identification of the appropriate level for tests and full reporting of outcomes
☐	☒	Estimates of effect sizes (e.g. Cohen's *d*, Pearson's *r*), indicating how they were calculated

Our web collection on statistics for biologists contains articles on many of the points above.

Software and code

Policy information about availability of computer code

Data collection

Cells were sorted by BD FACSAria™ Fusion Flow Cytometers (BD Biosciences, USA) for 10xGenomics scATAC-seq analysis. BD LSRFortessa X-20 Flow Cytometer (BD Biosciences, USA) were used for flow cytometry analysis. The scATAC-seq libraries was sequenced using an Illumina Novaseq sequencer with a sequencing depth of at least 25k read pairs per nucleus with pair-end 50 bp (PE50) reading strategy.
The gene expression matrix data of TEC scRNA-seq was obtained from GSE137699.
The analysis of interaction between SIRT6 and HDAC9 were performed by the ChIP-seq (SIRT6, H3K9ac, H3K27ac and H3K56ac) and ATAC-seq of WT and SIRT6 KO SKMel-239 cell line derived from GSE102813.
The bulk RNA-seq of WT and Sirt6 cKO mTEC were derived from our previously published studies, and the data was available on GSE166840.

Data analysis

scRNAseq analysis:
The downloaded scRNAseq data were analyzed using Seurat (v 4.0.1) R Package software. The marker genes of each cluster were generated by Seurat's default FindAllMarkers() function on the threshold of p_val_adj < 0.05 and avg_log2FC > 0.5.

scATAC-seq analysis:
The aggregated peak-by-cell data matrix was read into R (v 4.0.3) and processed using the Seurat (v 4.0.1) and Signac (v 1.1.1). Chromatin assay was constructed by "CreateChromatinAssay" and transfer to Seurat object by "CreateSeuratObject". The gene activity matrix was calculated for the filtered peak matrix by the Cicero analysis package (v 1.7.1) and the monocle 3 alpha package (2.99.3). Marker genes for peak-based clustering were generated using FindAllMarkers() function on the gene activity matrix base on the threshold of p_val_adj < 0.05 and avg_log2FC > 0.5. The shared characteristic genes between scATAC-seq and scRNA-seq calculated by scRNA-seq were added to peak-by-cell data matrix through AddModuleScore() function.
Motif enrichment analysis was performed using Signac (v 1.1.1) and ChromVAR (v 1.10.0). We create the Motif class to store all the required information, including a list of position weight matrices (PWMs) or position frequency matrices (PFMs) and a motif occurrence matrix derived from JASPAR2020 (species: 9606) database using AddMotifs() function.
Pseudotime analysis for the WT scATAC-seq analysis was performed by Monocle3 (v 0.2.1).

RNA-seq analysis:
The differentially expressed genes were calculated by DEseq2(v 1.28.1) package according to the threshold of adjusted p < 0.05 and |Log2FC| > 2.
The KEGG analysis of DEGs were performed on the web of KOBAS 3.0.

ChIP-seq analysis:
Reads were trimmed for Illumina adapter sequences using trim_galore (v 0.5.0) and aligned to the mm10 using hisat2 (version 2.1.0). Pou2f3 significant peaks were identified using MACS2 (v 2.1.1) with parameters --nomodel --shift -50 --extsize 100 and matching Input control was used to call peaks.
ChIPpeakAnno(v 3.28.0) was used to annotate the peaks found by MACS2, matching the upstream and downstream 2Kb regions of genes in EnsDb.Mmusculus.v79 genome to obtain 2152 predicted genes.

More details are provided in the materials and methods.

For manuscripts utilizing custom algorithms or software that are central to the research but not yet described in published literature, software must be made available to editors and reviewers. We strongly encourage code deposition in a community repository (e.g. GitHub). See the Nature Portfolio guidelines for submitting code & software for further information.

Data

Policy information about availability of data

All manuscripts must include a data availability statement. This statement should provide the following information, where applicable:
- Accession codes, unique identifiers, or web links for publicly available datasets
- A description of any restrictions on data availability
- For clinical datasets or third party data, please ensure that the statement adheres to our policy

The gene expression matrix data of TEC scRNA-seq was obtained from GSE137699. The analysis of interaction between SIRT6 and HDAC9 were performed by the ChIP-seq (SIRT6, H3K9ac, H3K27ac and H3K56ac) and ATAC-seq of WT and SIRT6 KO SKMel-239 cell line derived from GSE102813. The bulk RNA-seq of WT and Sirt6 cKO mTEC were derived from our previously published studies, and the data was available on GSE166840. The genes regulated by POU2F3 were calculated by the ChIP-seq (POU2F3) of intestinal tuft derived from GSE116510. The raw data of scATAC-seq sequencing using in this article have been deposited at the Big Data (BIGD) BioProject under number: PRJCA006664.

Field-specific reporting

Please select the one below that is the best fit for your research. If you are not sure, read the appropriate sections before making your selection.

☒ Life sciences ☐ Behavioural & social sciences ☐ Ecological, evolutionary & environmental sciences

For a reference copy of the document with all sections, see nature.com/documents/nr-reporting-summary-flat.pdf

Life sciences study design

All studies must disclose on these points even when the disclosure is negative.

Sample size	We choose Student's t-test (a method of testing hypotheses about the mean of a small sample drawn from a normally distributed population when the population standard deviation is unknown) for Statistical analysis, and a sample of size is smaller than 30. Therefore, we select n ≥ 3 according to the requirements of general biological replicates.
Data exclusions	Due to the extremely low number of thymic tuft cells in Sirt6 cKO mTECs, so we added more sorted Sirt6 cKO tuft cells for scATAC-seq assays. To ensure that the cell proportion of the data was consistent with the true value in vivo, we randomly excluded 50% of the Sirt6 cKO tuft cells in some assays based on the added cell proportion.
Replication	All attempts at replication were successful.
Randomization	Foxn1-Cre Sirt6loxp/loxp mice and Foxn1-Cre negative littermate controls were randomly obtained by hybridization between Foxn1-Cre mouse strain and Sirt6loxp/loxp mouse strain.
Blinding	All the data were collected and analyzed in a non-blind manner because subjective measurements were not involved.

Behavioural & social sciences study design

All studies must disclose on these points even when the disclosure is negative.

Study description	*Briefly describe the study type including whether data are quantitative, qualitative, or mixed-methods (e.g. qualitative cross-sectional, quantitative experimental, mixed-methods case study).*
Research sample	*State the research sample (e.g. Harvard university undergraduates, villagers in rural India) and provide relevant demographic*

Research sample	*information (e.g. age, sex) and indicate whether the sample is representative. Provide a rationale for the study sample chosen. For studies involving existing datasets, please describe the dataset and source.*
Sampling strategy	*Describe the sampling procedure (e.g. random, snowball, stratified, convenience). Describe the statistical methods that were used to predetermine sample size OR if no sample-size calculation was performed, describe how sample sizes were chosen and provide a rationale for why these sample sizes are sufficient. For qualitative data, please indicate whether data saturation was considered, and what criteria were used to decide that no further sampling was needed.*
Data collection	*Provide details about the data collection procedure, including the instruments or devices used to record the data (e.g. pen and paper, computer, eye tracker, video or audio equipment) whether anyone was present besides the participant(s) and the researcher, and whether the researcher was blind to experimental condition and/or the study hypothesis during data collection.*
Timing	*Indicate the start and stop dates of data collection. If there is a gap between collection periods, state the dates for each sample cohort.*
Data exclusions	*If no data were excluded from the analyses, state so OR if data were excluded, provide the exact number of exclusions and the rationale behind them, indicating whether exclusion criteria were pre-established.*
Non-participation	*State how many participants dropped out/declined participation and the reason(s) given OR provide response rate OR state that no participants dropped out/declined participation.*
Randomization	*If participants were not allocated into experimental groups, state so OR describe how participants were allocated to groups, and if allocation was not random, describe how covariates were controlled.*

Ecological, evolutionary & environmental sciences study design

All studies must disclose on these points even when the disclosure is negative.

Study description	*Briefly describe the study. For quantitative data include treatment factors and interactions, design structure (e.g. factorial, nested, hierarchical), nature and number of experimental units and replicates.*
Research sample	*Describe the research sample (e.g. a group of tagged Passer domesticus, all Stenocereus thurberi within Organ Pipe Cactus National Monument), and provide a rationale for the sample choice. When relevant, describe the organism taxa, source, sex, age range and any manipulations. State what population the sample is meant to represent when applicable. For studies involving existing datasets, describe the data and its source.*
Sampling strategy	*Note the sampling procedure. Describe the statistical methods that were used to predetermine sample size OR if no sample-size calculation was performed, describe how sample sizes were chosen and provide a rationale for why these sample sizes are sufficient.*
Data collection	*Describe the data collection procedure, including who recorded the data and how.*
Timing and spatial scale	*Indicate the start and stop dates of data collection, noting the frequency and periodicity of sampling and providing a rationale for these choices. If there is a gap between collection periods, state the dates for each sample cohort. Specify the spatial scale from which the data are taken*
Data exclusions	*If no data were excluded from the analyses, state so OR if data were excluded, describe the exclusions and the rationale behind them, indicating whether exclusion criteria were pre-established.*
Reproducibility	*Describe the measures taken to verify the reproducibility of experimental findings. For each experiment, note whether any attempts to repeat the experiment failed OR state that all attempts to repeat the experiment were successful.*
Randomization	*Describe how samples/organisms/participants were allocated into groups. If allocation was not random, describe how covariates were controlled. If this is not relevant to your study, explain why.*
Blinding	*Describe the extent of blinding used during data acquisition and analysis. If blinding was not possible, describe why OR explain why blinding was not relevant to your study.*

Did the study involve field work? ☐ Yes ☐ No

Field work, collection and transport

Field conditions	*Describe the study conditions for field work, providing relevant parameters (e.g. temperature, rainfall).*
Location	*State the location of the sampling or experiment, providing relevant parameters (e.g. latitude and longitude, elevation, water depth).*
Access & import/export	*Describe the efforts you have made to access habitats and to collect and import/export your samples in a responsible manner and in compliance with local, national and international laws, noting any permits that were obtained (give the name of the issuing authority, the date of issue, and any identifying information).*
Disturbance	*Describe any disturbance caused by the study and how it was minimized.*

Reporting for specific materials, systems and methods

We require information from authors about some types of materials, experimental systems and methods used in many studies. Here, indicate whether each material, system or method listed is relevant to your study. If you are not sure if a list item applies to your research, read the appropriate section before selecting a response.

Materials & experimental systems

n/a	Involved in the study
☐	☒ Antibodies
☒	☐ Eukaryotic cell lines
☒	☐ Palaeontology and archaeology
☐	☒ Animals and other organisms
☒	☐ Human research participants
☒	☐ Clinical data
☒	☐ Dual use research of concern

Methods

n/a	Involved in the study
☒	☐ ChIP-seq
☐	☒ Flow cytometry
☒	☐ MRI-based neuroimaging

Antibodies

Antibodies used

Fixable Viability Dye eFluor™ 506 (eBioscience, 65-0866-18, 1:400), CD45-BUV395 (BD Biosciences, clone 30-F11, 564279, 1:400), CD326-PE/Cy7 (Biolegend, clone G8.8, 188216, 1:800), UEA-I (Vector Laboratories, FL-1061, 1:300), Ly51-AF647 (Biolegend, clone 6C3, 108312, 1:300 dilution), Ly51-BV786 (BD Biosciences, clone BP-1, 740882, 1:300 dilution), I-A/I-E-BV421 (Biolegend, clone M5/114.15.2, 107632, 1:400 dilution), CD80-PE (Biolegend, clone 16-10A1, 104708, 1:2000 dilution), CD80-BV650 (Biolegend, clone 16-10A1, 104732, 1:300 dilution), L1CAM-PE (R&D, clone 555, FAB5674P, 1:20 dilution), rabbit anti-DCLK1 (Abcam, ab31704, 1:15000 dilution), TCR-β-PE-Cy7 (Biolegend, clone H57-597, 109222, 1:500 dilution), mouse CD1d loaded R-PE label (Proimmune, E001-2A-G, 1:600 dilution), RORγt-BV421 (BD Biosciences, clone Q31-378, 562894, 1:200 dilution), PLZF-AF488 (eBioscience, clone Mags.21F7, 53-9320-80, 1:200 dilution), CD4-PE (Biolegend, clone GK1.5, 100408, 1:1600 dilution), CD8a-PE/Cy5 (Biolegend, clone 53-6.7, 100710, 1:1600 dilution), EOMES-AF488 (eBioscience, clone Dan11mag, 53-4875-80, 1:200 dilution), CD127-BV785 (Biolegend, clone A7R34, 135037, 1:200 dilution), GATA3-APC (Biolegend, clone 16E10A23, 653805, 1:100 dilution), T-Bet-PE (eBioscience, clone 4B10, 12-5825-82, 1:200 dilution); Lin--FITC: CD4-FITC (Biolegend, clone GK1.5, 100405, 1:1000 dilution); CD8-FITC (BD Biosciences, clone 53-6.7, 553031, 1:400 dilution); CD19-FITC (Biolegend, clone 1D3/CD19, 153404, 1:200 dilution); CD11c-AF488 (eBioscience, clone N418, 53-0114-82, 1:400 dilution); CD11b-FITC (eBioscience, clone M1/70, 11-0112-82, 1:500 dilution); F4/80-FITC (eBioscience, clone BM8, 11-4801-82, 1:500 dilution); TCRγδ-FITC (BD Biosciences, clone GL3, 553177, 1:200 dilution); NK1.1-FITC (eBioscience, clone PK136, 11-5941-81, 1:400 dilution); TER119-FITC (Biolegend, clone TER-119, 116206, 1:400 dilution); Alexa Fluor 647 AffiniPure donkey anti-Rabbit IgG (H+L) (Jackson ImmunoResearch Laboratories, 703-545-155, 1:600 dilution). Acetyl-Histone H3 (Lys9) (C5B11) Rabbit mAb 9649 (1:1000 dilution), Acetyl-Histone H3 (Lys18) (D8Z5H) Rabbit mAb 13998 (1:1000 dilution) and Acetyl-Histone H3 (Lys27) (D5E4) XP® Rabbit mAb 8173 (1:200 dilution), all from Acetyl-Histone H3 Antibody Sampler Kit (Cell Signaling Technology, 9927).

Chicken anti-KRT5 (Biolegend, 905901; clone Poly9059) diluted by 1:300 and rabbit anti-DCLK1 (Abcam, ab31704) diluted by 1:250. Alexa Fluor 488-conjugated donkey anti-Chicken IgG (H+L) (Jackson ImmunoResearch Laboratories, 703-545-155) diluted by 1:300, Alexa Fluor 594-conjugated donkey anti-rabbit IgG (H+L) (Jackson ImmunoResearch Laboratories, 711-586-152) diluted by 1:300.

POU2F3 (Prosci, 7795) and anti-rabbit secondary HRP antibody (Proteinsimple, 042-206); Anti-acetyllysine mouse mAb (PTM BIOLABS, clone Kac-01, PTM0101) diluted by 1:1000 and anti-ACTIN (Sigma-Aldrich, A5441) diluted by 1:40000.

Validation

All primary antibody for flow cytometry and immunofluorescence staining used in this manuscript went through stringent validation steps as previously described (1. Zhang, Q. et al. Sirt6 Regulates the Development of Medullary Thymic Epithelial Cells and Contributes to the Establishment of Central Immune Tolerance. Front Cell Dev Biol 9, 655552; 2. Miller, C. N. et al. Thymic tuft cells promote an IL-4-enriched medulla and shape thymocyte development. Nature 559, 627-631; 3. Bornstein, C. et al. Single-cell mapping of the thymic stroma identifies IL-25-producing tuft epithelial cells. Nature 559, 622-626.) Optimal concentration was defined by comparing the expression with other previously validated clones of a given antibody.

For Western blot assay, the optimal concentration of primary antibody was defined according to the instructions on manufacturers' websites.

Eukaryotic cell lines

Policy information about cell lines

Cell line source(s)

State the source of each cell line used.

Authentication

Describe the authentication procedures for each cell line used OR declare that none of the cell lines used were authenticated.

Mycoplasma contamination

Confirm that all cell lines tested negative for mycoplasma contamination OR describe the results of the testing for mycoplasma contamination OR declare that the cell lines were not tested for mycoplasma contamination.

Commonly misidentified lines
(See ICLAC register)

Name any commonly misidentified cell lines used in the study and provide a rationale for their use.

Palaeontology and Archaeology

Specimen provenance	*Provide provenance information for specimens and describe permits that were obtained for the work (including the name of the issuing authority, the date of issue, and any identifying information). Permits should encompass collection and, where applicable, export.*
Specimen deposition	*Indicate where the specimens have been deposited to permit free access by other researchers.*
Dating methods	*If new dates are provided, describe how they were obtained (e.g. collection, storage, sample pretreatment and measurement), where they were obtained (i.e. lab name), the calibration program and the protocol for quality assurance OR state that no new dates are provided.*

☐ Tick this box to confirm that the raw and calibrated dates are available in the paper or in Supplementary Information.

Ethics oversight	*Identify the organization(s) that approved or provided guidance on the study protocol, OR state that no ethical approval or guidance was required and explain why not.*

Note that full information on the approval of the study protocol must also be provided in the manuscript.

Animals and other organisms

Policy information about studies involving animals; ARRIVE guidelines recommended for reporting animal research

Laboratory animals	Foxn1-Cre mouse strain was crossed with Sirt6loxp/loxp mouse strain to generate Foxn1-Cre Sirt6loxp/loxp mice and Foxn1-Cre negative littermate controls; No gender requirements; 4-week-old.
Wild animals	The study did not involve Wild animals.
Field-collected samples	The study did not involve samples collected from the field.
Ethics oversight	Mice were maintained in Institute of Zoology, Chinese Academy of Sciences (IOZ, CAS) specific pathogen–free conditions and treated in accordance with Animal Experiments Guidelines of the animal Ethics Committee of Institute of Zoology, Beijing, China.

Note that full information on the approval of the study protocol must also be provided in the manuscript.

Human research participants

Policy information about studies involving human research participants

Population characteristics	*Describe the covariate-relevant population characteristics of the human research participants (e.g. age, gender, genotypic information, past and current diagnosis and treatment categories). If you filled out the behavioural & social sciences study design questions and have nothing to add here, write "See above."*
Recruitment	*Describe how participants were recruited. Outline any potential self-selection bias or other biases that may be present and how these are likely to impact results.*
Ethics oversight	*Identify the organization(s) that approved the study protocol.*

Note that full information on the approval of the study protocol must also be provided in the manuscript.

Clinical data

Policy information about clinical studies
All manuscripts should comply with the ICMJE guidelines for publication of clinical research and a completed CONSORT checklist must be included with all submissions.

Clinical trial registration	*Provide the trial registration number from ClinicalTrials.gov or an equivalent agency.*
Study protocol	*Note where the full trial protocol can be accessed OR if not available, explain why.*
Data collection	*Describe the settings and locales of data collection, noting the time periods of recruitment and data collection.*
Outcomes	*Describe how you pre-defined primary and secondary outcome measures and how you assessed these measures.*

Dual use research of concern

Policy information about dual use research of concern

Hazards

Could the accidental, deliberate or reckless misuse of agents or technologies generated in the work, or the application of information presented in the manuscript, pose a threat to:

No	Yes	
☐	☐	Public health
☐	☐	National security
☐	☐	Crops and/or livestock
☐	☐	Ecosystems
☐	☐	Any other significant area

Experiments of concern

Does the work involve any of these experiments of concern:

No	Yes	
☐	☐	Demonstrate how to render a vaccine ineffective
☐	☐	Confer resistance to therapeutically useful antibiotics or antiviral agents
☐	☐	Enhance the virulence of a pathogen or render a nonpathogen virulent
☐	☐	Increase transmissibility of a pathogen
☐	☐	Alter the host range of a pathogen
☐	☐	Enable evasion of diagnostic/detection modalities
☐	☐	Enable the weaponization of a biological agent or toxin
☐	☐	Any other potentially harmful combination of experiments and agents

ChIP-seq

Data deposition

☐ Confirm that both raw and final processed data have been deposited in a public database such as GEO.

☐ Confirm that you have deposited or provided access to graph files (e.g. BED files) for the called peaks.

Data access links<br>*May remain private before publication.*	*For "Initial submission" or "Revised version" documents, provide reviewer access links. For your "Final submission" document, provide a link to the deposited data.*
Files in database submission	*Provide a list of all files available in the database submission.*
Genome browser session<br>(e.g. UCSC)	*Provide a link to an anonymized genome browser session for "Initial submission" and "Revised version" documents only, to enable peer review. Write "no longer applicable" for "Final submission" documents.*

Methodology

Replicates	*Describe the experimental replicates, specifying number, type and replicate agreement.*
Sequencing depth	*Describe the sequencing depth for each experiment, providing the total number of reads, uniquely mapped reads, length of reads and whether they were paired- or single-end.*
Antibodies	*Describe the antibodies used for the ChIP-seq experiments; as applicable, provide supplier name, catalog number, clone name, and lot number.*
Peak calling parameters	*Specify the command line program and parameters used for read mapping and peak calling, including the ChIP, control and index files used.*
Data quality	*Describe the methods used to ensure data quality in full detail, including how many peaks are at FDR 5% and above 5-fold enrichment.*
Software	*Describe the software used to collect and analyze the ChIP-seq data. For custom code that has been deposited into a community repository, provide accession details.*

Flow Cytometry

Plots

Confirm that:

☒ The axis labels state the marker and fluorochrome used (e.g. CD4-FITC).

☒ The axis scales are clearly visible. Include numbers along axes only for bottom left plot of group (a 'group' is an analysis of identical markers).

☒ All plots are contour plots with outliers or pseudocolor plots.

☒ A numerical value for number of cells or percentage (with statistics) is provided.

Methodology

Sample preparation	Thymi from 4-week-old wild-type and Sirt6 cKO mice were put into 6cm dishes respectively, then chopped into small pieces with surgical scissors and suspended in the DMEM (Hyclone Laboratories, SH30022.01B) medium containing 2% fetal bovine serum (FBS; Gibco, 16000-044). After removing most thymocytes from the supernatant, the remaining thymus fragments were digested with a solution containing 20 mg/ml collagenase/dispase (sigma Aldrich, 11097113001) and 20 U/ml DNase I (sigma Aldrich, d5025) for 40 minutes in a water bath at 37 ?. When the small pieces disappeared, the cell suspension filtered through a 200 mesh filter to obtain a single cell suspension. The cell suspension mentioned above was incubated with CD45 Microbeads (Miltenyi, mouse, 130-052-301) in dark for 15 minutes on ice, and the CD45 negative cells were enriched by LS column (Miltenyi, 130-042-401). After incubation with antibodies, wild-type samples (150000 CD45-CD326+ TECs) and Sirt6 cKO samples (100000 CD45-CD326+ TECs plus 8400 CD45-CD326+L1CAM+MHC IIlow thymic tuft cells) were sorted by BD FACSAria™ Fusion Flow Cytometers (BD Biosciences, USA) for 10xGenomics scATAC-seq analysis.
Instrument	BD FACSAria™ Fusion Flow Cytometers (BD Biosciences, USA);BD LSRFortessa X-20 Flow Cytometer (BD Biosciences, USA); Laser scanning confocal microscope (Zeiss LSM710, Oberkochen, Germany); WES (ProteinSimple, USA);
Software	Flowjo
Cell population abundance	The purity of the post-sort cells was more than 95% by flow cytometry.
Gating strategy	Thymic tuft cells (CD45–EpCAM+MHC IIlowDCLK1high) or (CD45–EpCAM+MHC IIlowL1CAM+); thymic iNKT (TCRβintCD1d+); NKT1 (TCRβintCD1d+PLZF–RORγt–), NKT2 (TCRβintCD1d+PLZF+RORγt–) and NKT17 (TCRβintCD1d+PLZF–RORγt+); EOMES +TCRβ+CD8 single-positive thymocytes; thymic ILCs (Lin–TCR–CD127+); ILC2 (Lin–TCR–CD127+Gata3+Rorγt–); mTEClo (CD45-EpCAM+UEA-1+CD80-); The boundaries between "positive" and "negative" staining cell populations are defined according to the known paper, 1. Zhang, Q. et al. Sirt6 Regulates the Development of Medullary Thymic Epithelial Cells and Contributes to the Establishment of Central Immune Tolerance. Front Cell Dev Biol 9, 655552; 2. Miller, C. N. et al. Thymic tuft cells promote an IL-4-enriched medulla and shape thymocyte development. Nature 559, 627-631; 3. Bornstein, C. et al. Single-cell mapping of the thymic stroma identifies IL-25-producing tuft epithelial cells. Nature 559, 622-626.

☒ Tick this box to confirm that a figure exemplifying the gating strategy is provided in the Supplementary Information.

Magnetic resonance imaging

Experimental design

Design type	*Indicate task or resting state; event-related or block design.*
Design specifications	*Specify the number of blocks, trials or experimental units per session and/or subject, and specify the length of each trial or block (if trials are blocked) and interval between trials.*
Behavioral performance measures	*State number and/or type of variables recorded (e.g. correct button press, response time) and what statistics were used to establish that the subjects were performing the task as expected (e.g. mean, range, and/or standard deviation across subjects).*

Acquisition

Imaging type(s)	*Specify: functional, structural, diffusion, perfusion.*
Field strength	*Specify in Tesla*
Sequence & imaging parameters	*Specify the pulse sequence type (gradient echo, spin echo, etc.), imaging type (EPI, spiral, etc.), field of view, matrix size, slice thickness, orientation and TE/TR/flip angle.*
Area of acquisition	*State whether a whole brain scan was used OR define the area of acquisition, describing how the region was determined.*

Diffusion MRI ☐ Used ☐ Not used

Preprocessing

Preprocessing software	*Provide detail on software version and revision number and on specific parameters (model/functions, brain extraction, segmentation, smoothing kernel size, etc.).*
Normalization	*If data were normalized/standardized, describe the approach(es): specify linear or non-linear and define image types used for transformation OR indicate that data were not normalized and explain rationale for lack of normalization.*
Normalization template	*Describe the template used for normalization/transformation, specifying subject space or group standardized space (e.g. original Talairach, MNI305, ICBM152) OR indicate that the data were not normalized.*
Noise and artifact removal	*Describe your procedure(s) for artifact and structured noise removal, specifying motion parameters, tissue signals and physiological signals (heart rate, respiration).*
Volume censoring	*Define your software and/or method and criteria for volume censoring, and state the extent of such censoring.*

Statistical modeling & inference

Model type and settings	*Specify type (mass univariate, multivariate, RSA, predictive, etc.) and describe essential details of the model at the first and second levels (e.g. fixed, random or mixed effects; drift or auto-correlation).*
Effect(s) tested	*Define precise effect in terms of the task or stimulus conditions instead of psychological concepts and indicate whether ANOVA or factorial designs were used.*

Specify type of analysis: ☐ Whole brain ☐ ROI-based ☐ Both

Statistic type for inference<br>(See Eklund et al. 2016)	*Specify voxel-wise or cluster-wise and report all relevant parameters for cluster-wise methods.*
Correction	*Describe the type of correction and how it is obtained for multiple comparisons (e.g. FWE, FDR, permutation or Monte Carlo).*

Models & analysis

n/a	Involved in the study
☐	☐ Functional and/or effective connectivity
☐	☐ Graph analysis
☐	☐ Multivariate modeling or predictive analysis

Functional and/or effective connectivity	*Report the measures of dependence used and the model details (e.g. Pearson correlation, partial correlation, mutual information).*
Graph analysis	*Report the dependent variable and connectivity measure, specifying weighted graph or binarized graph, subject- or group-level, and the global and/or node summaries used (e.g. clustering coefficient, efficiency, etc.).*
Multivariate modeling and predictive analysis	*Specify independent variables, features extraction and dimension reduction, model, training and evaluation metrics.*
